# d-StructMAn: Containerized structural annotation on the scale from genetic variants to whole proteomes

**DOI:** 10.1093/gigascience/giac086

**Published:** 2022-09-20

**Authors:** Alexander Gress, Sanjay K Srikakulam, Sebastian Keller, Vasily Ramensky, Olga V Kalinina

**Affiliations:** Helmholtz Institute for Pharmaceutical Research Saarland (HIPS)/Helmholtz Centre for Infection Research (HZI), Saarbrücken 8: 66123, Germany; Graduate School of Computer Science, Saarland University, Saarbrücken 5: 101990, Germany; Helmholtz Institute for Pharmaceutical Research Saarland (HIPS)/Helmholtz Centre for Infection Research (HZI), Saarbrücken 8: 66123, Germany; Graduate School of Computer Science, Saarland University, Saarbrücken 5: 101990, Germany; Interdisciplinary Graduate School of Natural Product Research, Saarland University, Saarbrücken 6: 119991, Germany; Helmholtz Institute for Pharmaceutical Research Saarland (HIPS)/Helmholtz Centre for Infection Research (HZI), Saarbrücken 8: 66123, Germany; Graduate School of Computer Science, Saarland University, Saarbrücken 5: 101990, Germany; Research Group Computational Biology, Max Planck Institute for Informatics, Saarbrücken 7: 66421, Germany; National Medical Research Center for Therapy and Preventive Medicine of the Ministry of Healthcare of Russian Federation, Moscow, Russia; Faculty of Bioengineering and Bioinformatics, Lomonosov Moscow State University, Moscow, Russia; Helmholtz Institute for Pharmaceutical Research Saarland (HIPS)/Helmholtz Centre for Infection Research (HZI), Saarbrücken 8: 66123, Germany; Medical Faculty, Saarland University, Homburg, Germany; Center for Bioinformatics, Saarland Informatics Campus, Saarbrücken, Germany

**Keywords:** Single-nucleotide variants, indels, genetic variation, protein structure, protein interactions, structural annotation, Docker container

## Abstract

**Background:**

Structural annotation of genetic variants in the context of intermolecular interactions and protein stability can shed light onto mechanisms of disease-related phenotypes. Three-dimensional structures of related proteins in complexes with other proteins, nucleic acids, or ligands enrich such functional interpretation, since intermolecular interactions are well conserved in evolution.

**Results:**

We present d-StructMAn, a novel computational method that enables structural annotation of local genetic variants, such as single-nucleotide variants and in-frame indels, and implements it in a highly efficient and user-friendly tool provided as a Docker container. Using d-StructMAn, we annotated several very large sets of human genetic variants, including all variants from ClinVar and all amino acid positions in the human proteome. We were able to provide annotation for more than 46% of positions in the human proteome representing over 60% proteins.

**Conclusions:**

d-StructMAn is the first of its kind and a highly efficient tool for structural annotation of protein-coding genetic variation in the context of observed and potential intermolecular interactions. d-StructMAn is readily applicable to proteome-scale datasets and can be an instrumental building machine-learning tool for predicting genotype-to-phenotype relationships.

Key Points

- A novel bioinformatics tool for structural characterization of genetic variants is presented.
- Single-nucleotide variants and indels are described with respect to intermolecular interactions in homologous protein complexes.
- An efficient implementation using a Docker container allows for analysis of large whole proteome-scale datasets.

## Introduction

### Background

In the age of next-generation sequencing, large-scale genetic diversity within populations became apparent. A single human individual of European ancestry carries around 3 million genetic variants [1], of which up to 14,000 occur in coding regions and lead to an amino acid substitution [2]. Additionally, up to 3,000 short insertions or deletions (indels) occur in the coding regions and either lead to a frame shift or cause an indel in the corresponding protein sequence [3]. These coding variants that result in nonsynonmous substitutions or indels in the protein sequence are the focus of this study. Additional sources of indels are alternatively spliced isoforms that result in retaining or skipping exons or parts of introns in the translated sequence. Alternative splicing is widespread across eukaryotes and human tissues [4]. UniProt lists an average of approximately 4 isoforms per a protein-coding gene in its reference human proteome.

Although most of sequence variants have no functional or pathogenic effect, in some cases, even a single mutation can be disease causing [5, 6]. Experimental characterization of all variants is infeasible; since most of them are rare and occur in only 1 or few individuals [7], statistical characterization of their association with diseases seems impossible, too. This emphasizes the importance of computational tools for predicting functional and/or pathogenic effects of sequence variants. Many such tools were developed specifically for nonsynonymous (missense) variants and take into account the protein 3-dimensional (3D) structure context of an input variant. One of the keystones in this field is PolyPhen [8, 9], which combines a number of features related to protein sequence and 3D structure in a Bayes classifier that predicts an individual variant to be deleterious or benign. Other methods also rely on conservation of the mutated position in a protein alignment [10, 11] or use additional features derived from phylogenetic analysis or analysis of the protein 3D structure [12–16]. Meta-methods combine outputs from several prediction tools [17–19].

Of those tools that employ protein 3D structure to derive various predictive features, several put variants of interest in the context of protein–protein interactions [20] and interactions with other biologically relevant molecules [21]. Indeed, features related to protein 3D structure were proven to be instrumental for predicting pathogenic effects of mutations [22]. Various intricate facets of structural analysis have also been implemented in databases and computational tools [23–26] with 1 common drawback: they rely on information from individual structures and do not integrate the multiple relevant findings.

As far as structural annotation of larger variants is concerned, most methods are developed to annotate single amino acid replacements, and to the best of our knowledge, there is currently no structural annotation pipeline that is able to map indel-type genetic variants to protein 3D structures.

### High-performance structural annotation

In this study, we present d-StructMAn, a new improved implementation of our earlier tool StructMAn [27], shipped in a convenient and easily installable container form and extended to annotation of short in-frame indels and alterations arising as a consequence of alternative splicing events. d-StructMAn produces a wide range of structural features by combining information from experimentally resolved structures of many related proteins, which is a unique feature of the StructMAn family tools. In addition to experimentally resolved protein 3D structures, d-StructMAn can also harness information from all protein structure models stored in the AlphaFold Protein Structure Database [28, 29]. To the best of our knowledge, this is the first tool with this property that can also analyze genomic indels and consequences of alternative splicing events. Additionally, we provide structural annotation of all proteins in the human proteome (all canonical protein sequences and all isoform sequences listed in UniProt) and all pathogenic and benign genetic variants from ClinVar [6] as a publicly available data resource.

## Data Description

### Human proteome dataset

The human proteome dataset contains 101,014 protein isoform sequences belonging to 79,038 human protein entries in UniProt [30]. We generated the dataset by downloading (on 5 December 2021) all sequences from https://www.uniprot.org/uniprot/?query=human&fil=proteome%3AUP000005640+AND+organism%3A%22Homo+sapiens+%28Human%29+%5B9606%5D%22&sort=score# [31] choosing for format the following: “FASTA (canonical & isoforms).” StructMAn can process this FASTA file directly. However, to test StructMAn’s functionality to retrieve sequences in an isoform-specific manner, we have also extracted accession identifiers and used them as an input. The resulting file (Supplementary File S1) represents protein sequence data over 4 million individual amino acids and was used as input file for StructMAn.

### ClinVar

The freely accessible ClinVar [6] database contains human mutations annotated with clinical outcomes. We downloaded (on 13 September 2021) the “variant_summary.txt.gz” from https://ftp.ncbi.nlm.nih.gov/pub/clinvar/tab_delimited/ [32]. We retained DelIns (multiresidue substitutions), insertions or deletions with “Assembly” field equal to GRCh38, and a RefSeq [33] protein identifier provided. The provided “clinical significance” field was simplified to Pathogenic, Benign, and Unknown. The resulting dataset is described in the Table 1 and Supplementary File S2.

**Table 1:** The ClinVar dataset

Mutation type	Pathogenic	Benign	None	Total
SAV*	41,557	48,285	317,261	407,103
Deletion	1,615	775	5,389	7,779
Insertion	108	82	372	562
DelIns**	287	46	750	1,083
All	43,567	49,188	323,772	416,527

*Single amino acid variation.

**Multiresidue substitutions.

## Analyses

### Annotation of the human proteome

The structural annotation of more than 100,000 protein sequences represents size-wise the ultimate challenge for a structural annotation method but, on the other hand, reviews the future applicability of the method by estimating what fraction of the human proteome can be mapped to the structure data.

**Figure 1:**
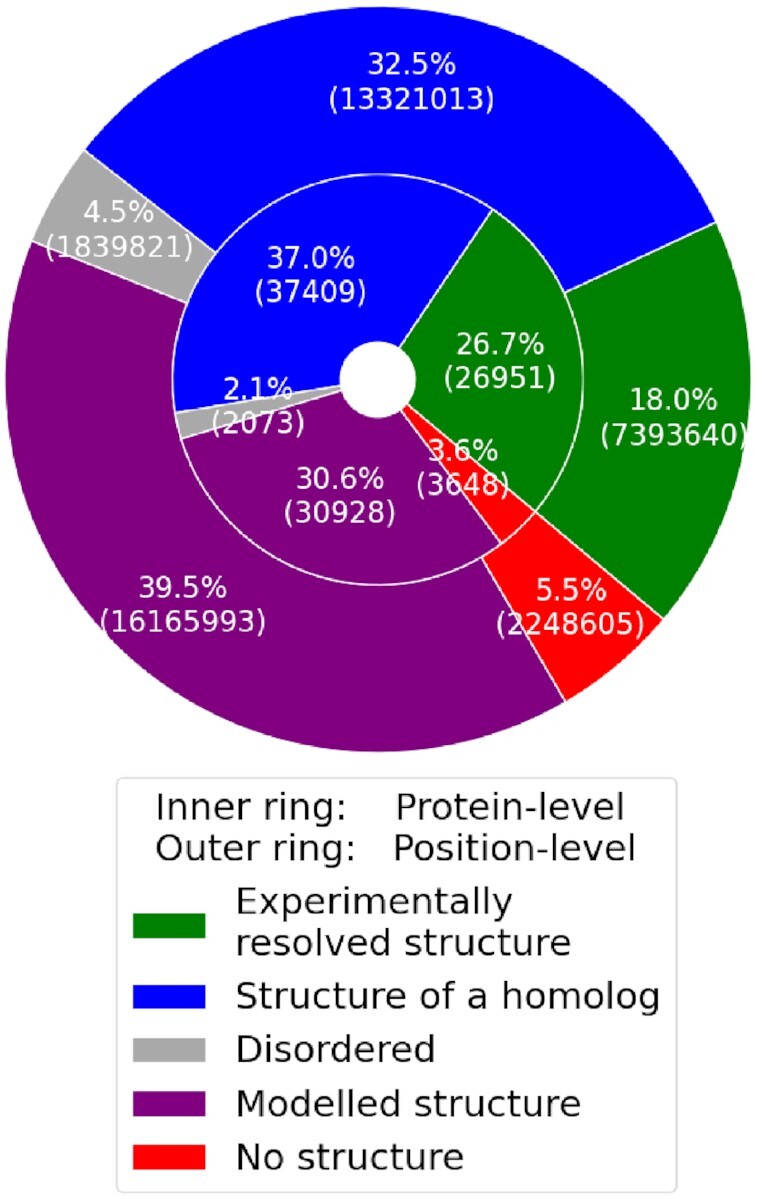
Proportion of proteins and positions from the human proteome dataset that could be mapped to structure data. Experimentally resolved structure (green) denotes that the protein (position) was mapped to at least 1 structure with sequence identity ≥0.99, and structure of a homolog (blue) denotes that the protein (position) was mapped to at least 1 structure with sequence identity in the range from 0.35 to 0.99. Modeled structure (purple) denotes that the protein (position) could only be mapped into a modeled structure that is not directly supported by experimental data. Disordered (gray) denotes proteins and positions that could not be mapped to any structure but are predicted by IUpred3 [34] to be disordered (for proteins, all positions have to be predicted to be disordered). No structure (red) denotes all other proteins and positions.

For only 21.7% of all proteins, the corresponding experimentally resolved structure is available in the Protein Data Bank (PDB) (Fig. 1), and since not all of them were resolved in full length, this estimate is reduced to 13.9% of the positions. Mapping proteins to structures of homologs drastically increases the usability of structural annotation to 60.5% of all proteins in the human proteome.

**Figure 2:**
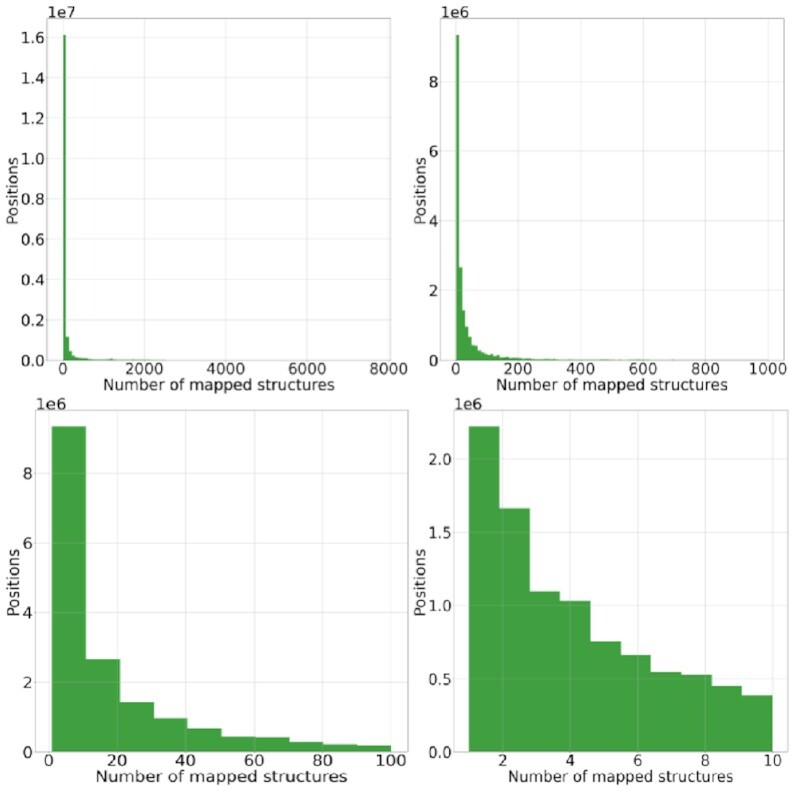
Distribution of the number of structures that could be mapped to a position in the annotation of the human proteome dataset. Each subplot shows the same distribution with a different zoom.

When a position can be structurally annotated, it can rarely be mapped to only structure. Most of the times, it is possible to identify more than 1 structure for a position that can be used for structural annotation (Fig. 2). Hence, another challenge of structural annotation is to manage the multiple sources of structural information. In such cases, StructMAn provides the user with recommendations listing the structure with the highest sequence identity and a structure considered to be the most representative biologically. The latter recommendation is based on the the structural analysis of all annotated structures and aggregation of relevant information. Sometimes, both structures (maximal identity and recommended) may be the same one.

**Figure 3:**
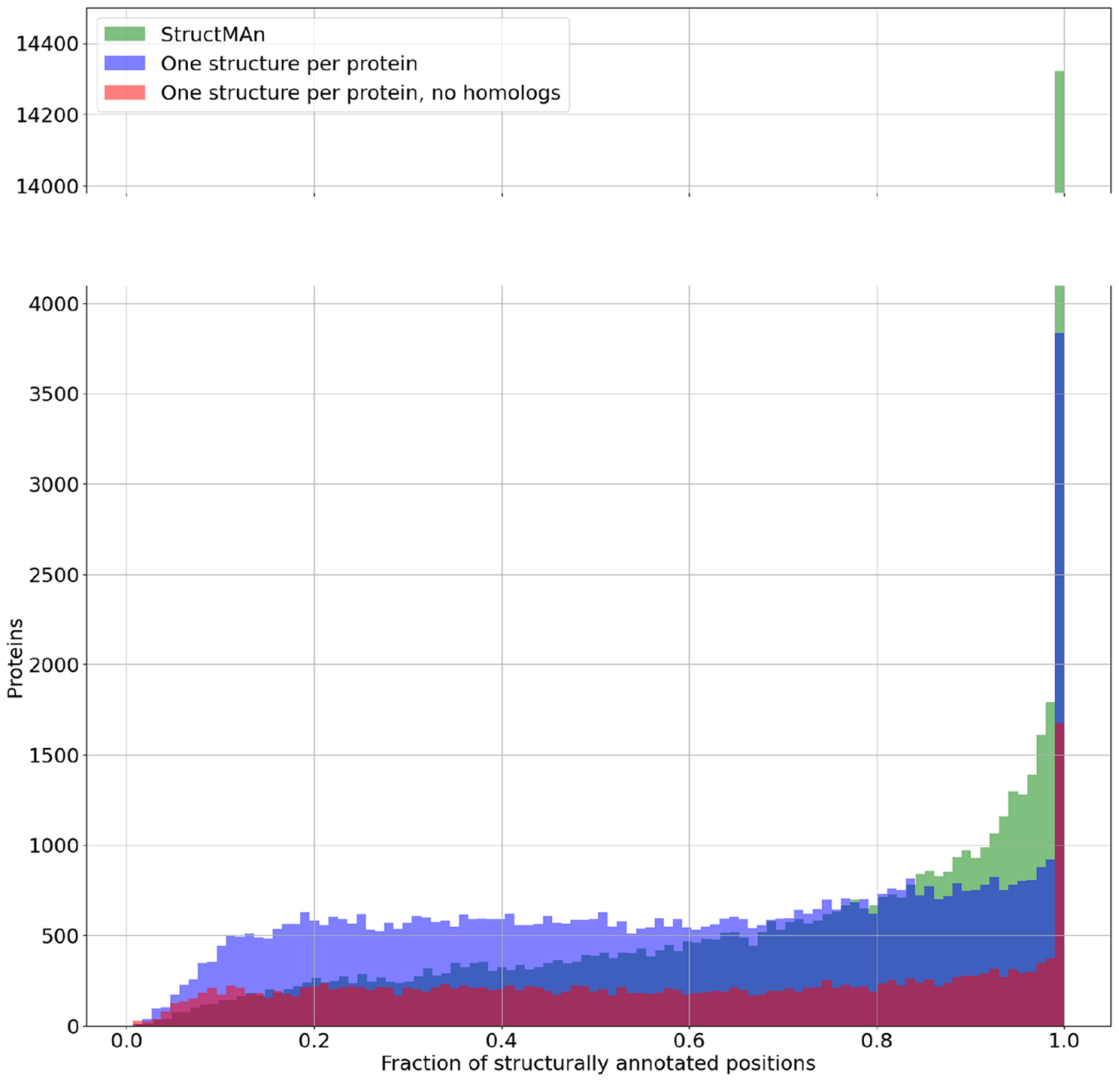
Distribution of the proportion of positions per protein that could be mapped to a structure in the annotation of the human proteome dataset. Green: fraction of positions annotated using multiple structures found by StructMAn; blue: fraction of positions annotated using only 1 structure per protein (highest sequence similarity was used, in case of same-sequence similarity; higher alignment coverage was preferred); red: fraction of positions annotated without considering structures of homologs.

Another advantage of annotation using all available structures is for proteins with partially resolved structures that cover different regions of the protein. This way, StructMAn could annotate 15% more (46% vs. 31%) positions compared to a strategy, where only the structure with maximum sequence identity is chosen (Fig. 3).

### Clinically relevant genetic variations

ClinVar contains genetic variations labeled with their clinical outcome and is routinely used in numerous supervised machine learning methods aimed to predict the effects of genetic variations [22, 35–37]. This makes ClinVar the ideal testing ground to showcase the feature generation capabilities of StructMAn. It also demonstrates the new indel annotation and analyses that generated features specific to indel-type genetic variations.

**Figure 4:**
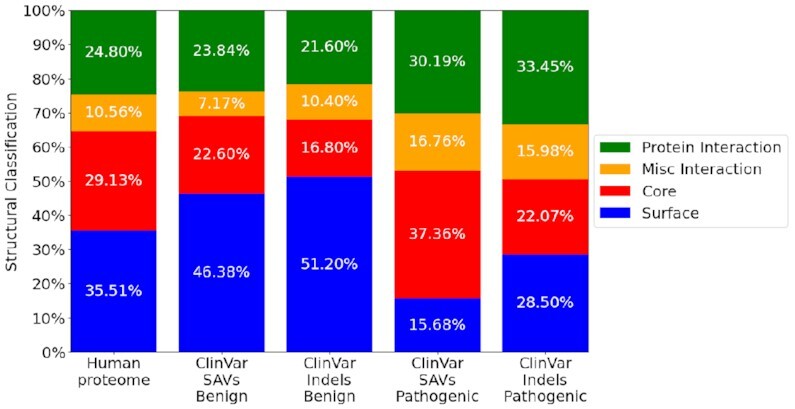
Each stacked barplot denotes the distribution of structural classifications for a dataset. Only positions that could be mapped to at least 1 structure are considered for this figure. Protein interaction: amino acids that are part of a protein–protein interaction interface; Misc interaction: amino acids that are part of an interaction with a nonprotein partner (DNA, for example); Core: amino acids in the core of the protein; Surface: amino acids classified to have access to solvent (and not involved in interactions).

We performed structural classification of all positions in the human proteome and variant positions in the ClinVar dataset (more details in the Methods section) and compared structural classes distribution depending on the ClinVar variant clinical significance (Fig. 4). Structural classifications for benign genetic variants, both single amino acid variations (SAVs) and indels, are distributed similarly to the classifications for all amino acids in the human proteome. Pathogenic SAVs have an increased tendency to be located in the protein core. This is also the case for pathogenic indels but not as strongly as for pathogenic SAVs. Further, pathogenic SAVs and indels are enriched on interaction interfaces. These results are in agreement with our earlier analysis [38]; for example, benign variants are enriched for noninteracting surfaces, whereas pathogenic and disease-associated variants are depleted in these regions but tend to appear more often on interaction interfaces.

**Figure 5:**
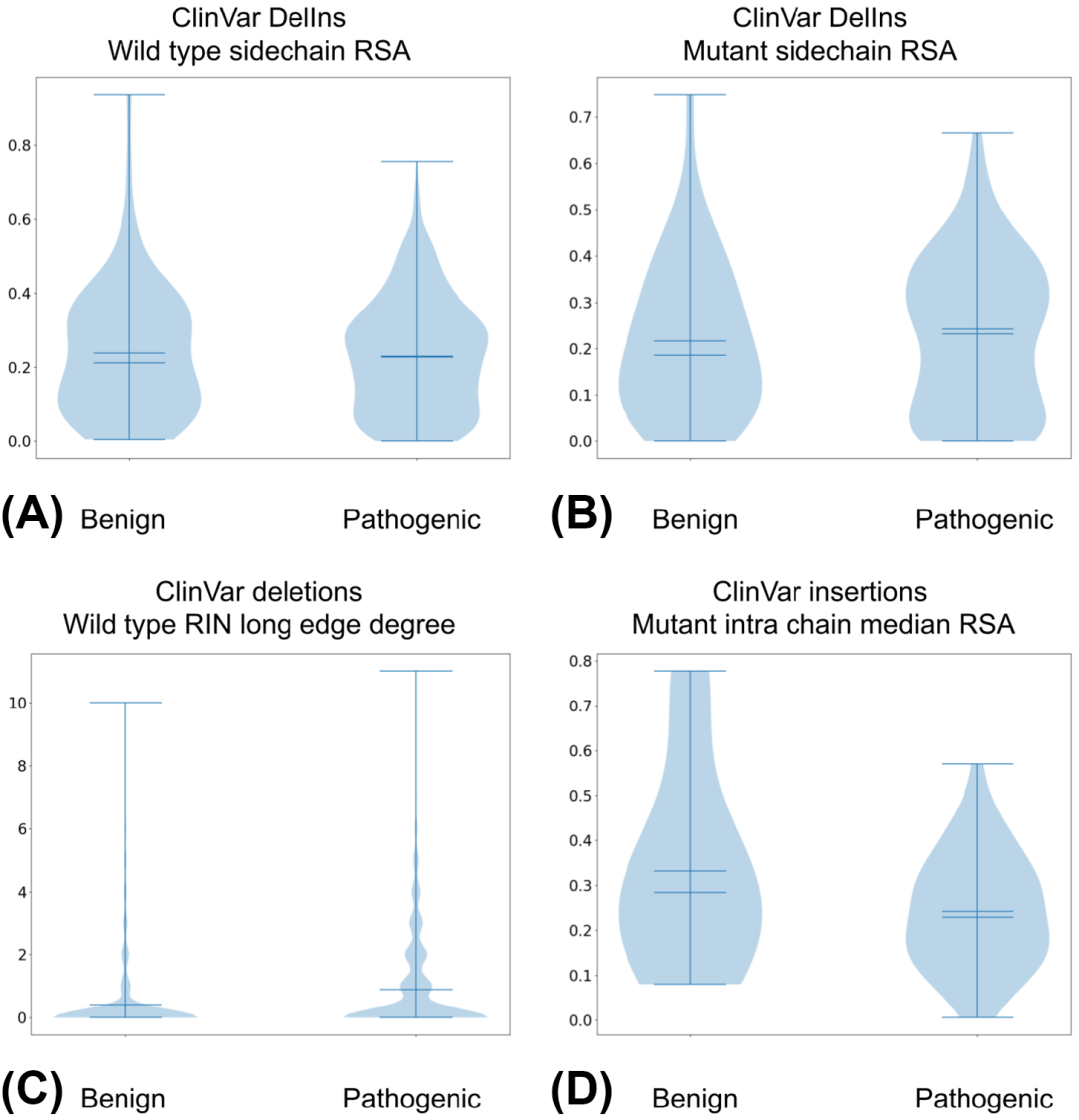
Violin plots for 4 example features. Left and right plots display the distribution of feature values for benign and pathogenic variants in ClinVar, respectively. (A) Relative surface area (RSA) value for chain atoms in the structures used for annotation of the wild-type protein. Only DelIns (multiresidue substitutions). (B) Same as A, but for the structures used for annotation of the mutant protein. (C) Only deletions. The number of spatial interactions to other amino acids in the same polypeptide chain and separated by more than 6 residues in the sequence. (D) Only insertions. Median solvent access of residues from other proteins (co-crystallized structures) that lie in a 10 Å sphere around the annotated residue.

Most missense variant effect prediction methods based on supervised machine learning models rely on ClinVar as the training and testing dataset. We used d-StructMAn to generate 123 features for every SAV and 600 features for every indel in ClinVar (more details about feature generation are provided in the Methods section: for SAVs, see section “Structural analysis of individual structures”; for indels, see section “Aggregation of annotation results for indels”). Structural features generated for SAVs are based on the results of the structural analysis of the residues in the wild-type, while for indels, the wild-type and mutant versions of the protein are analyzed separately and used for features generation (see Methods for more details). We selected 4 example features and plotted their value distributions for benign and pathogenic indels (Fig. 5). This gives an example of d-StructMAn employed for the preliminary feature analysis and selection, since a machine learning method would benefit from features that discriminate the datasets of interest.

### Performance benchmark

In order to benchmark the runtime performance of StructMAn on different computing systems and different configurations, we generated 6 datasets. Three of them contain 10, 50, or 100 proteins that all can be mapped to only 1 or very few PDB entries. Two other datasets contained 10 or 50 proteins that each could be mapped to around 100 PDB entries. The last dataset contains 10 proteins that are very challenging for the pipeline, since they can be mapped to over 13,000 PDB entries, since this dataset includes such proteins as kinases and antibodies. The total number of mapped structures and hence the total number of structurally analyzed residues is the main cause for computational complexity. This number does not directly depend on the number of proteins in the dataset (Table 2).

**Table 2:** The benchmark datasets. The first 3 datasets contain proteins that can be mapped to very few structures. The third and fourth datasets contain proteins that could be map to many structures (around 100). The last dataset contains 10 proteins that can be mapped to >13,000 of structures in total.

Dataset	Positions	Mapped	Analyzed
		PDB entries	residues
10 proteins, few hits	1,398	14	7,242
50 proteins, few hits	13,612	70	45,014
100 proteins, few hits	33,789	146	129,465
10 proteins, many hits	2,415	1,216	678,472
50 proteins, many hits	29,726	5,339	2,709,267
10 proteins, very many hits	1,440	13,109	9,753,815

**Figure 6:**
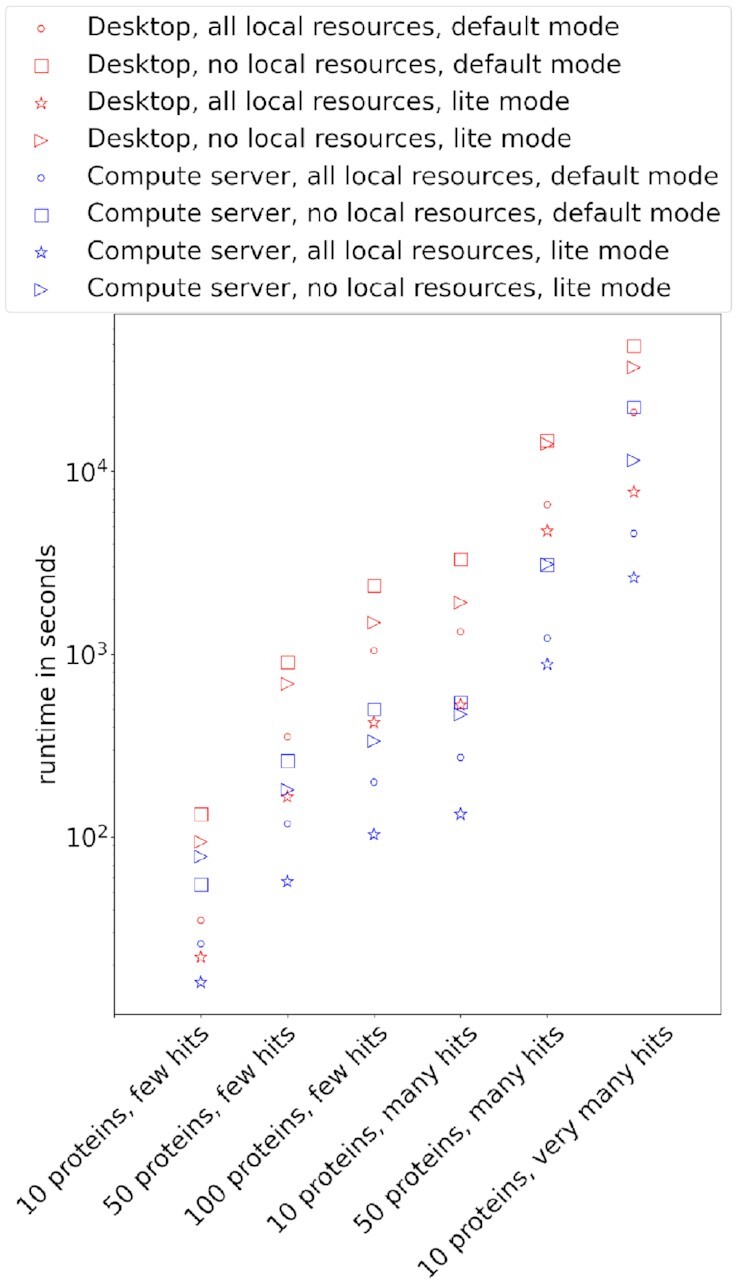
Scatterplot showing runtime performance of StructMAn using different systems and different configurations. Red markers denote a normal desktop computer and blue markers denote a high-performance computing server. Different marker shapes denote different configurations of d-StructMAn.

We processed the benchmark datasets on different systems and different configurations. First, these configurations differ in whether we used local resources (local instances of Uniprot and PDB; see Methods for more information). Second, they differ in whether we used the so-called lite mode that switches off the usage of the internal StructMAn database and performs all the calculations and retrievals on the fly. The lite mode still can use local resources but does not store intermediate results; thus, it is faster on smaller inputs but slower on larger inputs and when run multiple times in succession. All benchmark runs are conducted on an empty StructMAn database instance; thus, we only measure the overhead of filling the database and not the amortized benefits one would receive from successive usage of StructMAn, when many intermediate results can be reused. Therefore, lite mode runs always have an advantage over the default mode runs in this benchmark. Runtime was measured in 2 systems and 4 different configurations for each dataset (Fig. 6).

### Output example

d-StructMAn is able to produce very comprehensive outputs, and here we describe an example annotating 1 SAV D833A in the histone-lysine N-methyltransferase EHMT2 (Uniprot accession: A2ABF8).

#### Classification table

The classification table presents the integral output of the pipeline and is provided in a tab-separated (.tsv) file format. This table contains 28 values per queried position. The most informative of these values are summarized in Table 3 (we provide the full list of values in Supplementary Table S4).

**Table 3:** Condensed classification output for position D833 in A2ABF8

Weighted mainchain location	Core
Weighted sidechain location	Surface
RIN class	Multiple interactions: sidechain
	contact with a ligand and
	sidechain contact with a protein
Amount of structures	
this position is mapped to	291
Recommended structure	2V4H:C 118:D

From the selected values, one can see that the sidechain of the queried amino acid is part of the protein surface, while its mainchain belongs to the core of the protein. Further, it participates in interactions with low-molecular-weight ligands and other proteins. We can see that the example position was mapped to 291 different experimentally resolved protein 3D structures, and one of them was provided as recommended structure (including the corresponding chain identify and residue number).

#### Feature table

The feature table is also a tab-separated (.tsv) file containing 1 row per queried position. It contains all values computed during the structural analysis that could be used as features in a machine learning method. A manual interpretation of the feature table is much harder, and thus we focus on 3 specific feature values (Table 4) to get a deeper insight into the interactions the queried amino acid position engages in. The shown scores reflect the strength of interaction between mainchain atoms or sidechain atoms of the mutated position and different types of interaction partners. The given interaction score between sidechain atoms and low-molecular-weight ligands (0.00707) is relatively low and indicates that the protein–protein interaction is perhaps the more relevant type of interaction in this case. When comparing the protein interaction scores between sidechain atoms (0.02437) and mainchain atoms (0.01546), we can see that the interaction is mitigated more by the sidechain part of the amino acid, which agrees well with the observation that we made regarding surface/core orientation based on the classification table. The overall distribution of interaction scores over all considered proteins (data not shown) indicates that these scores are comparably low; thus, while the amino acid clearly participates in the interactions, it might not be the most important player for it.

**Table 4:** Three examples of feature table entries of protein A2ABF8 D833A

**Feature name**	**Value**
Sidechain ligand score	0.00707
Sidechain protein score	0.02437
Mainchain protein score	0.01546

## Discussion

In this study, we presented the structural annotation method d-StructMAn. To our knowledge, it is the first fully automated structural annotation method that can be installed locally and run as a command-line tool inside a Docker container. We annotated 2 big datasets with d-StructMAn: all proteins in human and ClinVar (the annotations are publicly available; see Availability of Source Code and Requirements section). The annotation of the human proteome showed that in practice, structural annotation is possible for more than 50% of positions, but only if the structural annotation method considers structures of homologs. A great advantage of d-StructMAn is the analysis of all available homologous protein structures in addition to aggregation of the results. These annotations will be useful for scientists in many practical application scenarios.

The performance benchmark confirmed that d-StructMAn runs well on personal laptop and desktop computers. However, for the annotation of large-scale datasets, we would still suggest using a compute server. For instance, the annotation of the human proteome took over 55 hours on our server, consuming 250 cores and 1,511 Gb RAM. The same system processed the most difficult test dataset in the benchmark section in 1.5 hours, while the desktop system (11 cores, 16 Gb RAM) took almost 6 hours.

## Potential implications

The feature vectors generated by d-StructMAn structural analysis for each given protein position are ideal to be fed into complex supervised machine learning methods. The history of the application of protein structure–based features in mutation effect prediction is surprisingly vacant. This is due to 2 major challenges: First, the computational work and the implementation needed to generate structural features cannot be underestimated. This hurdle should now be solved by our containerized structural annotation method. The second challenge is the sparsity of structural features, since they are not available for all positions in all proteins. Here, we implemented a variety of techniques to increase the coverage by considering structures of all homologous proteins and aggregating results from them. The gradual growth of the PDB size and the recent developments in the protein structure prediction [28] also help to overcome the problem of incomplete protein sequence space coverage.

## Methods

**Figure 7:**
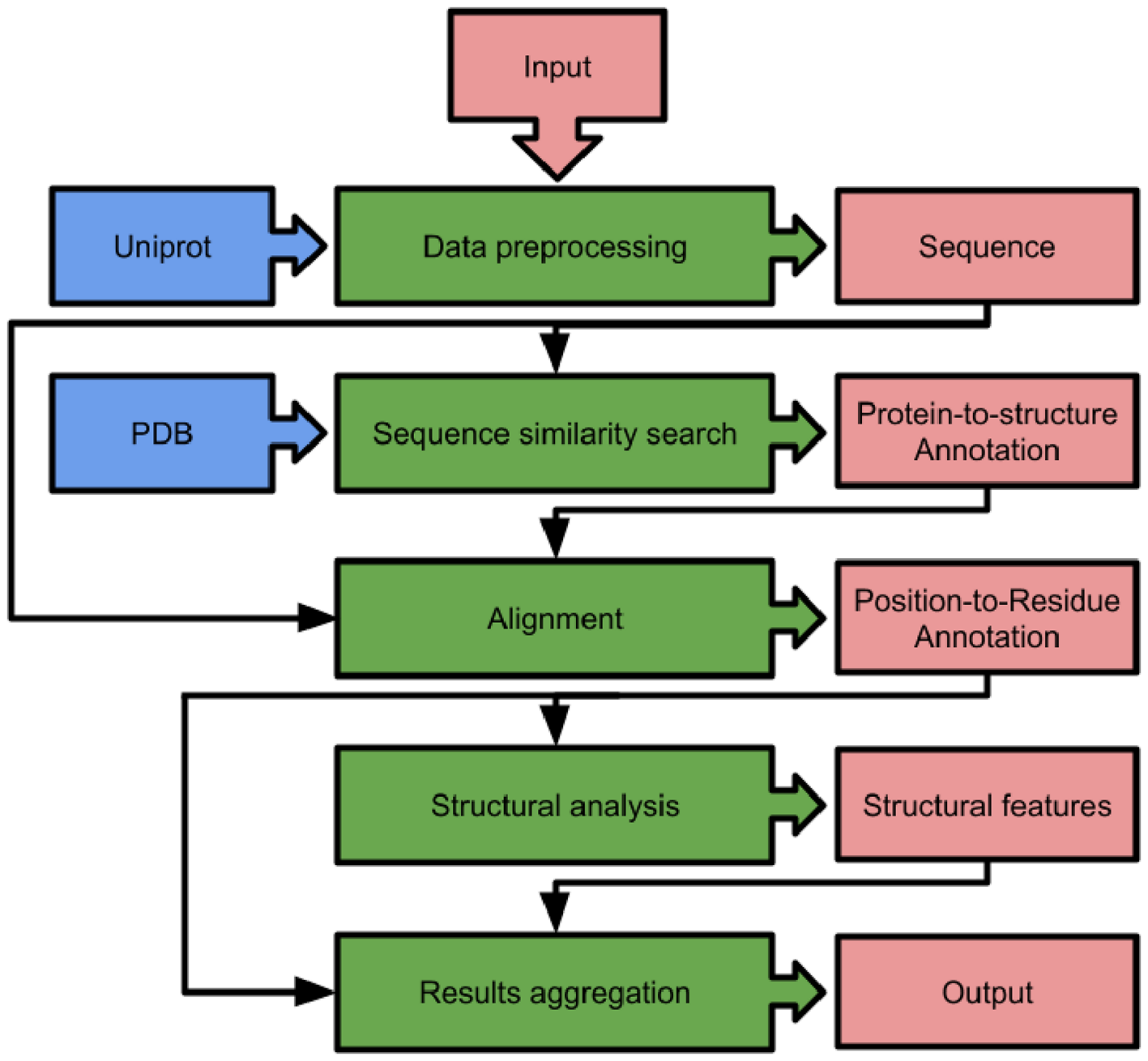
Schematic of computational pipeline of StructMAn. Green boxes are computational sections, red boxes are data structures, and blue boxes are data sources.

StructMAn is a computational pipeline that combines the retrieval and usage of information from publicly available databases with the application of complex computational biology algorithms. The pipeline can be divided into 5 computational steps (Fig. 7).

### Input and preprocessing

Input given to StructMAn can have up to 3 parts. The first part is mandatory and corresponds to protein sequence data. Protein sequences can be submitted via different kinds of protein database identifiers (Uniprot, RefSeq, and HGNC), directly given as fasta-formatted sequences or using PDB entry identifiers. In the latter case, the protein sequence is retrieved by parsing the ATOM records of the PDB file. Due to the “never-compute-anything-twice” policy of StructMAn, it is important to detect protein identifiers that map to the same amino acid sequence. Therefore, the protein identifier mapping services from UniProt are used.

The second part of input can comprise positions or genetic variations in the corresponding protein sequence. It is optional, and if it is not set, all positions in the corresponding sequence will be annotated. Possible genetic variations are single amino acid variations, insertions, and deletions. The third part of the input includes tags, which can be used by users to label specific positions or genetic variations in their input data. These labels will then reappear in the output, and summary statistics for each tag will be calculated to help to use the data for downstream applications. A detailed documentation of the possible input formats for d-StructMAn is provided in the following wiki: https://github.com/kalininalab/d-StructMAn/wiki/Simple-Mutation-List-Format and https://github.com/kalininalab/d-StructMAn/wiki/Fasta-format-file-input.

The given input is split into individual chunks that are processed in a serial manner, while the computations for each chunk are highly parallelized. The chunk size depends on the provided resources. Larger chunks require more memory, while enabling a more effective parallelization. After the chunking, the pipeline starts to loop through the core routine that starts with the sequence retrieval of the input proteins.

### Sequence similarity search and alignment

Each of the protein sequences is put into a sequence similarity search against all protein sequences contained in the PDB database. This step is performed by MMseqs2 [39] by setting the sensitivity parameter and the number of returned sequences to its maximum values. This results in a list of potential structure annotations for each input protein. For each potential sequence-to-structure annotation, a pairwise global sequence alignment is calculated using the Needleman–Wunsch algorithm [40] with a gap opening penalty of 10 and a gap extension penalty of 0.5 without any penalty for the end gaps. The sequence identity is computed ignoring the end gaps and should be above 0.35 to accept the corresponding sequence-to-structure annotation. This cutoff is the more conservative end of the usual 30% to 35% sequence identity threshold used in automated homology modeling pipelines and is based on a study of the relationship between protein sequence identity and their structural similarity [41]. The accepted alignments are used for position-specific structural annotations, where each amino acid from all input proteins is mapped to a list of specific residues in the experimentally resolved structure.

### Annotation using AlphaFold models

In contrast to the annotation to experimentally resolved protein structures, we use 1 AlphaFold [28] model per queried protein sequence for structural annotation. These models do not contain interaction partners and cover the whole range of the given sequence, and thus there is no benefit in using more than 1. Nonetheless, we use MMseqs2 [39] to perform a sequence similarity search in all entries stored in the AlphaFold Protein Structure database [28, 29]. This allows us to use AlphaFold models for proteins, whose corresponding structure is not in the database but bears significant similarity to proteins in the database, as well as for mutant protein sequences or proteins from newly sequenced species.

### Structural analysis of individual structures

Each protein structure or multiprotein complex in a PDB entry that contains at least 1 annotated residue gets completely structurally analyzed, unless processed in lite mode. This means that for each residue in each protein chain contained in the entry, a wide array of structural features are calculated, such as solvent accessibility, interactions to other residues, and molecules present in the structure. The general aim of these features is to specify the functional role of the residue in the structure. Some features describe the location of the residue in the chain, while other features are based on the distances and chemical interactions to other molecules contained in the entry. Some features require a graph-based representation of the corresponding protein complex structures, for which residue interaction networks (RINs) of the corresponding complexes are computed using RINerator [42]. In RINs, each amino acid is represented as a node, and an edge connects 2 nodes if there is a covalent bond or a noncovalent interaction between them. A complete list of calculated features is given in the supplementary materials (Supplementary Table S3).

### Aggregation of annotation results from multiple structures

The same set of features is calculated for each residue in each annotated structure, so that a position that is mapped to residues from multiple structures is assigned a list of feature vectors. Further, we calculate a quality score for each annotation, based on the sequence identity, coverage, and the resolution of the annotated structure. Numerical features can then be aggregated by a weighted mean:

(1) \begin{equation*} W(D) = \frac{\Sigma q_i \dot{d}_i}{\Sigma q_i}, i \in D
\end{equation*}

where *q_i_* is the quality score [27] of a structural annotation *i, d_i_* is the individual numerical value from the analysis of the structural annotation *i*, and *D* is the set of all structural annotations for 1 input.

Since the feature vectors are sparse, the undefined values are not included in the calculation of the weighted sum. This is typical for features that derive from interactions, since the interaction partner might be missing in some of the annotated structures. Here, we aggregate the feature values only from the structures that participate in the interaction. A typical example for aggregating results from multiple structure annotations being advantageous would be multiple structures co-crystallized with different interaction partners. The results aggregation for solvent accessibility values is weighted toward buried residues by multiplying the corresponding quality scores by squared alignment coverage, which introduces a penalty for partially resolved structures. In partially resolved structures, residues might appear to lie on the surface of a protein, because a part of the protein is missing in the experiment; thus, when mapping an amino acid to multiple structures with the result that it is annotated as buried residues as well as as surface, we bias the annotations to buried residues as described above.

### Structural classification

**Figure 8:**
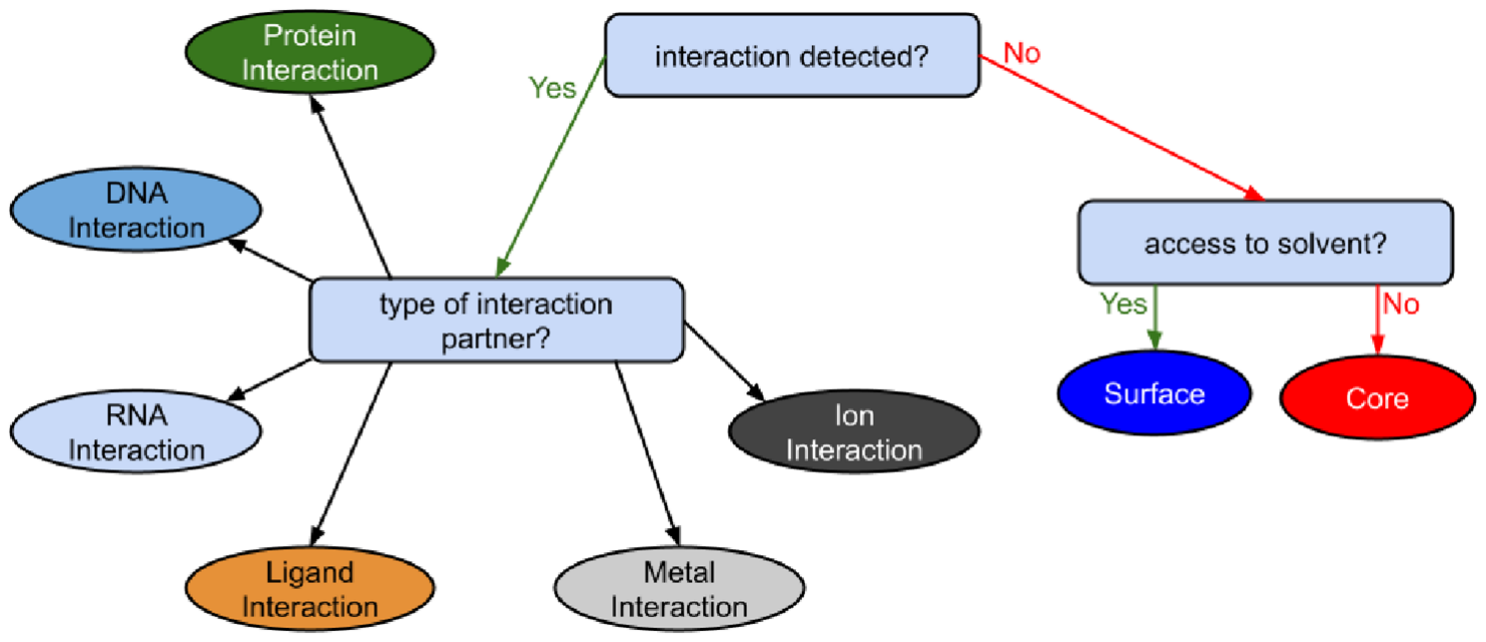
Structural classes are assigned by a decision tree based on the results from the annotation aggregation. The classification aims to describe the functional role of an amino acid residue in the protein structure.

To provide succinct information, StructMAn assigns a structural class to every queried position (see Fig. 8). The idea behind the classification is to give a human-readable interpretation for the functional role of a particular amino acid residue in the protein structure. Therefore, we first determine if the amino acid is part of an interaction interface. If this is the case, the classification is set to the type of the interaction partner: protein, DNA, and so on. Otherwise, the structural class is “core” for residues buried in the protein and “surface” for those with access to the surrounding solvent but not engaged in interactions. This classification is made by considering the aggregated relative solvent accessible area (RSA) of all residues to which the query position is mapped, as described above. The particular threshold for the aggregated values to make the decision reflects the threshold one would use making the decision for a single structure, since the aggregated values occupy the same scale as the individual values. Here we employ a threshold derived by Rost and Sander [43]: RSA >0.16 means “surface” and RSA ≤0.16 means “core.”

### Aggregation of annotation results for indels

**Figure 9:**
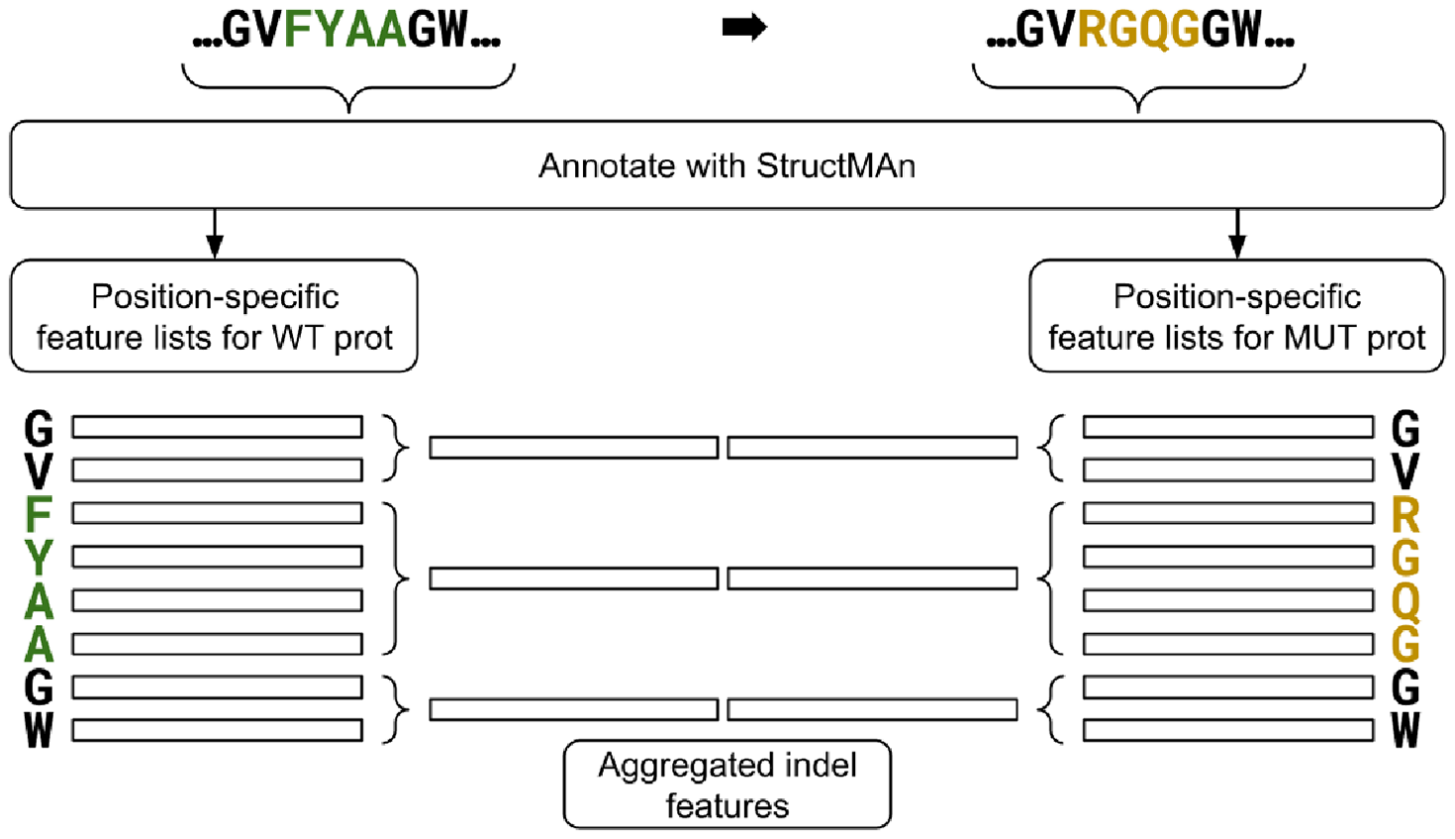
The results aggregation for indels is based on the position-specific results aggregation of the wild-type (WT) protein sequence and the results aggregation of the mutant (MUT) protein sequence. For both protein variants, 3 separate aggregations are performed: left flank of indel, indel region, and right flank of indel. Note that for a insertion, the length for the indel region in the WT is zero, and hence only the flanks produce feature lists (vice versa for deletions and MUT).

For an indel, 2 versions of the query protein are annotated with the d-StructMAn pipeline: the wild-type and the mutant with the indel (see Fig. 9). For both protein versions, we retrieve feature lists for the positions that are part of the indel region and of the 2 flanks (half the length of the indel). These 6 sets of feature lists can then be aggregated again in the same fashion as we aggregate annotation results from multiple structures, and after concatenating them, we receive the feature list for an indel.

### Implementation

d-StructMAn is provided as a Docker image that can be easily pulled from DockerHub. This image can be used in all architectures/operating systems supported by the Docker engine. The image was also tested for use with Podman (on Fedora 35), which allows users to run containers without root privileges. This would allow running this image in high-performance computing clusters/platforms. The use of containerization allows seamless installation of all the dependencies and databases. Folders are bind-mounted into the volumes that were created inside the container to make sure that the data are persistent. The computational pipeline depends on large amounts of data retrieved from Uniprot and PDB. Online retrieval of the data can slow down computations, not only due to transferred data packages but also due to high input/output caused by many writings of temporary files to the disk. Therefore, the installation of d-StructMAn can be expanded. The container includes scripts for downloading a local instance of the PDB and Uniprot, installation of RINerator, and precomputing RINs for all structures in the PDB that will be saved to a local database.

### Module-wise expansion

Installing StructMAn as a container (4 Gb of diskspace) enables all basic functionalities, but in order to reach the highest possible performance StructMAn has to offer, additional modules have to be installed and are described in more details below. The extensions require additional disk space: 100 Gb for PDB, 35 Gb for RINdb, and 50 Gb for Uniprot.

#### MySQL database

The database stores all data produced by the computational pipeline. This has multiple benefits: first, for multiple runs that share the same proteins and/or structures, a lot of computations can be saved. Second, a similar saving is achieved when running large inputs. Since large inputs are chunked down and processed in series by the pipeline, identical computations may appear within the same run. For example, for the annotation of the human proteome, the task was chunked into 115 parts. Overall, over 11 million individual protein-to-structure annotations were processed, while the total number of different PDB entries considered was just around 80,000. Without the database, many of the annotations would have led to a repeated analysis of the same structures again and again. With the database, each structure gets analyzed exactly 1 time. The third benefit is the exportation capability of the database enabling easy shipment of StructMAn results between individual instances of the pipeline. An empty instance of the the database based on the MariaDB engine is installed automatically in the docker container. For the pip version, the user needs to provide a MySQL database server. Then after the configuration of the database credentials, a single command sets up the database structure. This allows the usage of a database server that is physically apart from the system that runs the pipeline, thus enabling more effective usage of provided resources.

#### Local instance of the PDB

Throughout the structural analysis section of the pipeline, many thousands of PDB entries may have to be retrieved. The vanilla version takes that data directly from the RCSB webservices, resulting in a lot of traffic and time delay. Many work groups oriented toward protein structure analysis already maintain an instance of the PDB locally, while other users can use a script attached to d-StructMAn that downloads and configures an instance of the PDB. The same script can be used to update the local file storage and the search index databases that are required for the sequence similarity search section of the pipeline.

#### Local instance of the AlphaFold Protein Structure Database

In addition to the PDB, the AlphaFold Protein Structure Database can be used as a source for protein 3D structure data. d-StructMAn contains a script that creates a local instance of the database retrieving all structures stored at https://ftp.ebi.ac.uk/pub/databases/alphafold/latest/ [44]. The database is unpacked and distributed similarly to the PDB directory scheme to ensure fast local retrieval of individual protein structures. At present, d-StructMAn offers the annotation using AlphaFold structures only when the local instance has been installed.

#### Local instance of a RIN database

An important part of the structural analysis is based on the RIN of the corresponding PDB entry. While a live computation of each RIN during the runtime is possible, users that plan the processing of many or large inputs may consider to precompute the RINs of all PDB entries. We provided a script that creates such a RIN database locally. Similar to the local instance of the PDB, the same script can be used to update the RIN database.

#### Protein sequence and protein identifier mapping database

Different protein or transcript identifiers from different sequence databases can represent an identical protein sequence. Since d-StructMAn operates on the amino acid sequence level, identifying such duplicates at the beginning of the pipeline can save a lot of computations. Uniprot hosts a comprehensive identifier mapping service (https://www.uniprot.org/uploadlists/ [45]) that can also be programmatically accessed. After checking for duplicates, the sequences have to be retrieved. Both tasks require a nonblocked connection to the Internet and produce some traffic, while one also depends on the Uniprot webservices to be reachable. As an optional upgrade, we offer the solution to download all sequences in Uniprot and the identifier mapping tables. They then are locally processed into an SQL database for fast retrieval.

### Scaling solutions

Size and complexity of inputs given to StructMAn can vary a lot. This results in various scaling issues that had to be solved in a way considering the resources provided by the underlying system architecture. We provide several solutions to optimally balance the core load and memory usage.

#### Input chunking

The major mechanism to control memory consumption is the input chunking that divides the input in equally sized subtasks. The size of a chunk is defined by the number of proteins it contains. It increases when more memory is available, while it decreases when more cores are provided. Larger proteins or those that will be mapped to more structures generate more load on the system. Since the number of proteins only roughly estimates the true complexity of a subtask, the input chunking may be very tentative. The implemented parallelization techniques that are explained in the following subsection enable an efficient core usage even when the number of given proteins is lower than the number of available cores.

#### Parallelization of the alignment section

For each protein-to-structure mapping coming out of the initial sequence similarity search, a Needleman–Wunsch alignment without terminal gap penalties with runtime complexity *O*(*nm*), where *n* is the length of the protein sequence and *m* is the length of sequence retrieved from the mapped structure, is computed. Since at this stage of the pipeline, both sequence lengths are known, the necessary runtimes for each task can be estimated quite precisely. We use this to prepare *N* equally complex subtasks, where *N* is the amount of available cores. In this packaging process, we also prefer putting alignment tasks from the same protein together in order to reduce data amounts shared to the resulting subprocesses. In summary, this leads to a packaging strategy that adapts to different types of inputs. An input carrying a single protein will distribute multiple alignments of the same protein to different structures to different subprocesses, and inputs with multiple proteins combine alignments of the same protein together and assign them together to a single subprocess.

#### Parallelization of the structural analysis

The structural analysis is performed separately for different PDB entries. The complexity of most computations is linear by the number of protein chains in the entry, with the exception of analyses that revolve around interaction between different chains, which have a roughly quadratic complexity. While generally we can distribute the analyses of different entries to different subprocesses, this can lead to situations, where the computation time for a large entry in a separate subprocess takes longer than the computation of all entries. To avoid such scenarios, we assign multiple threads to larger structures and subdivide many of the analyses chain-wise. This is a so-called nested parallelization and should be avoided if possible, but in our case, it provided a significant performance increase in practice.

#### Parallelization of the results aggregation

The structural analysis results from many different structures are combined for each position, and this typically requires a lot of resources to look up data from 1 big data structure. Such a task cannot be effectively parallelized in Python without copying the data structure for each subprocess, which leads to an overhead that is larger than the time saved by parallelized processing. The Ray [46] library offers solutions for exactly such problem settings; however, when we increase the number of threads, the overhead also increases until we reach a point where more threads do not result in any performance increase anymore. For large jobs, we implemented an advanced optimization technique that divides the major data structure at a preprocessing step and sends the substructures to individual subprocesses, which then use the usual parallelization, leading again to a nested parallelization setup that can then use a large number of available cores efficiently. Since the preprocessing step also leads to a significant overhead, this technique is best applicable in situations where a large input meets large computation resources.

## Availability of Source Code and Requirements

The code is available on GitHub at https://github.com/kalininalab/d-StructMAn. The implementation is in Python and the code is distributed under the LGPL-2.1 license. Easy installation is provided using containerization software: Docker or Podman.

- Project name: d-StructMAn
- Project home page: https://github.com/kalininalab/d-StructMAn
- Operating system: Linux
- Programming language: Python 3.8
- Other requirements: Docker or Podman
- License: LPGL-2.1
- RRID: SCR_022534
- biotools ID: d-structman

## Availability of Supporting Data and Materials

The full structural annotation of the human proteome and ClinVar mutations is openly available in the *GigaScience* repository, GigaDB, in the *Supporting data for “d-StructMAn: containerized structural annotation on the scale from genetic variants to whole proteomes”* repository [47].

## Additional Files

**Supplementary Table S1**- Human proteome dataset.

**Supplementary Table S2** - ClinVar dataset.

**Supplementary Table S3** - Feature descriptions.

**Supplementary Table S4** - Classification Example.

## Abbreviations

3D: 3-dimensional; DelIns: mutliresidue substitutions; Gb: gigabyte; indels: insertions and deletions; MUT: mutant; PDB: Protein Data Bank; RAM: random access memory; RIN: residue interaction network; RSA: relative solvent accessible area; SAV: single amino acid variation; WT: wild-type.

## Competing Interests

The authors declare no competing interests.

## Funding

The reported study was funded by the joint Russian Foundation for Basic Research (RFBR) and Deutsche Forschungsgemeinschaft (DFG) research project 20-54-12008. A.G. was supported by the German Federal Ministry of Education and Research (BMBF) within the framework of the e:Med research and funding concept (grant SysCARE [01ZX1908A]). S.K. was supported by the IMPRS-CS graduate student fellowship and DFG project number 430158625. S.K.S. was partially supported by the UdS-HIPS-Tandem Interdisciplinary Graduate School for Drug Research. O.V.K. was supported by the Klaus Faber Foundation.

## Authors’ Contributions

A.G. devised the method and implemented the core functionality. S.K.S. and S.K. assisted with the method development and implemented the container. V.R. and O.V.K. conceived the project. All authors wrote the manuscript.

## Acknowledgments

We would like to thank Anne Tolkmitt, Nadezhda Azbukina, Amit Fenn and Olga Tsoy for testing d-StructMAn.

## Supplementary Material

giac086_GIGA-D-22-00032_Original_Submission

giac086_GIGA-D-22-00032_Revision_1

giac086_GIGA-D-22-00032_Revision_2

giac086_Response_to_Reviewer_Comments_Original_Submission

giac086_Response_to_Reviewer_Comments_Revision_1

giac086_Reviewer_1_Report_Original_Submission Roman Laskowski, PhD -- 4/4/2022 Reviewed

giac086_Reviewer_1_Report_Revision_1 Roman Laskowski, PhD -- 7/14/2022 Reviewed

giac086_Reviewer_2_Report_Original_Submission Eduard Porta Pardo -- 4/5/2022 Reviewed

giac086_Reviewer_2_Report_Revision_1 Eduard Porta Pardo -- 8/1/2022 Reviewed

giac086_Supplemental_Files
