# Supplementary material for: d-StructMAn: Containerized structural annotation on the scale from genetic variants to whole proteomes
Source: Gigascience. 2022 Sep 20;11:giac086. doi: 10.1093/gigascience/giac086 (PMC9487898; doi:10.1093/gigascience/giac086)
Supplement: giac086_GIGA-D-22-00032_Revision_1 [file giac086_giga-d-22-00032_revision_1.pdf]

GigaScience

d-StructMAn: containerized structural annotation on the scale from genetic variants to whole proteomes

--Manuscript Draft--

**Manuscript Number:**	GIGA-D-22-00032R1
**Full Title:**	d-StructMAn: containerized structural annotation on the scale from genetic variants to whole proteomes
**Article Type:**	Technical Note
**Funding Information:**	Bundesministerium für Bildung und Forschung (01ZX1908A) — Not applicable
**Abstract:**	Background , Structural annotation of genetic variants in the context of inter-molecular interactions and protein stability can shed light onto mechanisms of disease-related phenotypes. Three-dimensional structures of related proteins in complexes with other proteins, nucleic acids, or ligands enriches such functional interpretation, since inter-molecular interactions are well conserved in evolution. Results , We present d-StructMAn, a novel computational method that enables structural annotation of local genetic variants, such as single-nucleotide variants and in-frame indels, and implements it in a highly efficient and user-friendly tool provided as a Docker container. Using d-StructMAn, we annotated several very large sets of human genetic variants, including all variants from ClinVar and all amino acid positions in the human proteome. We were able to provide annotation for more than 46\% of positions in the human proteome representing over 60\% proteins. Conclusions , d-StructMAn is the first of its kind and highly efficient tool for structural annotation of protein-coding genetic variation in the context of observed and potential inter-molecular interactions. d-StructMAn is readily applicable to proteome-scale datasets and can be instrumental building machine-learning tools for predicting genotype-to-phenotype relationships.
**Corresponding Author:**	Alexander Gress, Ph.D.<br>Helmholtz Institute for Pharmaceutical Research Saarland<br>Saarbrücken, GERMANY
**Corresponding Author Secondary Information:**	
**Corresponding Author's Institution:**	Helmholtz Institute for Pharmaceutical Research Saarland
**Corresponding Author's Secondary Institution:**	
**First Author:**	Alexander Gress
**First Author Secondary Information:**	
**Order of Authors:**	Alexander Gress
	Sanjay Kumar Srikakulam
	Sebastian Keller
	Vasily Ramensky
	Olga Kalinina
**Order of Authors Secondary Information:**	
**Response to Reviewers:**	Reviewer #1: The authors describe a method of annotating single-nucleotide variants and indels with information from 3D protein structures. It is an improved version of their previous tool StructMAn and can be installed and run locally as a command line tool.<br><br>The paper describes in detail how the tool operates and how it can be installed. Indeed, it rather focuses on the technical aspects and various statistics such as the numbers of variants, proteins, and performance issues. What it fails to provide are examples of the annotations themselves, how they are presented, and how these

Powered by Editorial Manager® and ProduXion Manager® from Aries Systems Corporation

might be useful. A list of features given in Table S3, but at least one example of the output would make it clearer what one gets from the system. It does, after all, require a significant investment of effort to install and get running, so potential users need to be able to see what they will be getting out of it at the end of the process.

Response:

We would like to thank the reviewer for their patience with the installation process. The overall installation of the whole pipeline is quite complex due to the usage of many dependencies, online resources and especially the integrated MySQL database. For the publication in GigaScience our focus was on the processing of very large inputs, and to achieve that the pipeline needs all the previously mentioned parts installed. While we plan to provide a full installation guide outside of the container in the future, at present containerization was an ideal solution to provide the full potential of the pipeline without the need of a trained systems administrator to install the tool. In the future, we also want to provide a quicker installation that limits the pipeline to the 'lite-mode'. This installation will then not be recommended to process large inputs, but can be very easily installed and used. To showcase the output capabilities of the tool, we added a section explaining the results for one example with a single amino acid substitution D833A in the histone-lysine N-methyltransferase EHMT2.

Reviewer #2: Comments

The authors describe the d-StructMAn containerized methodology and provide a comprehensive summary of its performance, with a nice reading flow, especially in the introduction. They present the performance of the tool by structurally annotating the human proteome as well as a clinically relevant database such as ClinVar. Additionally, d-StructMAn authors provide insights on important aspects that influence their performances, which is of general interest and value to the community. Regarding the implementation of their tool, they are very aware of the data load the user will have to deal with and provide nice adaptations of their software. Other important aspects of the method, in particular input formats, therholds selection and the lack of high quality structural models from AlphaFold or RosettaFold are less detailed and would benefit of some clarification.

Response:

We are very happy to receive a reviewer that has dealt with a similar topic in the past and can delve even into the finest details of this project. We support multiple types of input formats, and explaining them in details would take too much space, thus we chose to provide the full documentation in the wiki that comes with the git repository (https://github.com/kalininalab/d-StructMAn/wiki/Simple-Mutation-List-Format). We will continue to improve the content of the wiki in the future.
We tried to give better reasonings for our selected thresholds (see point-to-point responses).
We added the support for AlphaFold models, which needed quite some time to implement and test, which is the main cause for the long amount of time we needed to submit the revised manuscript.

Major

1) The choice of using all isoforms instead of a single isoform per gene can potentially be problematic. On one hand, it is an unusual experiment and the results, therefore, are interesting. On the other hand, this biases significantly the "overall" results towards genes with more isoforms. Another issue is that this choice makes it hard to interpret the rest of the results of the paper. For example, as it is written, I cannot know whether in Table 2 the "10 proteins, very many hits" include 10 proteins from 10 different genes, 10 protein isoforms from the same gene, or any combination in between. I'd suggest that the authors also show the results of the coverage with one isoform per gene. This will also make their results easier to compare to the existing literature.

Response:

All proteins in the benchmark datasets are not isoforms of one or several genes, but

Powered by Editorial Manager® and ProduXion Manager® from Aries Systems Corporation

rather come from different genes. We use different isoforms only in the whole human proteome experiment, but the user is free to provide one or several isoforms per gene as input. We tried to make it clearer in the text that “using different isoforms” is a matter of input, not hard-coded in the pipeline.
Analyzing one isoform per gene in the human proteome does not change the results much, since the majority of proteins in this dataset only have one isoform (78.24%), so we chose to focus on the isoform-specific analysis, since this is the unique feature of our pipeline to differentiate between different isoforms in the input Uniprot identifiers..

2) Regarding the sequence alignment: The authors do not mention why their minimum sequence homology filter is set to 35%. This is a very important choice that would alter results significantly so I believe this should be clarified in the manuscript.

Response:

We use the threshold of 35 % as a more conservative version of a commonly accepted threshold of 30-35 % that is considered sufficient sequence identity for homology-based modelling. This threshold is based on the analysis of the relationship between sequence identity and structural similarity by Burkhard Rost (doi:10.1093/protein/12.2.85).

3) Do the authors filter the sequence alignment also by evalue? If one only filters by sequence identity it is possible to retrieve many non-significant hits for short sequences. This could be an even more significant problem if including multiple short isoforms for some genes.

Response:

We do not apply filtering by e-value, since the notion of significant and non-significant hits is somewhat different here from a typical sequence search. The key principle of the method is to transfer functional conclusions from structures of related proteins, therefore some degree of redundancy is intentional. Of course, for a short isoform, we will also annotate the structures of their longer variants, since they will be returned by the sequence similarity search and aligned to the identical segments. This is intentional, since the part of the structure that the short isoform is mapped to will be structurally analyzed and the key principle of the method is to transfer functional conclusions from structures of related proteins. We have to assume here that the function of the short isoform does not change significantly, when expressed without the other domains.

4) Does it make sense to aggregate the features from multiple structures? For example, if a residue is exposed in one structure but buried in a different one due to a conformational change, the average of both (buried and exposed) is difficult to interpret from a biological perspective.

Response:

We believe that aggregating results from different structures is important and provides better biological insights. Considering every structure separately quickly becomes infeasible for well studied proteins. However, the point raised by the reviewer is a valid one and constitutes a challenge. We approached this by favoring buried/core residues in the aggregation step. There are two reasons for this approach: 1. In partially resolved structures, buried residues can appear on the surface, while in reality being buried, thus we trust an annotation as a buried residue more than as a surface residue. We take the alignment coverage into account to a greater extent when aggregating surface exposure features compared to the aggregation of other structural features. 2. Buried and core residues tend to be more important for the stability and function of the protein compared to surface residues, and the same we assume to be true for residues that are surface residues in one conformation and buried in another conformation. Thus, annotating those switching residues as buried is more useful. We added more details in text to clarify our decisions. A potential future improvement would be to detect such cases and introduce another exposure type in our classification scheme. However, we would like to refrain from commenting on mutations’ functional importance in this manuscript, since it only meant to introduce the classification tool.

Powered by Editorial Manager® and ProduXion Manager® from Aries Systems Corporation

	5) Following the same thought as in point 3, how does the decision tree for the "Structural classification" decide which feature to assign when there is more than one interaction feature (i.e. a residue that interacts with a protein in a structure and with a nucleic acid in another)?<br><br>Response:<br><br>There are two classification schemes that are used in the results of the tool. First, a more complex one, lists all different interactions as multiple interactions. For the simpler scheme, we have to choose one of the types, here we take the interaction type with the highest aggregated interaction score.<br><br>Or if a residue is exposed in one structure and accessible in another?<br><br>Response:<br><br>Please see our response to point 4 that clarifies the assignment based on surface exposure.<br><br>6) Related to the previous point, the authors do not specify the RSA threshold they use to determine if a residue is buried or not. Again, I think this would affect the structural functional annotation, and therefore the reader should be aware of.<br><br>Response:<br><br>We added the threshold and explanation of how we chose it into the text.<br><br>7) The authors do not clarify how the input of protein positions or variants is. For example, variants do not follow a standard format so I think it is of importance to specify this to the reader.<br><br>Response:<br><br>We thought the details of our input formats would be too technical for the manuscript (which is already very technical) and left the documentation for it in our wiki. While there is sadly no standard format, we tried to design our format to comply with other formats previously used in the field.<br><br>8) In section "Clinically relevant genetic variations", it is unclear what are the 123 features and the 600 for either SNVs and indels. In supplementary table S3 only includes 75 d-StructMAn features, so I guess the rest of features are ClinVar features.<br><br>Response:<br><br>We added a remark in the section "Clinically relevant genetic variations" where to find more details on the feature generation. In supplementary table S3, some feature descriptors contain brackets “[“ and “]” denoting a list of alternative words that each describe a different feature. Thus, the total number of features (123) is higher than the number of rows (74) in table S3. For indels, most features come in six different variants (WT, MUT, left flank WT, left flank MUT, right flank WT, right flank MUT), which results in a total 600 features.
**Additional Information:**	
**Question**	**Response**
Are you submitting this manuscript to a special series or article collection?	No
**Experimental design and statistics**	Yes

Powered by Editorial Manager® and ProduXion Manager® from Aries Systems Corporation

Full details of the experimental design and statistical methods used should be given in the Methods section, as detailed in our Minimum Standards Reporting Checklist. Information essential to interpreting the data presented should be made available in the figure legends.<br><br>Have you included all the information requested in your manuscript?	
**Resources**<br><br>A description of all resources used, including antibodies, cell lines, animals and software tools, with enough information to allow them to be uniquely identified, should be included in the Methods section. Authors are strongly encouraged to cite Research Resource Identifiers (RRIDs) for antibodies, model organisms and tools, where possible.<br><br>Have you included the information requested as detailed in our Minimum Standards Reporting Checklist?	Yes
**Availability of data and materials**<br><br>All datasets and code on which the conclusions of the paper rely must be either included in your submission or deposited in publicly available repositories (where available and ethically appropriate), referencing such data using a unique identifier in the references and in the "Availability of Data and Materials" section of your manuscript.<br><br>Have you have met the above requirement as detailed in our Minimum Standards Reporting Checklist?	Yes

LaTeX - Main Document (TeX file) Click here to access/download;LaTeX - Main Document (TeX file);main.tex

GigaScience, 2017, 1–11

doi: xx.xxxx/xxxx
Manuscript in Preparation
Paper

PAPER

d-StructMAn: containerized structural annotation on the scale from genetic variants to whole proteomes

Alexander Gress[1,2,*], Sanjay K. Srikakulam[1,2,3], Sebastian Keller[1,2,4], Vasily Ramensky[5,6] and Olga V. Kalinina[1,7,8]

[1]Helmholtz Institute for Pharmaceutical Research Saarland (HIPS) / Helmholtz Centre for Infection Research (HZI), Saarbrücken, Germany and [2]Graduate School of Computer Science, University of Saarland, Saarbrücken, Germany and [3]Interdisciplinary Graduate School of Natural Product Research, Saarland University, Saarbrücken, Germany and [4]Research Group Computational Biology, Max Planck Institute for Informatics, Saarbrücken, Germany and [5]National Medical Research Center for Therapy and Preventive Medicine of the Ministry of Healthcare of Russian Federation, Moscow, Russia and [6]Faculty of Bioengineering and Bioinformatics, Lomonosov Moscow State University, Moscow, Russia and [7]Medical Faculty, Saarland University, Homburg, Germany and [8]Center for Bioinformatics, Saarland Informatics Campus, Saarbrücken, Germany

*alexander.gress@helmholtz-hips.de

Abstract

Background, Structural annotation of genetic variants in the context of inter-molecular interactions and protein stability can shed light onto mechanisms of disease-related phenotypes. Three-dimensional structures of related proteins in complexes with other proteins, nucleic acids, or ligands enriches such functional interpretation, since inter-molecular interactions are well conserved in evolution. **Results**, We present d-StructMAn, a novel computational method that enables structural annotation of local genetic variants, such as single-nucleotide variants and in-frame indels, and implements it in a highly efficient and user-friendly tool provided as a Docker container. Using d-StructMAn, we annotated several very large sets of human genetic variants, including all variants from ClinVar and all amino acid positions in the human proteome. We were able to provide annotation for more than 46% of positions in the human proteome representing over 60% proteins. **Conclusions**, d-StructMAn is the first of its kind and highly efficient tool for structural annotation of protein-coding genetic variation in the context of observed and potential inter-molecular interactions. d-StructMAn is readily applicable to proteome-scale datasets and can be instrumental building machine-learning tools for predicting genotype-to-phenotype relationships.

Key words: Single-nucleotide variants, indels, genetic variation, protein structure, protein interactions, structural annotation, Docker container

Introduction

Background

In the age of next-generation sequencing, large-scale genetic diversity within populations became apparent. A single human

Compiled on: July 6, 2022.
Draft manuscript prepared by the author.

Key Points

- A novel bioinformatics tool for structural characterization of genetic variants is presented
- Single-nucleotide variants and indels are described with respect to inter-molecular interactions in homologous protein complexes
- An efficient implementation using Docker container allows for analysis of large whole proteome-scale datasets

individual of European ancestry carries around three million genetic variants [1], of which up to 14,000 occur in coding regions and lead to an amino acid substitution [2]. Additionally, up to 3,000 short insertions or deletions (indels) occur in the coding regions and either lead to a frame shift or cause an indel in the corresponding protein sequence [3]. These coding variants that result in non-synonmous substitutions or indels in the protein sequence are the focus of this study. Additional sources of indels are alternatively spliced isoforms that result in retaining or skipping exons or parts of introns in the translated sequence. Alternative splicing is wide-spread across eukaryotes and human tissues [4]. UniProt lists an average of approximately four isoforms per a protein-coding gene in its reference human proteome.

Although most of sequence variants have no functional or pathogenic effect, in some cases even a single mutation can be disease-causing [5, 6]. Experimental characterization of all variants is infeasible; since most of them are rare and occur in only one or few individuals [7], statistical characterization of their association with diseases seems impossible, too. This emphasizes the importance of computational tools for predicting functional and/or pathogenic effects of sequence variants. Many such tools were developed specifically for non-synonymous (missense) variants and take into account the protein three-dimensional (3D) structure context of an input variant. One of the keystones in this field is PolyPhen [8, 9] which combines a number of features related to protein sequence and 3D structure in a Bayes classifier that predicts an individual variant to be deleterious or benign. Other methods also rely on conservation of the mutated position in a protein alignment [10, 11], or use additional features derived from phylogenetic analysis or analysis of the protein 3D structure [12, 13, 14, 15, 16]. Meta-methods combine outputs from several prediction tools [17, 18, 19].

Of those tools that employ protein 3D structure to derive various predictive features, several put variants of interest in the context of protein-protein interactions [20], and interactions with other biologically relevant molecules [21]. Indeed, features related to protein three-dimensional structure were proven to be instrumental for predicting pathogenic effects of mutations [22]. Various intricate facets of structural analysis have also been implemented in databases and computational tools [23, 24, 25, 26] with one common drawback: they rely on information from individual structures and do not integrate the multiple relevant findings.

As far as structural annotation of larger variants is concerned, the majority of methods are developed to annotate single amino acid replacements, and, to the best of our knowledge, there is currently no structural annotation pipeline that is able to map indel-type genetic variants to protein 3D structures.

High-performance structural annotation

In this study, we present d-StructMAn, a new improved implementation of our earlier tool StructMAn[27], shipped in a convenient and easily installable container form and extended to annotation of short in-frame indels and alterations arising as a consequence of alternative splicing events. d-StructMAn produces a wide range of structural features by combining information from experimentally resolved structures of many related proteins, which is a unique feature of the StructMAn family tools. In addition to experimentally resolved protein 3D structures, d-StructMAn can also harness information from all protein structure models stored in the AlphaFold Protein Structure Database [28, 29]. To the best of our knowledge, this is the first tool with this property that can also analyze genomic indels and consequences of alternative splicing events. Additionally, we provide structural annotation of all proteins in the human proteome (all canonical protein sequences and all isoform sequences listed in UniProt) and all pathogenic and benign genetic variants from ClinVar[6] as a publicly available data resource.

Data Description

Human proteome dataset

The human proteome dataset contains 101,014 protein isoform sequences belonging to 79,038 human protein entries in UniProt [30]. We generated the dataset by downloading (on the 5th December 2021) all sequences from https://www.uniprot.org/uniprot/?query=human&fil=proteome%3AUP000005640+AND+organism%3A%22Homo+sapiens+%28Human%29+%5B9606%5D%22&sort=score# [31] choosing for format: "FASTA (canonical & isoforms)". StructMAn can process this FASTA file directly. However, to test StructMAn's functionality to retrieve sequences in an isoform-specific manner, we have also extracted accession identifiers and used them as an input. The resulting file (Supplementary file S1) represents protein sequence data over 4 million individual amino acids and was used as input file for StructMAn.

ClinVar

The freely accessible ClinVar [6] database contains human mutations annotated with clinical outcomes. We downloaded (on 13th September 2021) the "variant_summary.txt.gz" from https://ftp.ncbi.nlm.nih.gov/pub/clinvar/tab_delimited/ [32]. We retained DelIns (mutli-residue substitutions), insertions or deletions with "Assembly" field equal to GRCh38 and a RefSeq[33] protein identifier provided. The provided "clinical significance" field was simplified to Pathogenic, Benign and Unknown. The resulting dataset is described in the Table 1 and Supplementary file S2.

Analyses

Annotation of the human proteome

The structural annotation of more than 100,000 protein sequences represents size-wise the ultimate challenge for a structural annotation method, but on the other hand reviews

Table 1. The ClinVar dataset.

Mutation type	Pathogenic	Benign	None	Total
SAV*	41557	48285	317261	407103
Deletion	1615	775	5389	7779
Insertion	108	82	372	562
DelIns**	287	46	750	1083
All	43567	49188	323772	416527

*Single amino acid variation.
**Multi-residue substitutions

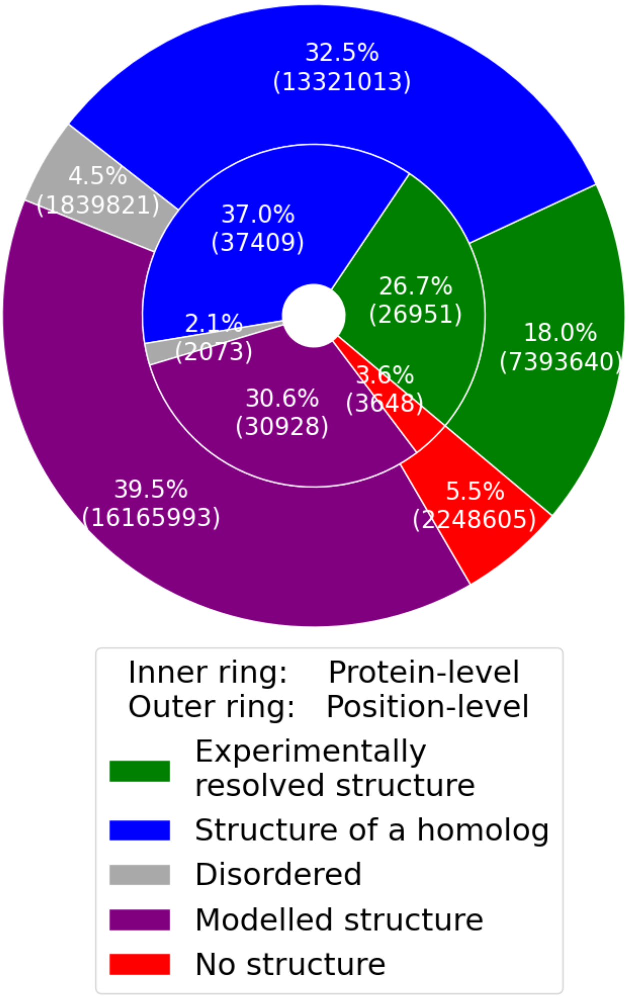

Figure 1. Proportion of proteins and positions from the human proteome dataset that could be mapped to structure data. Experimentally resolved structure (green) denotes that the protein (position) was mapped to at least one structure with sequence identity $\geq$ 0.99, structure of a homolog (blue) denotes that the protein (position) was mapped to at least one structures with sequence identity in the range from 0.35 to 0.99. Modelled structure (purple) denotes that the protein (position) could only be mapped into modelled structure that is not directly supported by experimental data. Disordered (grey) denotes proteins and positions that could not be mapped to any structure, but are predicted by IUpred3 [34] to be disordered (for proteins all positions have to be predicted to be disordered). No structure (red) denotes all other proteins and positions.

the future applicability of the method by estimating what fraction of the human proteome can be mapped to the structure data.

For only 21.7% of all proteins the corresponding experimentally resolved structure is available in the PDB (Figure 1) and since not all of them were resolved in full length this estimate is reduced to 13.9% of the positions. Mapping proteins

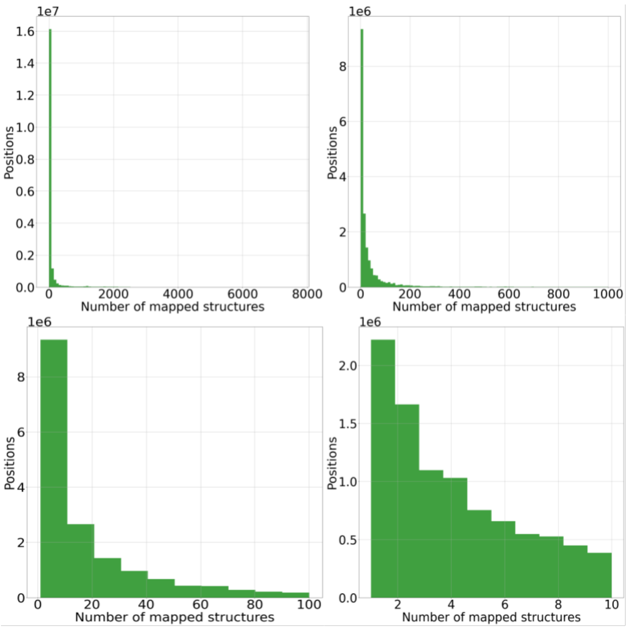

Figure 2. Distribution of the number of structures that could be mapped to a position in the annotation of the human proteome dataset. Each subplot shows the same distribution with a different zoom.

to structures of homologs drastically increases the usability of structural annotation to 60.5% of all proteins in the human proteome.

When a position can be structurally annotated, it can rarely be mapped to only structure. Most of the times, it is possible to identify more than one structure for a position that can be used for structural annotation (Figure 2). Hence, another challenge of structural annotation is to manage the multiple sources of structural information. In such cases, StructMAn provides the user with recommendations listing the structure with the highest sequence identity and a structure considered to be the most representative biologically. The latter recommendation is based on the the structural analysis of all annotated structures and aggregation of relevant information. Sometimes both structures (maximal identity and recommended) may be the same one.

Another advantage of annotation using all available structures is for proteins with partially resolved structures that cover different regions of the protein. This way, StructMAn could annotate 15% more (46% vs. 31%) positions compared to a strategy, where only the structure with maximum sequence identity is chosen (Figure 3).

Clinically relevant genetic variations

ClinVar contains genetic variations labeled with their clinical outcome and is routinely used in numerous supervised machine learning methods aimed to predict the effects of genetic variations [35, 22, 36, 37]. This makes ClinVar the ideal testing ground to showcase the feature generation capabilities of StructMAn. It also demonstrates the new indel annotation and analyses that generated features specific to indel-type genetic variations.

We performed structural classification of all positions in the human proteome and variant positions in the ClinVar dataset (more details in the Methods section) and compared structural classes distribution depending on the ClinVar variant clinical significance (Figure 4). Structural classifications for benign genetic variants, both SAVs and indels, are distributed similarly to the classifications for all amino acids in the human

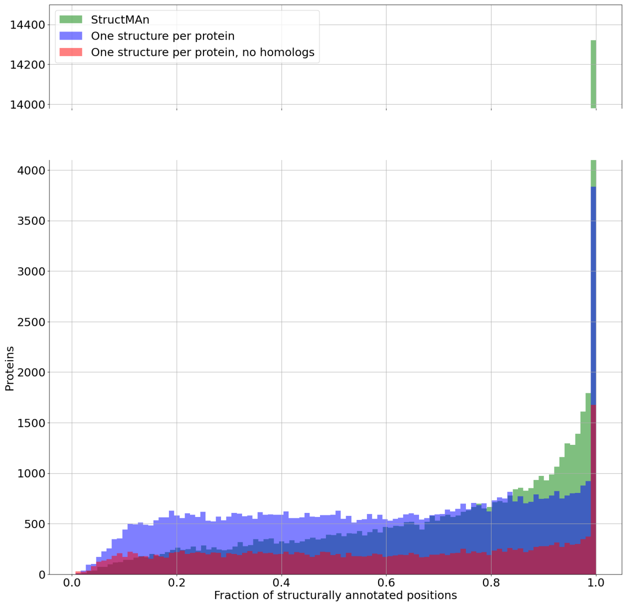

Figure 3. Distribution of the proportion of positions per protein that could be mapped to a structure in the annotation of the human proteome dataset. Green: fraction of positions annotated using multiple structures found by StructMAn; blue: fraction of positions annotated using only one structure per protein (highest sequence similarity was used, in case of same sequence similarity, higher alignment coverage was preferred); red: fraction of positions annotate without considering structures of homologs.

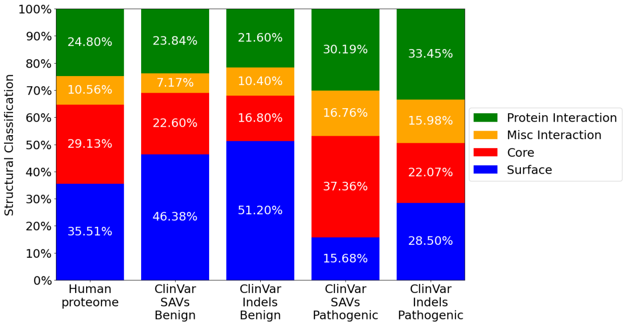

Figure 4. Each stacked barplot denotes the distribution of structural classifications for a dataset. Only positions that could be mapped to at least one structure are considered for this figure. Protein interaction: amino acids that are part of a protein-protein interaction interface; Misc interaction: amino acids that are part of an interaction with a non-protein partner, DNA for example; Core: amino acids in the core of the protein; Surface: amino acids classified to have access to solvent (and not involved in interactions).

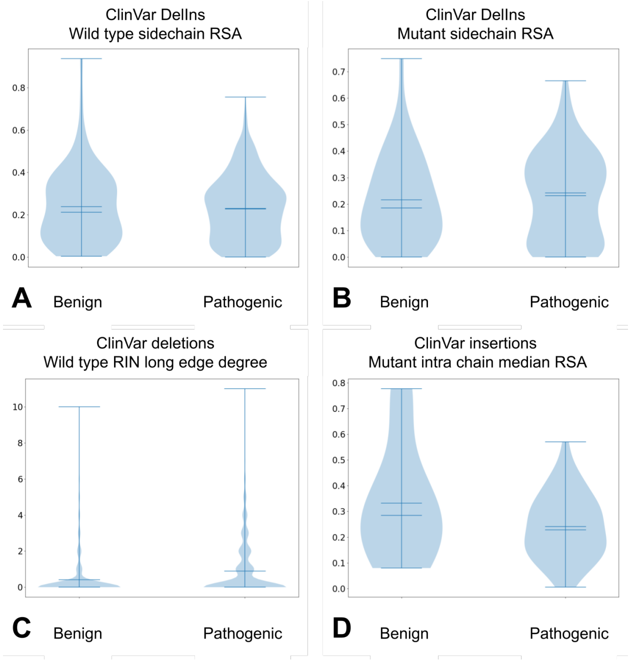

Figure 5. Violin plots for four example features. Left and right plots display the distribution of feature values for benign and pathogenic variants in ClinVar, respectively. **A:** relative surface area (RSA) value for chain atoms in the structures used for annotation of the wild-type protein. Only DelIns (mutli-residue substitutions). **B:** Same as A, but for the structures used for annotation of the mutant protein. **C:** Only deletions. The number of spatial interactions to other amino acids in the same polypeptide chain and separated by more than 6 residues in the sequence. **D:** Only insertions. Median solvent access of residues from other proteins (co-crystallized structures) that lie in a 10Å sphere around the annotated residue.

proteome. Pathogenic SAVs have an increased tendency to be located in the protein core. This is also the case for pathogenic indels, but not as strongly as for pathogenic SAVs. Further, pathogenic SAVs and indels are enriched on interaction interfaces. These results are in agreement with our earlier analysis [38], e.g. benign variants are enriched for non-interacting surfaces, whereas pathogenic and disease-associated variants are depleted in these regions, but tend to appear more often on interaction interfaces.

Most missense variant effect prediction methods based on supervised machine learning models rely on ClinVar as the training and testing dataset. We used d-StructMAn to generate 123 features for every SAV and 600 features for every indel in ClinVar (more details about feature generation are provided in the Methods section: for SAVs, see section 'Structural analysis of individual structures', for indels, see section 'Aggregation of annotation results for indels'). Structural features generated for SAVs are based on the results of the structural analysis of the residues in the wild-type, while for indels the wild-type and mutant versions of the protein are analysed separately and used for features generation (see Methods for more details). We selected four example features and plotted their value distributions for benign and pathogenic indels (Figure 5). This gives an example of d-StructMAn employed for the preliminary feature analysis and selection, since a machine learning method would benefit from features that discriminate the datasets of interest.

Performance benchmark

In order to benchmark the runtime performance of StructMAn on different computing systems and different configurations, we generated six datasets. Three of them contain 10, 50 or 100 proteins that all can be mapped to only one or very few PDB entries. Two other datasets contained 10 or 50 proteins that each could be mapped to around 100 PDB entries. The last dataset contains 10 proteins that are very challenging for the pipeline, since they can be mapped to over 13,000 PDB entries, since this dataset includes such proteins as kinases and antibodies. The total number of mapped structures and hence the total number of structurally analysed residues is the main cause for computational complexity. This number does not directly depend on the number of proteins in the dataset (Table 2).

We processed the benchmark datasets on different systems and different configurations. First, these configurations differ in whether we used local resources (local instances of Uniprot and PDB, see Methods for more information). Second, they differ in whether we used the so-called lite mode that switches off the usage of the internal StructMAn database and performs

Table 2. The benchmark datasets. The first three datasets contain proteins that can be mapped to very few structures. The third and fourth datasets contain proteins that could be map to many structures (around 100). The last dataset contains 10 proteins that can be mapped to >13,000 of structures in total.

Dataset	Positions	Mapped PDB entries	Analyzed residues
10 proteins, few hits	1,398	14	7,242
50 proteins, few hits	13,612	70	45,014
100 proteins, few hits	33,789	146	129,465
10 proteins, many hits	2,415	1,216	678,472
50 proteins, many hits	29,726	5,339	2,709,267
10 proteins, very many hits	1,440	13,109	9,753,815

Table 3. Condensed classification output for position D833 in A2ABF8.

Weighted mainchain location	Core
Weighted sidechain location	Surface
RIN class	Multiple interactions: sidechain contact with a ligand and sidechain contact with a protein
Amount of structures this position is mapped to	291
Recommended structure	2V4H:C 118:D

all the calculations and retrievals on the fly. The lite mode still can use local resources, but does not store intermediate results, thus it is faster on smaller inputs, but slower on larger inputs and when run multiple times in succession. All benchmark runs are conducted on an empty StructMAn database instance, thus we only measure the overhead of filling the database and not the amortized benefits one would receive from successive usage of StructMAn, when many intermediate results can be reused. Therefore, lite mode runs always have an advantage over the default mode runs in this benchmark. Runtime was measured in two systems and four different configurations for each dataset (Figure 6).

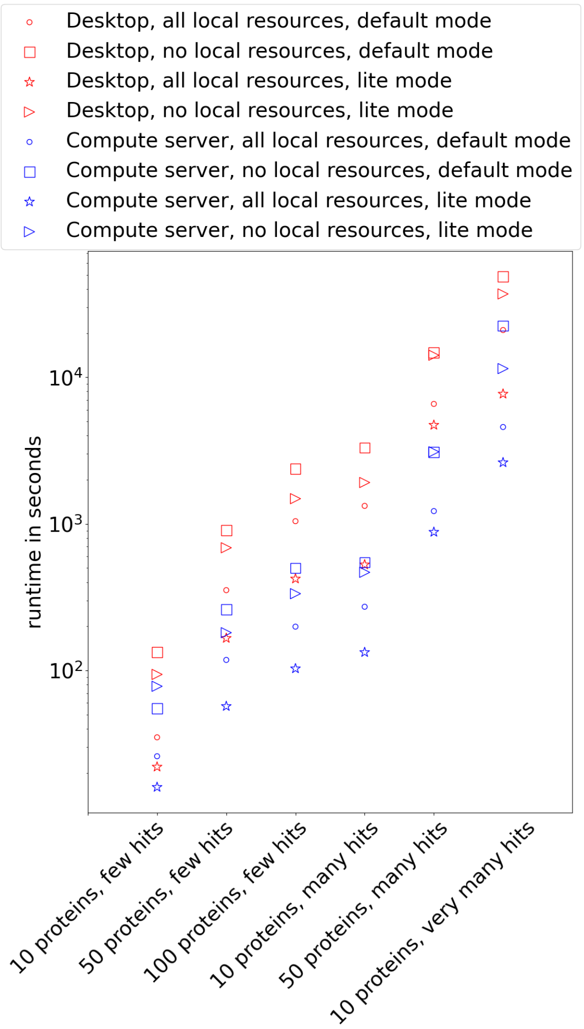

Figure 6. Scatter plot showing runtime performance of StructMAn using different systems and different configurations. Red markers denote a normal desktop computer and blue markers denote a high-performance computing server. Different marker shapes denote different configurations of d-StructMAn.

Output example

d-StructMAn is able to produce very comprehensive outputs, and here we describe an example annotating one SAV D833A in the histone-lysine N-methyltransferase EHMT2 (Uniprot accession: A2ABF8).

Classification table

The classification table presents the integral output of the pipeline and is provided in a tab-separated (.tsv) file format. This table contains 28 values per queried position. The most informative of these values are summarized in Table 3 (we provide the full list of values in Supplementary Table S4).

From the selected values one can see that the sidechain of the queried amino acid is part of the protein surface, while its mainchain belongs to the core of the protein. Further it participates in interactions with low-molecular-weight ligands and other proteins. We can see that the example position was mapped to 291 different experimentally resolved protein 3D structures and one of them was provided as recommended structure (including the corresponding chain identify and residue number).

Feature table

The feature table is also a tab-separated (.tsv) file containing one row per queried position. It contains all values computed during the structural analysis that could be used as features in a machine learning method. A manual interpretation of the feature table is much harder, thus we focus on three specific feature values (Table 4) to get a deeper insight into the interactions the queried amino acid position engages in. The shown scores reflect the strength of interaction between mainchain atoms or sidechain atoms of the mutated position and different types of interaction partners. The given interaction score between sidechain atoms and low-molecular-weight ligands (0.00707) is relatively low and indicates that the protein-protein interaction is perhaps the more relevant type of interaction in this case. When comparing the protein interaction scores between sidechain atoms (0.02437) and mainchain atoms (0.01546), we can see that the interaction is mitigated more by the sidechain part of the amino acid, which agrees well with the observation that we made regarding surface/core orientation based on the classification table. The overall distribution of interaction scores over all considered proteins (data not shown) indicates that these scores are comparably low, thus while the amino acid clearly participates in the interactions, it might not be the most important player for it.

Table 4. Three examples of feature table entries of protein A2ABF8 D833A.

Sidechain ligand score	0.00707
Sidechain protein score	0.02437
Mainchain protein score	0.01546

Discussion

In this study, we presented the structural annotation method d-StructMAn. To our knowledge, it is the first fully automated structural annotation method that can be installed locally and run as a command line tool inside a Docker container. We annotated two big datasets with d-StructMAn: all proteins in human and ClinVar (the annotations are publicly available, see Availability of source code and requirements section). The annotation of the human proteome showed that in practice structural annotation is possible for more than 50% positions, but only if the structural annotation method considers structures of homologs. A great advantage of d-StructMAn is the analysis of all available homologous protein structures in addition to aggregation of the results. These annotations will be useful for scientists in many practical application scenarios.

The performance benchmark confirmed that d-StructMAn runs well on personal laptop and desktop computers. However, for the annotation of large-scale datasets, we would still suggest using a compute server. For instance, the annotation of the human proteome took over 55 hours on our server consuming 250 cores and 1511Gb RAM. The same system processed the most difficult test dataset in the benchmark section in 1.5 hour, while the desktop system (11 cores, 16Gb RAM) took almost 6 hours.

Potential implications

The feature vectors generated by d-StructMAn structural analysis for each given protein position are ideal to be fed into complex supervised machine learning methods. The history of the application of protein structure-based features in mutation effect prediction is surprisingly vacant. This is due to two major challenges: First, the computational work and the implementation needed to generate structural features can not be underestimated. This hurdle should now be solved by our containerized structural annotation method. The second challenge is the sparsity of structural features, since they are not available for all positions in all proteins. Here, we implemented a variety of techniques to increase the coverage by considering structures of all homologous proteins and aggregating results from them. The gradual growth of the Protein Data Bank size and the recent developments in the protein structure prediction [28] also help to overcome the problem of incomplete protein sequence space coverage.

Methods

StructMAn is a computational pipeline that combines the retrieval and usage of information from publicly available databases with the application of complex computational biology algorithms. The pipeline can be divided into five computational steps (Figure 7).

Input and preprocessing

Input given to StructMAn can have up to three parts. The first part is mandatory and corresponds to protein sequence data. Protein sequences can be submitted via different kind of protein database identifier (Uniprot, RefSeq, and HGNC), directly given as fasta-formatted sequences, or using PDB entry identifiers. In the latter case the protein sequence is retrieved by parsing the ATOM records of the PDB file. Due to the 'never-compute-anything-twice' policy of StructMAn, it is important to detect protein identifiers that map to the same amino acid sequence. Therefore, the protein identifier mapping services from UniProt are used.

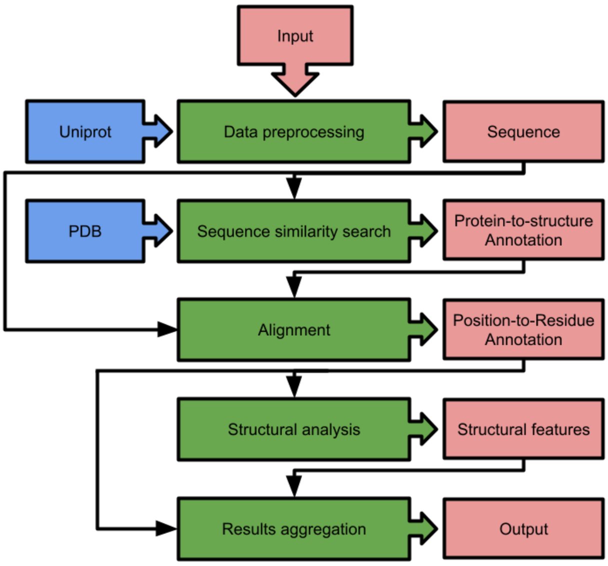

Figure 7. Schematic of computational pipeline of StructMAn. Green boxes are computational sections, red boxes are data structures, and blue boxes are data sources.

The second part of input can be comprised of positions or genetic variations in the corresponding protein sequence. It is optional, and if it is not set, all positions in the corresponding sequence will be annotated. Possible genetic variations are single amino acid variations, insertions and deletions. The third part of the input are tags, which can be used by users to label specific positions or genetic variations in their input data. These labels will then reappear in the output, and summary statistics for each tag will be calculated to help to use the data for downstream applications. A detailed documentation of the possible input formats for d-StructMAn is provided in the wiki: https://github.com/kalininalab/d-StructMAn/wiki/Simple-Mutation-List-Format and https://github.com/kalininalab/d-StructMAn/wiki/Fasta-format-file-input.

The given input is split into individual chunks that are processed in a serial manner, while the computations for each chunk are highly parallelized. The chunk size depends on the provided resources. Larger chunks require more memory, while enabling a more effective parallelization. After the chunking, the pipeline starts to loop through the core routine that starts with the sequence retrieval of the input proteins.

Sequence similarity search and alignment

Each of the protein sequences is put into a sequence similarity search against all protein sequences contained in the PDB database. This step is performed by MMseqs2 [39] by setting the sensitivity parameter and the number of returned sequences to its maximum values. This results in a list of potential structure annotations for each input protein. For each potential sequence-to-structure annotation a pairwise global sequence alignment is calculated using the Needleman-Wunsch algorithm [40] with a gap opening penalty of 10 and a gap ex-

tension penalty of 0.5 without any penalty for the end gaps. The sequence identity is computed ignoring the end gaps and should be above 0.35 to accept the corresponding sequence-to-structure annotation. This cutoff is the more conservative end of the usual 30-35 % sequence identity threshold used in automated homology modelling pipelines, and is based on a study of relationship between protein sequence identity and their structural similarity [41]. The accepted alignments are used for position-specific structural annotations, where each amino acid from all input proteins is mapped to a list of specific residues in the experimentally resolved structure.

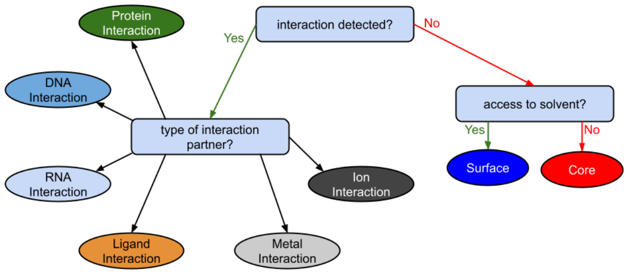

Figure 8. Structural classes are assigned by a decision tree based on the results from the annotation aggregation. The classification aims to describe the functional role of an amino acid residue in the protein structure.

Annotation using AlphaFold models

In contrast to the annotation to experimentally resolved protein structures, we use one AlphaFold [28] model per queried protein sequence for structural annotation. These models do not contain interaction partners and cover the whole range of the given sequence, thus there is no benefit in using more than one. Nonetheless, we use MMseqs2 [39] to perform a sequence similarity search in all entries stored in the AlphaFold Protein Structure database [28, 29]. This allows us to use AlphaFold models for proteins, whose corresponding structure is not in the database, but bears significant similarity to proteins in the database, as well as for or mutant protein sequences or proteins from newly sequenced species.

Structural analysis of individual structures

Each protein structure or multi-protein complex in a PDB entry that contains at least one annotated residue gets completely structurally analysed, unless processed in lite mode. This means that for each residue in each protein chain contained in the entry a wide array of structural features are calculated, such as solvent accessibility, interactions to other residues and molecules present in the structure. The general aim of these features is to specify the functional role of the residue in the structure. Some features describe the location of the residue in the chain, while other features are based on the distances and chemical interactions to other molecules contained in the entry. Some features require a graph-based representation of the corresponding protein complex structures, for which residue interaction networks (RINs) of the corresponding complexes are computed using RINerator [42]. In RINs, each amino acid is represented as a node, and an edge connects two nodes if there is a covalent bond or a non-covalent interaction between them. A complete list of calculated features is given in the supplementary materials (Supplementary table S3).

Aggregation of annotation results from multiple structures

The same set of features is calculated for each residue in each annotated structure, so that a position that is mapped to residues from multiple structures is assigned a list of feature vectors. Further, we calculate a quality score for each annotation, based on the sequence identity, coverage, and the resolution of the annotated structure. Numerical features can then be aggregated by a weighted mean:

$$W(D) = \frac{\Sigma q_i \dot{d}_i}{\Sigma q_i}, i \in D \tag{1}$$

where q_i is the quality score [27] of a structural annotation i; d_i is the individual numerical value from the analysis of the structural annotation i; D is the set of all structural annotations for one input

Since the feature vectors are sparse, the undefined values are not included in the calculation of the weighted sum. This is typical for features that derive from interactions, since the interaction partner might be missing in some of the annotated structures. Here, we aggregate the feature values only from the structures that participate in the interaction. A typical example for aggregating results from multiple structure annotations being advantageous would be multiple structures co-crystallized with different interaction partners. The results aggregation for solvent accessibility values is weighted towards buried residues by multiplying the corresponding quality scores by squared alignment coverage, which introduces a penalty for partially resolved structures. In partially resolved structures, residues might appear to lie on the surface of a protein, because a part of the protein is missing in the experiment, thus when mapping an amino acid to multiple structures with the result that it is annotated as buried residues as well as as surface, we bias the annotations to buried residues as described above.

Structural classification

To provide succinct information, StructMAn assigns a structural class to every queried position (see figure 8). The idea behind the classification is to give a human-readable interpretation for the functional role of a particular amino acid residue in the protein structure. Therefore, we first determine if the amino acid is part of an interaction interface. If this is the case, the classification is set to the type of the interaction partner: protein, DNA, etc. Otherwise, the structural class is "core" for residues buried in the protein and "surface" for those with access to the surrounding solvent but not engaged in interactions. This classification is made by considering the aggregated relative solvent accessible area (RSA) of all residues to which the query position is mapped, as described above. The particular threshold for the aggregated values to make the decision reflects the threshold one would use making the decision for a single structure, since the aggregated values occupy the same scale as the individual values. Here we employ a threshold derived by Rost and Sander [43]: RSA > 0.16 means "surface" and RSA $\leq$ 0.16 means "core".

Aggregation of annotation results for indels

For an indel, two version of the query protein are annotated with the d-StructMAn pipeline: the wild-type and the mutant with the indel (see figure 9). For both protein versions we retrieve feature lists for the positions that are part of the indel region and of the two flanks (half the length of the indel). These

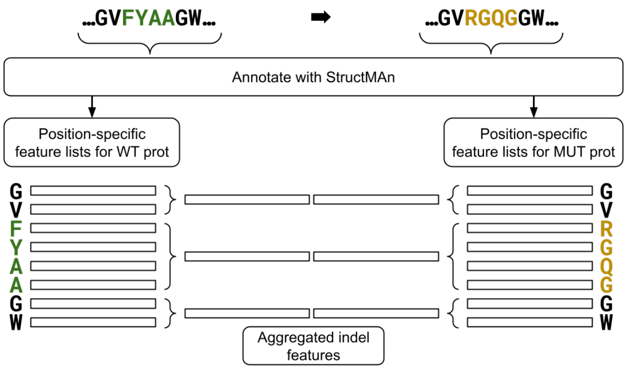

Figure 9. The results aggregation for indels is based on the position-specific results aggregation of the wild type (WT) protein sequence and the results aggregation of the mutant (MUT) protein sequence. For both protein variants, three separate aggregations are performed: left flank of indel, indel region, and right flank of indel. Note that for a insertion the length for the indel region in the WT is zero and hence only the flanks produce feature lists (vice versa for deletions and MUT).

six sets of feature lists can then be aggregated again in the same fashion as we aggregate annotation results from multiple structures and after concatenating them we receive the feature list for an indel.

Implementation

d-StructMAn is provided as a Docker image that can be easily pulled from DockerHub. This image can be used in all architectures/operating systems supported by the Docker engine. The image was also tested for use with Podman (on Fedora 35) which allows users to run containers without root privileges. This would allow running this image in HPC clusters/platforms. The use of containerization allows seamless installation of all the dependencies and databases. Folders are bind-mounted into the volumes that were created inside the container to make sure that the data is persistent. The computational pipeline depends on large amounts of data retrieved from Uniprot and PDB. Online retrieval of the data can slow down computations, not only due to transferred data packages, but also due to high input/output caused by many writings of temporary files to the disk. Therefore, the installation of d-StructMAn can be expanded. The container includes scripts for downloading a local instance of the PDB and Uniprot, installation of RINerator and pre-computing RINs for all structures in the PDB that will be saved to a local database.

Module-wise expansion

Installing StructMAn as a container (4Gb of diskspace) enables all basic functionalities, however in order to reach the highest possible performance StructMAn has to offer, additional modules have to be installed and are described in more details below. The extensions require additional disk space: 100Gb for PDB, 35Gb for RINdb, and 50Gb for Uniprot.

MySQL database

The database stores all data produced by the computational pipeline. This has multiple benefits: first, for multiple runs that share the same proteins and/or structures, a lot of computations can be saved. Second, a similar saving is achieved when running large inputs. Since large inputs are chunked down, and processed in series by the pipeline, identical computations may appear within the same run. For example, for the annotation of the human proteome, the task was chunked into 115 parts. Overall, over 11 million individual protein-to-structure annotations were processed, while the total number of different PDB entries considered was just around 80,000. Without the database, many of the annotations would have led to a repeated analysis of the same structures again and again. With the database, each structure gets analysed exactly one time. The third benefit is the exportation capability of the database enabling easy shipment of StructMAn results between individual instances of the pipeline. An empty instance of the the database based on MariaDB engine is installed automatically in the docker container. For the pip version, the user needs to provide a MySQL database server. Then after the configuration of the database credentials, a single command sets up the database structure. This allows the usage of a database server that is physically apart from the system that runs the pipeline, thus enabling more effective usage of provided resources.

Local instance of the PDB

Throughout the structural analysis section of the pipeline, many thousands of PDB entries may have to be retrieved. The vanilla version takes that data directly from the RCSB webservices resulting in a lot of traffic and time delay. Many work groups oriented towards protein structure analysis already maintain an instance of the PDB locally, while other users can use a script attached to d-StructMAn that downloads and configures an instance of the PDB. The same script can be used to update the local file storage and the search index databases that are required for the sequence similarity search section of the pipeline.

Local instance of the AlphaFold Protein Structure Database

In addition to the PDB, the AlphaFold Protein Structure Database can be used as a source for protein 3D structure data. d-StructMAn contains a script that creates a local instance of the database retrieving all structures stored at https://ftp.ebi.ac.uk/pub/databases/alphafold/latest/ [44]. The database is unpacked and distributed similarly to the PDB directory scheme to ensure fast local retrieval of individual protein structures. At present, d-StructMAn offers the annotation using AlphaFold structures only when the local instance has been installed.

Local instance of a RIN database

An important part of the structural analysis is based on the RIN (residue interaction network) of the corresponding PDB entry. While a live computation of each RIN during the runtime is possible, users that plan the processing of many or large inputs may consider to precompute the RINs of all PDB entries. We provided a script that creates such a RIN database locally. Similar to the local instance of the PDB, the same script can be used to update the RIN database.

Protein sequence and protein identifier mapping database

Different protein or transcript identifiers from different sequence databases can represent an identical protein sequence. Since d-StructMAn operates on the amino acid sequence level, identifying such duplicates at the beginning of the pipeline can save a lot of computations. Uniprot hosts a comprehensive identifier mapping service (https://www.uniprot.org/uploadlists/ [45]) that can also be programmatically accessed. After checking for duplicates the sequences have to be retrieved. Both tasks require a non-blocked connection to the internet and produce some traffic, while one also depends on the Uniprot webservices to be reachable. As an optional upgrade we offer the solution to download all sequences in Uniprot and the identifier mapping tables. They then are locally processed into an SQL database for fast retrieval.

Scaling solutions

Size and complexity of inputs given to StructMAn can vary a lot. This results in various scaling issues that had to be solved in a way considering the resources provided by the underlying system architecture. We provide several solutions to optimally balance the core load and memory usage.

Input chunking

The major mechanism to control memory consumption is the input chunking that divides the input in equally-sized subtasks. The size of a chunk is defined by the number of proteins it contains. It increases when more memory is available, while it decreases when more cores are provided. Larger proteins or those that will be mapped to more structures generate more load on the system. Since the number of proteins only roughly estimates the true complexity of a subtask, the input chunking may be very tentative. The implemented parallelization techniques that are explained in the following subsection enable an efficient core usage even when the number of given proteins is lower than the number of available cores.

Parallelization of the alignment section

For each protein-to-structure mapping coming out of the initial sequence similarity search, a Needleman-Wunsch alignment without terminal gap penalties with runtime complexity $O(nm)$, where n is the length of the protein sequence and m is the length of sequence retrieved from the mapped structure, is computed. Since at this stage of the pipeline both sequence lengths are known, the necessary runtimes for each task can be estimated quite precisely. We use this to prepare N equally complex subtasks, where N is the amount of available cores. In this packaging process we also prefer putting alignment task from the same protein together in order to reduce data amounts shared to the resulting subprocesses. In summary, this leads to a packaging strategy that adapts to different types of inputs. An input carrying a single protein will distribute multiple alignments of the same protein to different structures to different subprocesses and inputs with multiple proteins combine alignments of the same protein together and assign them together to a single subprocess.

Parallelization of the structural analysis

The structural analysis is performed separately for different PDB entries. The complexity of most computations is linear by the number of protein chains in the entry, with the exception of analyses that revolve around interaction between different chains, which have a roughly quadratic complexity. While generally we can distribute the analyses of different entries to different subprocesses, this can lead to situations, where the computation time for a large entry in a separate subprocess takes longer than the computation of all entries. To avoid such scenarios, we assign multiple threads to larger structures and subdivide many of the analyses chain-wise. This is a so-called nested parallelization and should be avoided if possible, but in our case it provided a significant performance increase in practice.

Parallelization of the results aggregation

The structural analysis results from many different structures are combined for each position, this typically requires a lot of resources to look up data from one big data structure. Such a task cannot be effectively parallelized in Python without copying the datastructure for each subprocess, which leads to an overhead that is larger than the time saved by parallelized processing. The Ray [46] library offers solutions for exactly such problem settings; however, when we increase the number of threads the overhead also increases until we reach a point where more threads do not result in any performance increase anymore. For large jobs, we implemented an advanced optimization technique which divides the major data structure at a preprocessing step and sends the substructures to individual subprocesses, which then use the usual parallelization leading again to a nested parallelization setup that can then use a large number of available cores efficiently. Since the preprocessing step also leads to a significant overhead, this technique is best applicable in situations where a large input meets large computation resources.

Availability of source code and requirements

The code is available on GitHub, https://github.com/kalininalab/d-StructMAn. The implementation is in Python and the code is distributed under the LGPL-2.1 licence. Easy installation is provided using containerization software: Docker or Podman.

- Project name: d-StructMAn
- Project home page: https://github.com/kalininalab/d-StructMAn
- Operating system: Linux
- Programming language: Python 3.8
- Other requirements: Docker or Podman
- License: LPGL-2.1
- RRID: SCR_022534
- biotools ID: d-structman

Availability of supporting data and materials

The full structural annotation of the human proteome and ClinVar mutations are openly available in the GigaScience repository, GigaDB [47].

Declarations

List of abbreviations

- 3D: three-dimensional
- DelIns: mutli-residue substitutions
- Gb: gigabyte
- HPC: high performance computing
- indels: insertions and deletions
- MUT: mutant
- PDB: the Protein Data Bank
- RAM: random access memory
- RIN: residue interaction network
- RSA: relative solvent accessible area
- WT: wild type

Consent for publication

“Not applicable”

Competing Interests

The authors declare no competing interests.

Funding

The reported study was funded by the joint RFBR and DFG research project 20-54-12008. A.G. was supported by the German Federal Ministry of Education and Research (BMBF) within the frame-work of the e:Med research and funding concept (grant SysCARE [01ZX1908A]). S.K. was supported by the IMPRS-CS graduate student fellowship and Deutsche Forschungsgemeinschaft (DFG) project number 430158625. S.K.S. was partially supported by the UdS-HIPS-Tandem Interdisciplinary Graduate School for Drug Research. O.V.K. was supported by the Klaus Faber Foundation.

Author's Contributions

A.G. devised the method and implemented the core functionality. S.K.S. and S.K. assisted with the method development and implemented the container. V.R. and O.V.K. conceived the project. All authors wrote the manuscript.

Acknowledgements

References

1. Consortium TGP. A map of human genome variation from population scale sequencing. Nature 2010 Oct;467(7319):1061–1073. https://www.ncbi.nlm.nih.gov/pmc/articles/PMC3042601/.
2. Eilbeck K, Quinlan A, Yandell M. Settling the score: variant prioritization and Mendelian disease. Nature Reviews Genetics 2017 Oct;18(10):599–612. https://www.nature.com/articles/nrg.2017.52, number: 10 Publisher: Nature Publishing Group.
3. Chen R, Mias GI, Li-Pook-Than J, Jiang L, Lam HYK, Chen R, et al. Personal Omics Profiling Reveals Dynamic Molecular and Medical Phenotypes. Cell 2012;148(6):1293–1307. https://www.sciencedirect.com/science/article/pii/S0092867412001663.
4. Amoah K, Hsiao YHE, Bahn JH, Sun Y, Burghard C, Tan BX, et al. Allele-specific alternative splicing and its functional genetic variants in human tissues. Genome Research 2021 Mar;31(3):359–371. https://genome.cshlp.org/content/31/3/359, company: Cold Spring Harbor Laboratory Press Distributor: Cold Spring Harbor Laboratory Press Institution: Cold Spring Harbor Laboratory Press Label: Cold Spring Harbor Laboratory Press Publisher: Cold Spring Harbor Lab.
5. Chong J, Buckingham K, Jhangiani S, Boehm C, Sobreira N, Smith J, et al. The Genetic Basis of Mendelian Phenotypes: Discoveries, Challenges, and Opportunities. The American Journal of Human Genetics 2015 Aug;97(2):199–215. http://www.sciencedirect.com/science/article/pii/S0002929715002451.
6. Landrum MJ, Lee JM, Benson M, Brown G, Chao C, Chitipiralla S, et al. ClinVar: public archive of interpretations of clinically relevant variants. Nucleic Acids Research 2016 Jan;44(Database issue):D862–D868. https://www.ncbi.nlm.nih.gov/pmc/articles/PMC4702865/.
7. Lek M, Karczewski KJ, Minikel EV, Samocha KE, Banks E, Fennell T, et al. Analysis of protein-coding genetic variation in 60,706 humans. Nature 2016 Aug;536(7616):285–291. https://www.nature.com/articles/nature19057.
8. Ramensky V, Bork P, Sunyaev S. Human non-synonymous SNPs: server and survey. Nucleic Acids Research 2002 Sep;30(17):3894–3900. https://academic.oup.com/nar/article/30/17/3894/2376118, publisher: Oxford Academic.
9. Adzhubei IA, Schmidt S, Peshkin L, Ramensky VE, Gerasimova A, Bork P, et al. A method and server for predicting damaging missense mutations. Nature methods 2010 Apr;7(4):248–249. https://www.ncbi.nlm.nih.gov/pmc/articles/PMC2855889/.
10. Ng PC, Henikoff S. SIFT: predicting amino acid changes that affect protein function. Nucleic Acids Research 2003 Jul;31(13):3812–3814. https://www.ncbi.nlm.nih.gov/pmc/articles/PMC168916/.
11. Thomas PD, Campbell MJ, Kejariwal A, Mi H, Karlak B, Daverman R, et al. PANTHER: A Library of Protein Families and Subfamilies Indexed by Function. Genome Research 2003 Sep;13(9):2129–2141. https://www.ncbi.nlm.nih.gov/pmc/articles/PMC403709/.
12. Yue P, Melamud E, Moult J. SNPs3D: Candidate gene and SNP selection for association studies. BMC Bioinformatics 2006 Mar;7:166. https://www.ncbi.nlm.nih.gov/pmc/articles/PMC1435944/.
13. Bromberg Y, Rost B. SNAP: predict effect of non-synonymous polymorphisms on function. Nucleic Acids Research 2007 Jun;35(11):3823–3835. https://www.ncbi.nlm.nih.gov/pmc/articles/PMC1920242/.
14. Thusberg J, Olatubosun A, Vihinen M. Performance of mutation pathogenicity prediction methods on missense variants. Human Mutation 2011;32(4):358–368. https://onlinelibrary.wiley.com/doi/abs/10.1002/humu.21445.
15. De Baets G, Van Durme J, Reumers J, Maurer-Stroh S, Vanhee P, Dopazo J, et al. SNPeffect 4.0: on-line prediction of molecular and structural effects of protein-coding variants. Nucleic Acids Research 2012 Jan;40(Database issue):D935–D939. https://www.ncbi.nlm.nih.gov/pmc/articles/PMC3245173/.
16. Katsonis P, Lichtarge O. A formal perturbation equation between genotype and phenotype determines the Evolutionary Action of protein-coding variations on fitness. Genome Research 2014 Dec;24(12):2050–2058. https://www.ncbi.nlm.nih.gov/pmc/articles/PMC4248321/.
17. Capriotti E, Calabrese R, Fariselli P, Martelli PL, Altman RB, Casadio R. WS-SNPs&GO: a web server for predicting the deleterious effect of human protein variants using functional annotation. BMC Genomics 2013 May;14(Suppl 3):S6. https://www.ncbi.nlm.nih.gov/pmc/articles/PMC3665478/.
18. Ioannidis NM, Rothstein JH, Pejaver V, Middha S, McDonnell SK, Baheti S, et al. REVEL: An Ensemble Method for Predicting the Pathogenicity of Rare Missense Variants. The American Journal of Human Genetics 2016;99(4):877–885. https://www.sciencedirect.com/science/article/pii/S0002929716303706.
19. Li J, Zhao T, Zhang Y, Zhang K, Shi L, Chen Y, et al. Performance evaluation of pathogenicity-computation methods for missense variants. Nucleic Acids Research 2018 Sep;46(15):7793–7804. https://www.ncbi.nlm.nih.gov/pmc/articles/PMC6125674/.
20. Mosca R, Tenorio-Laranga J, Olivella R, Alcalde V, Céol A, Soler-López M, et al. dSysMap: exploring the edgetic role of disease mutations. Nature Methods 2015 Mar;12(3):167–168. https://www.nature.com/articles/nmeth.3289.
21. Betts MJ, Lu Q, Jiang Y, Drusko A, Wichmann O, Utz M, et al. Mechismo: predicting the mechanistic impact of mutations and modifications on molecular interactions. Nucleic Acids Research 2015 Jan;43(2):e10. https://www.ncbi.nlm.nih.gov/pmc/articles/PMC4333368/.
22. Dehiya V, Thomas J, Sael L. Impact of structural prior knowledge in SNV prediction: Towards causal variant finding in rare disease. PLoS ONE 2018 Sep;13(9). https://www.ncbi.nlm.nih.gov/pmc/articles/PMC6161878/.
23. Konc J, Skrlj B, Erzen N, Kunej T, Janezic D. GenProBiS: web server for mapping of sequence variants to pro-

tein binding sites. Nucleic Acids Research 2017 Jul;45(Web Server issue):W253–W259. https://www.ncbi.nlm.nih.gov/pmc/articles/PMC5570222/.
24. Radusky L, Modenutti C, Delgado J, Bustamante JP, Vishnopolska S, Kiel C, et al. VarQ: A Tool for the Structural and Functional Analysis of Human Protein Variants. Frontiers in Genetics 2018 Dec;9. https://www.ncbi.nlm.nih.gov/pmc/articles/PMC6291447/.
25. Wagih O, Galardini M, Busby BP, Memon D, Typas A, Beltrao P. A resource of variant effect predictions of single nucleotide variants in model organisms. Molecular Systems Biology 2018 Dec;14(12). https://www.ncbi.nlm.nih.gov/pmc/articles/PMC6301329/.
26. Segura J, Sanchez-Garcia R, Sorzano COS, Carazo JM. 3DBIONOTES v3.0: crossing molecular and structural biology data with genomic variations. Bioinformatics 2019 Sep;35(18):3512–3513. https://academic.oup.com/bioinformatics/article/35/18/3512/5320560, publisher: Oxford Academic.
27. Gress A, Ramensky V, Büch J, Keller A, Kalinina OV. StructMAn: annotation of single-nucleotide polymorphisms in the structural context. Nucleic Acids Research 2016 Jul;44(W1):W463–468.
28. Jumper J, Evans R, Pritzel A, Green T, Figurnov M, Ronneberger O, et al. Highly accurate protein structure prediction with AlphaFold. Nature 2021 Aug;596(7873):583–589. https://www.nature.com/articles/s41586-021-03819-2, number: 7873 Publisher: Nature Publishing Group.
29. Varadi M, Anyango S, Deshpande M, Nair S, Natassia C, Yordanova G, et al. AlphaFold Protein Structure Database: massively expanding the structural coverage of protein-sequence space with high-accuracy models. Nucleic Acids Research 2021 Nov;50(D1):D439–D444. https://www.ncbi.nlm.nih.gov/pmc/articles/PMC8728224/.
30. UniProt: a hub for protein information. Nucleic Acids Research 2015 Jan;43(Database issue):D204–D212. https://www.ncbi.nlm.nih.gov/pmc/articles/PMC4384041/.
31. human in UniProtKB (6971127) | UniProt;. https://www.uniprot.org/uniprotkb/?query=human&fil=proteome%3AUP000005640+AND+organism%3A%22Homo+sapiens+%28Human%29+%5B9606%5D%22&sort=score.
32. Index of /pub/clinvar/tab_delimited;. https://ftp.ncbi.nlm.nih.gov/pub/clinvar/tab_delimited/.
33. Pruitt K, Brown G, Tatusova T, Maglott D. The Reference Sequence (RefSeq) Database. National Center for Biotechnology Information (US); 2012. https://www.ncbi.nlm.nih.gov/books/NBK21091/.
34. Erdős G, Pajkos M, Dosztányi Z. IUPred3: prediction of protein disorder enhanced with unambiguous experimental annotation and visualization of evolutionary conservation. Nucleic Acids Research 2021 05;49(W1):W297–W303. https://doi.org/10.1093/nar/gkab408.
35. Schwarz JM, Cooper DN, Schuelke M, Seelow D. MutationTaster2: mutation prediction for the deep-sequencing age. Nature Methods 2014 Apr;11(4):361–362. https://www.nature.com/articles/nmeth.2890.
36. Sundaram L, Gao H, Padigepati SR, McRae JF, Li Y, Kosmicki JA, et al. Predicting the clinical impact of human mutation with deep neural networks. Nature Genetics 2018 Aug;50(8):1161. https://www.nature.com/articles/s41588-018-0167-z.
37. Boudellioua I, Kulmanov M, Schofield PN, Gkoutos GV, Hoehndorf R. DeepPVP: phenotype-based prioritization of causative variants using deep learning. BMC Bioinformatics 2019 Feb;20(1):65. https://doi.org/10.1186/s12859-019-2633-8.
38. Gress A, Ramensky V, Kalinina OV. Spatial distribution of disease-associated variants in three-dimensional structures of protein complexes. Oncogenesis 2017 Sep;6(9):e380.
39. Steinegger M, Söding J. MMseqs2 enables sensitive protein sequence searching for the analysis of massive data sets. Nature Biotechnology 2017 Oct;35:1026–1028. https://www.nature.com/articles/nbt.3988.
40. Needleman SB, Wunsch CD. A general method applicable to the search for similarities in the amino acid sequence of two proteins. Journal of Molecular Biology 1970;48(3):443–453. https://www.sciencedirect.com/science/article/pii/0022283670900574.
41. Rost B. Twilight zone of protein sequence alignments. Protein Engineering, Design and Selection 1999 Feb;12(2):85–94. https://academic.oup.com/peds/article/12/2/85/1550637.
42. Doncheva NT, Klein K, Domingues FS, Albrecht M. Analyzing and visualizing residue networks of protein structures. Trends in Biochemical Sciences 2011 Apr;36(4):179–182. http://www.sciencedirect.com/science/article/pii/S0968000411000132.
43. Rost B, Sander C. Conservation and prediction of solvent accessibility in protein families. Proteins: Structure, Function, and Bioinformatics 1994;20(3):216–226. https://onlinelibrary.wiley.com/doi/abs/10.1002/prot.340200303.
44. Index of /pub/databases/alphafold/latest/;. https://ftp.ebi.ac.uk/pub/databases/alphafold/latest/.
45. Retrieve/ID mapping | UniProt;. https://www.uniprot.org/id-mapping/.
46. Moritz P, Nishihara R, Wang S, Tumanov A, Liaw R, Liang E, et al. Ray: A Distributed Framework for Emerging AI Applications. arXiv:171205889 [cs, stat] 2018 Sep;http://arxiv.org/abs/1712.05889, arXiv: 1712.05889.
47. Decap D, de Schaetzen van Brienen L, Larmuseau M, Costanza P, Herzeel C, Wuyts R, et al. Halvade somatic: Somatic variant calling with Apache Spark. GigaScience 2022 Jan;11:giab094. https://academic.oup.com/gigascience/article/doi/10.1093/gigascience/giab094/6505120.

Manuscript

Click here to access/download;Manuscript;dStructMAn_for_GigaScience_Jo

GigaScience, 2017, 1–11

doi: xx.xxxx/xxxx
Manuscript in Preparation
Paper

PAPER

d-StructMAn: containerized structural annotation on the scale from genetic variants to whole proteomes

Alexander Gress[1,2,*], Sanjay K. Srikakulam[1,2,3], Sebastian Keller[1,2,4], Vasily Ramensky[5,6] and Olga V. Kalinina[1,7,8]

[1]Helmholtz Institute for Pharmaceutical Research Saarland (HIPS) / Helmholtz Centre for Infection Research (HZI), Saarbrücken, Germany and [2]Graduate School of Computer Science, University of Saarland, Saarbrücken, Germany and [3]Interdisciplinary Graduate School of Natural Product Research, Saarland University, Saarbrücken, Germany and [4]Research Group Computational Biology, Max Planck Institute for Informatics, Saarbrücken, Germany and [5]National Medical Research Center for Therapy and Preventive Medicine of the Ministry of Healthcare of Russian Federation, Moscow, Russia and [6]Faculty of Bioengineering and Bioinformatics, Lomonosov Moscow State University, Moscow, Russia and [7]Medical Faculty, Saarland University, Homburg, Germany and [8]Center for Bioinformatics, Saarland Informatics Campus, Saarbrücken, Germany

[*]alexander.gress@helmholtz-hips.de

Abstract

Background, Structural annotation of genetic variants in the context of inter-molecular interactions and protein stability can shed light onto mechanisms of disease-related phenotypes. Three-dimensional structures of related proteins in complexes with other proteins, nucleic acids, or ligands enriches such functional interpretation, since inter-molecular interactions are well conserved in evolution. **Results**, We present d-StructMAn, a novel computational method that enables structural annotation of local genetic variants, such as single-nucleotide variants and in-frame indels, and implements it in a highly efficient and user-friendly tool provided as a Docker container. Using d-StructMAn, we annotated several very large sets of human genetic variants, including all variants from ClinVar and all amino acid positions in the human proteome. We were able to provide annotation for more than 46% of positions in the human proteome representing over 60% proteins. **Conclusions**, d-StructMAn is the first of its kind and highly efficient tool for structural annotation of protein-coding genetic variation in the context of observed and potential inter-molecular interactions. d-StructMAn is readily applicable to proteome-scale datasets and can be instrumental building machine-learning tools for predicting genotype-to-phenotype relationships.

Key words: Single-nucleotide variants, indels, genetic variation, protein structure, protein interactions, structural annotation, Docker container

Introduction

Background

In the age of next-generation sequencing, large-scale genetic diversity within populations became apparent. A single human individual of European ancestry carries around three million genetic variants [1], of which up to 14,000 occur in coding regions and lead to an amino acid substitution [2]. Additionally, up to 3,000 short insertions or deletions (indels) occur in the coding regions and either lead to a frame shift or cause an indel in the corresponding protein sequence [3]. These coding variants that result in non-synonmous substitutions or indels in the protein sequence are the focus of this study. Additional sources of indels are alternatively spliced isoforms that result in retaining or skipping exons or parts of introns in the translated sequence. Alternative splicing is wide-

Compiled on: July 6, 2022.
Draft manuscript prepared by the author.

Key Points

- A novel bioinformatics tool for structural characterization of genetic variants is presented
- Single-nucleotide variants and indels are described with respect to inter-molecular interactions in homologous protein complexes
- An efficient implementation using Docker container allows for analysis of large whole proteome-scale datasets

spread across eukaryotes and human tissues [4]. UniProt lists an average of approximately four isoforms per a protein-coding gene in its reference human proteome.

Although most of sequence variants have no functional or pathogenic effect, in some cases even a single mutation can be disease-causing [5, 6]. Experimental characterization of all variants is infeasible; since most of them are rare and occur in only one or few individuals [7], statistical characterization of their association with diseases seems impossible, too. This emphasizes the importance of computational tools for predicting functional and/or pathogenic effects of sequence variants. Many such tools were developed specifically for non-synonymous (missense) variants and take into account the protein three-dimensional (3D) structure context of an input variant. One of the keystones in this field is PolyPhen [8, 9] which combines a number of features related to protein sequence and 3D structure in a Bayes classifier that predicts an individual variant to be deleterious or benign. Other methods also rely on conservation of the mutated position in a protein alignment [10, 11], or use additional features derived from phylogenetic analysis or analysis of the protein 3D structure [12, 13, 14, 15, 16]. Meta-methods combine outputs from several prediction tools [17, 18, 19].

Of those tools that employ protein 3D structure to derive various predictive features, several put variants of interest in the context of protein-protein interactions [20], and interactions with other biologically relevant molecules [21]. Indeed, features related to protein three-dimensional structure were proven to be instrumental for predicting pathogenic effects of mutations [22]. Various intricate facets of structural analysis have also been implemented in databases and computational tools [23, 24, 25, 26] with one common drawback: they rely on information from individual structures and do not integrate the multiple relevant findings.

As far as structural annotation of larger variants is concerned, the majority of methods are developed to annotate single amino acid replacements, and, to the best of our knowledge, there is currently no structural annotation pipeline that is able to map indel-type genetic variants to protein 3D structures.

High-performance structural annotation

In this study, we present d-StructMAn, a new improved implementation of our earlier tool StructMAn[27], shipped in a convenient and easily installable container form and extended to annotation of short in-frame indels and alterations arising as a consequence of alternative splicing events. d-StructMAn produces a wide range of structural features by combining information from experimentally resolved structures of many related proteins, which is a unique feature of the StructMAn family tools. In addition to experimentally resolved protein 3D structures, d-StructMAn can also harness information from all protein structure models stored in the AlphaFold Protein Structure Database [28, 29]. To the best of our knowledge, this is the first tool with this property that can also analyze genomic indels and consequences of alternative splicing events. Additionally, we provide structural annotation of all proteins in the human proteome (all canonical protein sequences and all isoform sequences listed in UniProt) and all pathogenic and benign genetic variants from ClinVar[6] as a publicly available data resource.

Table 1. The ClinVar dataset.

Mutation type	Pathogenic	Benign	None	Total
SAV*	41557	48285	317261	407103
Deletion	1615	775	5389	7779
Insertion	108	82	372	562
DelIns**	287	46	750	1083
All	43567	49188	323772	416527

* Single amino acid variation.
** Multi-residue substitutions

Data Description

Human proteome dataset

The human proteome dataset contains 101,014 protein isoform sequences belonging to 79,038 human protein entries in UniProt [30]. We generated the dataset by downloading (on the 5th December 2021) all sequences from https://www.uniprot.org/uniprot/?query=human&fil=proteome%3AUP000005640+AND+organism%3A%22Homo+sapiens+%28Human%29+%5B9606%5D%22&sort=score# [31] choosing for format: "FASTA (canonical & isoforms)". StructMAn can process this FASTA file directly. However, to test StructMAn's functionality to retrieve sequences in an isoform-specific manner, we have also extracted accession identifiers and used them as an input. The resulting file (Supplementary file S1) represents protein sequence data over 4 million individual amino acids and was used as input file for StructMAn.

ClinVar

The freely accessible ClinVar [6] database contains human mutations annotated with clinical outcomes. We downloaded (on 13th September 2021) the "variant_summary.txt.gz" from https://ftp.ncbi.nlm.nih.gov/pub/clinvar/tab_delimited/ [32]. We retained DelIns (mutli-residue substitutions), insertions or deletions with "Assembly" field equal to GRCh38 and a RefSeq[33] protein identifier provided. The provided "clinical significance" field was simplified to Pathogenic, Benign and Unknown. The resulting dataset is described in the Table 1 and Supplementary file S2.

Analyses

Annotation of the human proteome

The structural annotation of more than 100,000 protein sequences represents size-wise the ultimate challenge for a structural annotation method, but on the other hand reviews the future applicability of the method by estimating what fraction of the human proteome can be mapped to the structure data.

For only 21.7% of all proteins the corresponding experimentally resolved structure is available in the PDB (Figure 1) and since not all of them were resolved in full length this estimate is reduced to 13.9% of the positions. Mapping proteins to structures of homologs drastically increases the usability of structural annotation to 60.5% of all proteins in the human proteome.

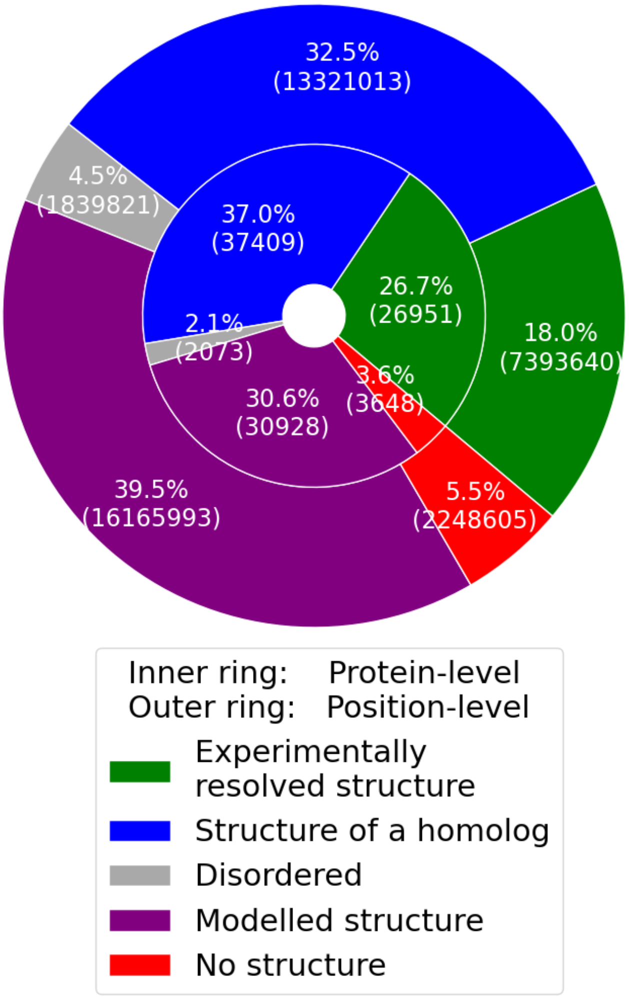

Figure 1. Proportion of proteins and positions from the human proteome dataset that could be mapped to structure data. Experimentally resolved structure (green) denotes that the protein (position) was mapped to at least one structure with sequence identity $\geq$ 0.99, structure of a homolog (blue) denotes that the protein (position) was mapped to at least one structures with sequence identity in the range from 0.35 to 0.99. Modelled structure (purple) denotes that the protein (position) could only be mapped into modelled structure that is not directly supported by experimental data. Disordered (grey) denotes proteins and positions that could not be mapped to any structure, but are predicted by IUPred3 [34] to be disordered (for proteins all positions have to be predicted to be disordered). No structure (red) denotes all other proteins and positions.

When a position can be structurally annotated, it can rarely be mapped to only structure. Most of the times, it is possible to identify more than one structure for a position that can be used for structural annotation (Figure 2). Hence, another challenge of structural annotation is to manage the multiple sources of structural information. In such cases, StructMAn provides the user with recommendations listing the structure with the highest sequence identity and a structure considered to be the most representative biologically. The latter recommendation is based on the the structural analysis of all annotated structures and aggregation of relevant information. Sometimes both structures (maximal identity and recommended) may be the same one.

Another advantage of annotation using all available structures is for proteins with partially resolved structures that cover different regions of the protein. This way, StructMAn could annotate 15% more (46% vs. 31%) positions compared to a strategy, where only the structure with maximum sequence identity is chosen (Figure 3).

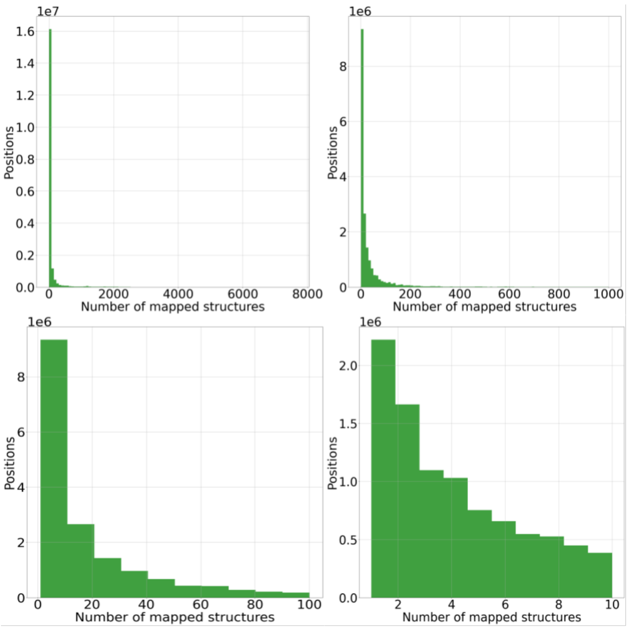

Figure 2. Distribution of the number of structures that could be mapped to a position in the annotation of the human proteome dataset. Each subplot shows the same distribution with a different zoom.

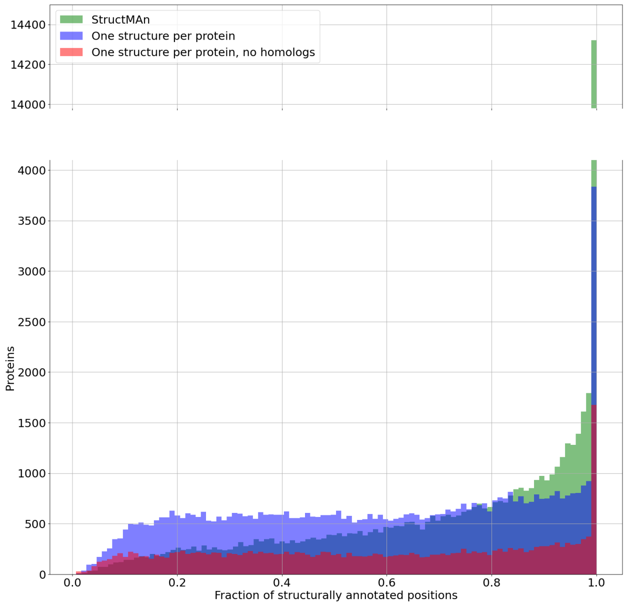

Figure 3. Distribution of the proportion of positions per protein that could be mapped to a structure in the annotation of the human proteome dataset. Green: fraction of positions annotated using multiple structures found by StructMAn; blue: fraction of positions annotated using only one structure per protein (highest sequence similarity was used, in case of same sequence similarity, higher alignment coverage was preferred); red: fraction of positions annotate without considering structures of homologs.

Clinically relevant genetic variations

ClinVar contains genetic variations labeled with their clinical outcome and is routinely used in numerous supervised machine learning methods aimed to predict the effects of genetic variations [35, 22, 36, 37]. This makes ClinVar the ideal testing ground to showcase the feature generation capabilities of StructMAn. It also demonstrates the new indel annotation and analyses that generated features specific to indel-type genetic variations.

We performed structural classification of all positions in the hu-

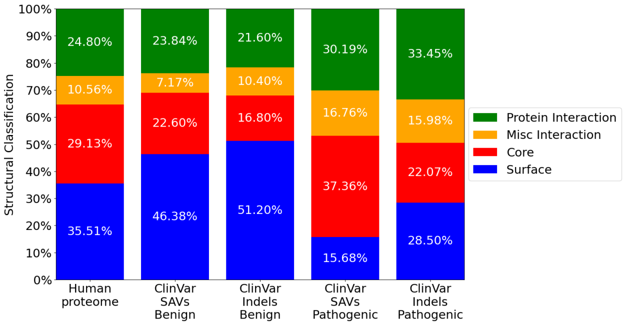

Figure 4. Each stacked barplot denotes the distribution of structural classifications for a dataset. Only positions that could be mapped to at least one structure are considered for this figure. Protein interaction: amino acids that are part of a protein-protein interaction interface; Misc interaction: amino acids that are part of an interaction with a non-protein partner, DNA for example; Core: amino acids in the core of the protein; Surface: amino acids classified to have access to solvent (and not involved in interactions).

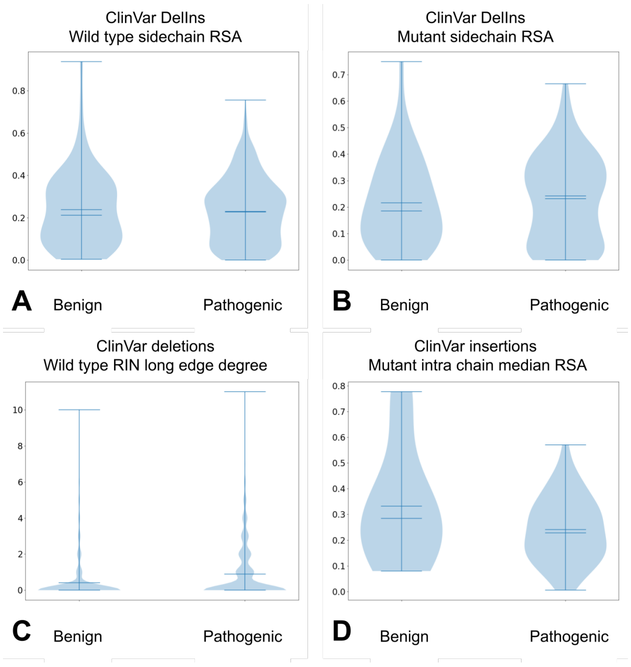

Figure 5. Violin plots for four example features. Left and right plots display the distribution of feature values for benign and pathogenic variants in ClinVar, respectively. **A:** relative surface area (RSA) value for chain atoms in the structures used for annotation of the wild-type protein. Only DelIns (mutli-residue substitutions). **B:** Same as A, but for the structures used for annotation of the mutant protein. **C:** Only deletions. The number of spatial interactions to other amino acids in the same polypeptide chain and separated by more than 6 residues in the sequence. **D:** Only insertions. Median solvent access of residues from other proteins (co-crystallized structures) that lie in a 10Å sphere around the annotated residue.

man proteome and variant positions in the ClinVar dataset (more details in the Methods section) and compared structural classes distribution depending on the ClinVar variant clinical significance (Figure 4). Structural classifications for benign genetic variants, both SAVs and indels, are distributed similarly to the classifications for all amino acids in the human proteome. Pathogenic SAVs have an increased tendency to be located in the protein core. This is also the case for pathogenic indels, but not as strongly as for pathogenic SAVs. Further, pathogenic SAVs and indels are enriched on interaction interfaces. These results are in agreement with our earlier analysis [38], e.g. benign variants are enriched for non-interacting surfaces, whereas pathogenic and disease-associated variants are depleted in these regions, but tend to appear more often on interaction interfaces.

Most missense variant effect prediction methods based on supervised machine learning models rely on ClinVar as the training and testing dataset. We used d-StructMAn to generate 123 features for every SAV and 600 features for every indel in ClinVar (more details about feature generation are provided in the Methods section: for SAVs, see section 'Structural analysis of individual structures', for indels, see section 'Aggregation of annotation results for indels'). Structural features generated for SAVs are based on the results of the structural analysis of the residues in the wild-type, while for indels the wild-type and mutant versions of the protein are analysed separately and used for features generation (see Methods for more details). We selected four example features and plotted their value distributions for benign and pathogenic indels (Figure 5). This gives an example of d-StructMAn employed for the preliminary feature analysis and selection, since a machine learning method would benefit from features that discriminate the datasets of interest.

Performance benchmark

In order to benchmark the runtime performance of StructMAn on different computing systems and different configurations, we generated six datasets. Three of them contain 10, 50 or 100 proteins that all can be mapped to only one or very few PDB entries. Two other datasets contained 10 or 50 proteins that each could be mapped to around 100 PDB entries. The last dataset contains 10 proteins that are very challenging for the pipeline, since they can be mapped to over 13,000 PDB entries, since this dataset includes such proteins as kinases and antibodies. The total number of mapped structures and hence the total number of structurally analysed residues is the main cause for computational complexity. This number does not directly depend on the number of proteins in the dataset (Table 2).

We processed the benchmark datasets on different systems and different configurations. First, these configurations differ in whether we used local resources (local instances of Uniprot and PDB, see Methods for more information). Second, they differ in whether we used the so-called lite mode that switches off the usage of the internal StructMAn database and performs all the calculations and retrievals on the fly. The lite mode still can use local resources, but does not store intermediate results, thus it is faster on smaller inputs, but slower on larger inputs and when run multiple times in succession. All benchmark runs are conducted on an empty StructMAn database instance, thus we only measure the overhead of filling the database and not the amortized benefits one would receive from successive usage of StructMAn, when many intermediate results can be reused. Therefore, lite mode runs always have an advantage over the default mode runs in this benchmark. Runtime was measured in two systems and four different configurations for each dataset (Figure 6).

Table 2. The benchmark datasets. The first three datasets contain proteins that can be mapped to very few structures. The third and fourth datasets contain proteins that could be map to many structures (around 100). The last dataset contains 10 proteins that can be mapped to >13,000 of structures in total.

Dataset	Positions	Mapped PDB entries	Analyzed residues
10 proteins, few hits	1,398	14	7,242
50 proteins, few hits	13,612	70	45,014
100 proteins, few hits	33,789	146	129,465
10 proteins, many hits	2,415	1,216	678,472
50 proteins, many hits	29,726	5,339	2,709,267
10 proteins, very many hits	1,440	13,109	9,753,815

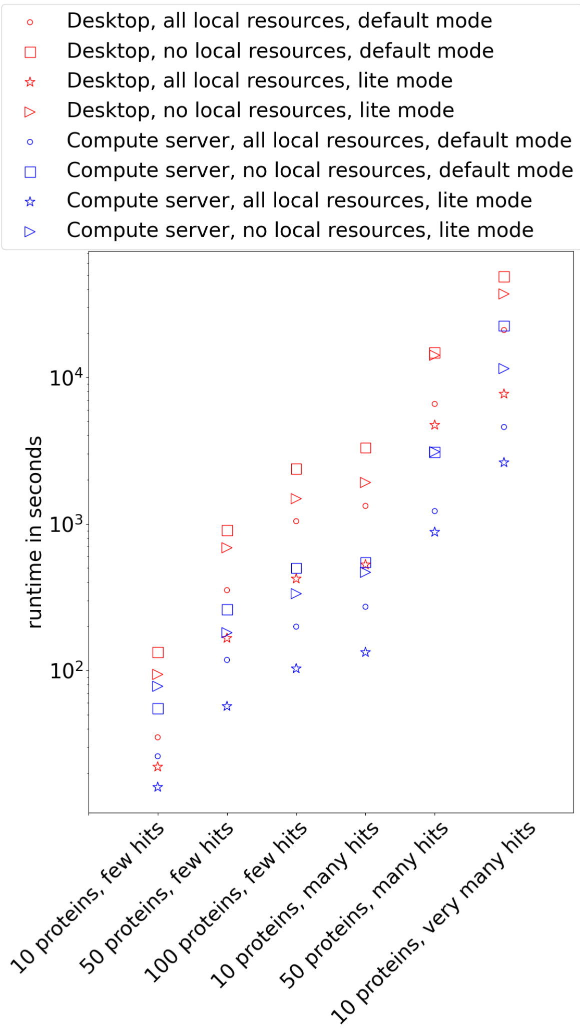

Figure 6. Scatter plot showing runtime performance of StructMAn using different systems and different configurations. Red markers denote a normal desktop computer and blue markers denote a high-performance computing server. Different marker shapes denote different configurations of d-StructMAn.

Table 3. Condensed classification output for position D833 in A2ABF8.

Weighted mainchain location	Core
Weighted sidechain location	Surface
RIN class	Multiple interactions: sidechain contact with a ligand and sidechain contact with a protein
Amount of structures this position is mapped to	291
Recommended structure	2V4H:C 118:D

Output example

d-StructMAn is able to produce very comprehensive outputs, and here we describe an example annotating one SAV D833A in the histone-lysine N-methyltransferase EHMT2 (Uniprot accession: A2ABF8).

Classification table

The classification table presents the integral output of the pipeline and is provided in a tab-separated (.tsv) file format. This table contains 28 values per queried position. The most informative of these values are summarized in Table 3 (we provide the full list of values in Supplementary Table S4).

Table 4. Three examples of feature table entries of protein A2ABF8 D833A.

Sidechain ligand score	0.00707
Sidechain protein score	0.02437
Mainchain protein score	0.01546

From the selected values one can see that the sidechain of the queried amino acid is part of the protein surface, while its mainchain belongs to the core of the protein. Further it participates in interactions with low-molecular-weight ligands and other proteins. We can see that the example position was mapped to 291 different experimentally resolved protein 3D structures and one of them was provided as recommended structure (including the corresponding chain identify and residue number).

Feature table

The feature table is also a tab-separated (.tsv) file containing one row per queried position. It contains all values computed during the structural analysis that could be used as features in a machine learning method. A manual interpretation of the feature table is much harder, thus we focus on three specific feature values (Table 4) to get a deeper insight into the interactions the queried amino acid position engages in. The shown scores reflect the strength of interaction between mainchain atoms or sidechain atoms of the mutated position and different types of interaction partners. The given interaction score between sidechain atoms and low-molecular-weight ligands (0.00707) is relatively low and indicates that the protein-protein interaction is perhaps the more relevant type of interaction in this case. When comparing the protein interaction scores between sidechain atoms (0.02437) and mainchain atoms (0.01546), we can see that the interaction is mitigated more by the sidechain part of the amino acid, which agrees well with the observation that we made regarding surface/core orientation based on the classification table. The overall distribution of interaction scores over all considered proteins (data not shown) indicates that these scores are comparably low, thus while the amino acid clearly participates in the interactions, it might not be the most important player for it.

Discussion

In this study, we presented the structural annotation method d-StructMAn. To our knowledge, it is the first fully automated structural annotation method that can be installed locally and run as a command line tool inside a Docker container. We annotated two big datasets with d-StructMAn: all proteins in human and ClinVar (the annotations are publicly available, see Availability of source code and requirements section). The annotation of the human proteome showed that in practice structural annotation is possible for more than 50% positions, but only if the structural annotation method considers structures of homologs. A great advantage of d-StructMAn is the analysis of all available homologous protein structures in addition to aggregation of the results. These annotations will be useful for scientists in many practical application scenarios.

The performance benchmark confirmed that d-StructMAn runs well on personal laptop and desktop computers. However, for the annotation of large-scale datasets, we would still suggest using a compute server. For instance, the annotation of the human proteome took over 55 hours on our server consuming 250 cores and 1511Gb RAM. The same system processed the most difficult test dataset in the benchmark section in 1.5 hour, while the desktop system (11 cores, 16Gb RAM) took almost 6 hours.

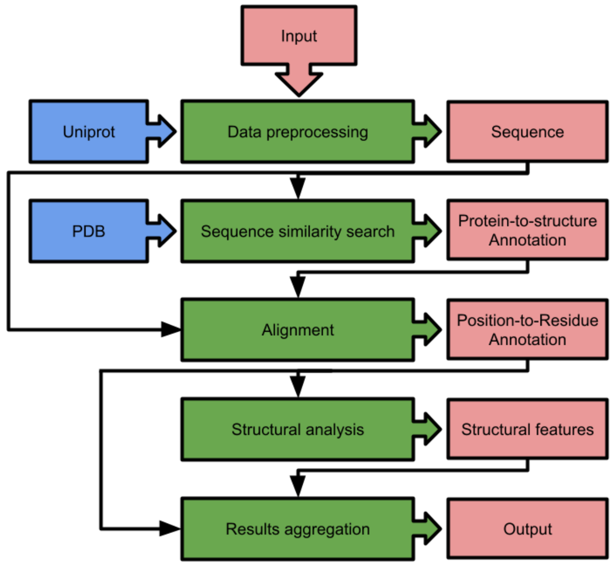

Figure 7. Schematic of computational pipeline of StructMAn. Green boxes are computational sections, red boxes are data structures, and blue boxes are data sources.

Potential implications

The feature vectors generated by d-StructMAn structural analysis for each given protein position are ideal to be fed into complex supervised machine learning methods. The history of the application of protein structure-based features in mutation effect prediction is surprisingly vacant. This is due to two major challenges: First, the computational work and the implementation needed to generate structural features can not be underestimated. This hurdle should now be solved by our containerized structural annotation method. The second challenge is the sparsity of structural features, since they are not available for all positions in all proteins. Here, we implemented a variety of techniques to increase the coverage by considering structures of all homologous proteins and aggregating results from them. The gradual growth of the Protein Data Bank size and the recent developments in the protein structure prediction [28] also help to overcome the problem of incomplete protein sequence space coverage.

Methods

StructMAn is a computational pipeline that combines the retrieval and usage of information from publicly available databases with the application of complex computational biology algorithms. The pipeline can be divided into five computational steps (Figure 7).

Input and preprocessing

Input given to StructMAn can have up to three parts. The first part is mandatory and corresponds to protein sequence data. Protein sequences can be submitted via different kind of protein database identifier (Uniprot, RefSeq, and HGNC), directly given as fasta-formatted sequences, or using PDB entry identifiers. In the latter case the protein sequence is retrieved by parsing the ATOM records of the PDB file. Due to the 'never-compute-anything-twice' policy of StructMAn, it is important to detect protein identifiers that map to the same amino acid sequence. Therefore, the protein identifier mapping services from UniProt are used.

The second part of input can be comprised of positions or genetic variations in the corresponding protein sequence. It is optional, and if it is not set, all positions in the corresponding sequence will be annotated. Possible genetic variations are single amino acid variations, insertions and deletions. The third part of the input are tags, which can be used by users to label specific positions or genetic variations in their input data. These labels will then reappear in the output, and summary statistics for each tag will be calculated to help to use the data for downstream applications. A detailed documentation of the possible input formats for d-StructMAn is provided in the wiki: https://github.com/kalininalab/d-StructMAn/wiki/Simple-Mutation-List-Format and https://github.com/kalininalab/d-StructMAn/wiki/Fasta-format-file-input.

The given input is split into individual chunks that are processed in a serial manner, while the computations for each chunk are highly parallelized. The chunk size depends on the provided resources. Larger chunks require more memory, while enabling a more effective parallelization. After the chunking, the pipeline starts to loop through the core routine that starts with the sequence retrieval of the input proteins.

Sequence similarity search and alignment

Each of the protein sequences is put into a sequence similarity search against all protein sequences contained in the PDB database. This step is performed by MMseqs2 [39] by setting the sensitivity parameter and the number of returned sequences to its maximum values. This results in a list of potential structure annotations for each input protein. For each potential sequence-to-structure annotation a pairwise global sequence alignment is calculated using the Needleman-Wunsch algorithm [40] with a gap opening penalty of 10 and a gap extension penalty of 0.5 without any penalty for the end gaps. The sequence identity is computed ignoring the end gaps and should be above 0.35 to accept the corresponding sequence-to-structure annotation. This cutoff is the more conservative end of the usual 30-35 % sequence identity threshold used in automated homology modelling pipelines, and is based on a study of relationship between protein sequence identity and their structural similarity [41]. The accepted alignments are used for position-specific structural annotations, where each amino acid from all input proteins is mapped to a list of specific residues in the experimentally resolved structure.

Annotation using AlphaFold models

In contrast to the annotation to experimentally resolved protein structures, we use one AlphaFold [28] model per queried protein sequence for structural annotation. These models do not contain interaction partners and cover the whole range of the given sequence, thus there is no benefit in using more than one. Nonetheless, we use MMseqs2 [39] to perform a sequence similarity search in all entries stored in the AlphaFold Protein Structure database [28, 29]. This allows us to use AlphaFold models for proteins, whose corresponding structure is not in the database, but bears significant similarity to proteins in the database, as well as for or mutant protein sequences or proteins from newly sequenced species.

Structural analysis of individual structures

Each protein structure or multi-protein complex in a PDB entry that contains at least one annotated residue gets completely structurally analysed, unless processed in lite mode. This means that for each residue in each protein chain contained in the entry a wide array of structural features are calculated, such as solvent accessibility, interactions to other residues and molecules present in the structure. The general aim of these features is to specify the functional role of the residue in the structure. Some features describe the location of the residue in the chain, while other features are based on the

distances and chemical interactions to other molecules contained in the entry. Some features require a graph-based representation of the corresponding protein complex structures, for which residue interaction networks (RINs) of the corresponding complexes are computed using RINerator [42]. In RINs, each amino acid is represented as a node, and an edge connects two nodes if there is a covalent bond or a non-covalent interaction between them. A complete list of calculated features is given in the supplementary materials (Supplementary table S3).

Aggregation of annotation results from multiple structures

The same set of features is calculated for each residue in each annotated structure, so that a position that is mapped to residues from multiple structures is assigned a list of feature vectors. Further, we calculate a quality score for each annotation, based on the sequence identity, coverage, and the resolution of the annotated structure. Numerical features can then be aggregated by a weighted mean:

$$W(D) = \frac{\Sigma q_i \dot{d}_i}{\Sigma q_i}, i \in D \qquad (1)$$

where q_i is the quality score [27] of a structural annotation i; d_i is the individual numerical value from the analysis of the structural annotation i; D is the set of all structural annotations for one input

Since the feature vectors are sparse, the undefined values are not included in the calculation of the weighted sum. This is typical for features that derive from interactions, since the interaction partner might be missing in some of the annotated structures. Here, we aggregate the feature values only from the structures that participate in the interaction. A typical example for aggregating results from multiple structure annotations being advantageous would be multiple structures co-crystallized with different interaction partners. The results aggregation for solvent accessibility values is weighted towards buried residues by multiplying the corresponding quality scores by squared alignment coverage, which introduces a penalty for partially resolved structures. In partially resolved structures, residues might appear to lie on the surface of a protein, because a part of the protein is missing in the experiment, thus when mapping an amino acid to multiple structures with the result that it is annotated as buried residues as well as as surface, we bias the annotations to buried residues as described above.

Structural classification

To provide succinct information, StructMAn assigns a structural class to every queried position (see figure 8). The idea behind the classification is to give a human-readable interpretation for the functional role of a particular amino acid residue in the protein structure. Therefore, we first determine if the amino acid is part of an interaction interface. If this is the case, the classification is set to the type of the interaction partner: protein, DNA, etc. Otherwise, the structural class is "core" for residues buried in the protein and "surface" for those with access to the surrounding solvent but not engaged in interactions. This classification is made by considering the aggregated relative solvent accessible area (RSA) of all residues to which the query position is mapped, as described above. The particular threshold for the aggregated values to make the decision reflects the threshold one would use making the decision for a single structure, since the aggregated values occupy the same scale as the individual values. Here we employ a threshold derived by Rost and Sander [43]: RSA > 0.16 means "surface" and RSA $\leq$ 0.16 means "core".

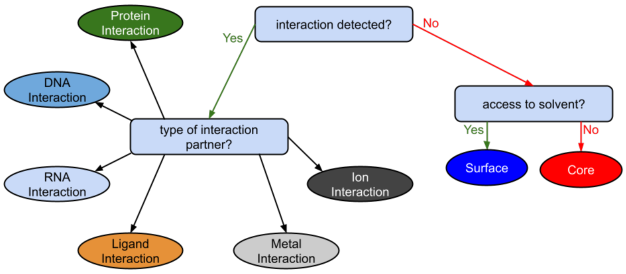

Figure 8. Structural classes are assigned by a decision tree based on the results from the annotation aggregation. The classification aims to describe the functional role of an amino acid residue in the protein structure.

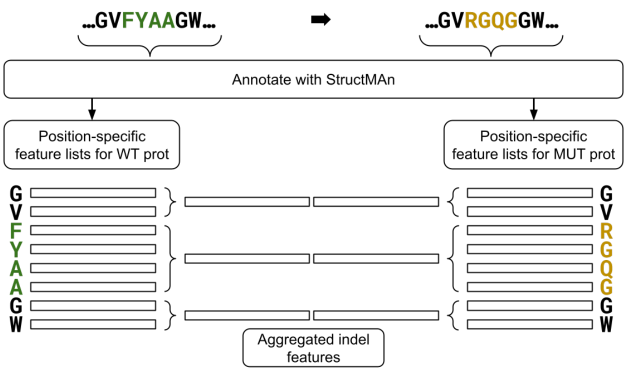

Figure 9. The results aggregation for indels is based on the position-specific results aggregation of the wild type (WT) protein sequence and the results aggregation of the mutant (MUT) protein sequence. For both protein variants, three separate aggregations are performed: left flank of indel, indel region, and right flank of indel. Note that for a insertion the length for the indel region in the WT is zero and hence only the flanks produce feature lists (vice versa for deletions and MUT).

Aggregation of annotation results for indels

For an indel, two version of the query protein are annotated with the d-StructMAn pipeline: the wild-type and the mutant with the indel (see figure 9). For both protein versions we retrieve feature lists for the positions that are part of the indel region and of the two flanks (half the length of the indel). These six sets of feature lists can then be aggregated again in the same fashion as we aggregate annotation results from multiple structures and after concatenating them we receive the feature list for an indel.

Implementation

d-StructMAn is provided as a Docker image that can be easily pulled from DockerHub. This image can be used in all architectures/operating systems supported by the Docker engine. The image was also tested for use with Podman (on Fedora 35) which allows users to run containers without root privileges. This would allow running this image in HPC clusters/platforms. The use of containerization allows seamless installation of all the dependencies and databases. Folders are bind-mounted into the volumes that were created inside the container to make sure that the data is persistent. The computational pipeline depends on large amounts of data retrieved from Uniprot and PDB. Online retrieval of the data can slow down computations, not only due to transferred data packages, but also due to high input/output caused by many writings of temporary files to the disk. Therefore, the installation of d-StructMAn can be expanded. The container includes scripts for downloading a local instance of the PDB and Uniprot, installation of RINerator and pre-computing RINs for all structures in the PDB that will be saved to a local database.

Module-wise expansion

Installing StructMAn as a container (4Gb of diskspace) enables all basic functionalities, however in order to reach the highest possible performance StructMAn has to offer, additional modules have to be installed and are described in more details below. The extensions require additional disk space: 100Gb for PDB, 35Gb for RINdb, and 50Gb for Uniprot.

MySQL database

The database stores all data produced by the computational pipeline. This has multiple benefits: first, for multiple runs that share the same proteins and/or structures, a lot of computations can be saved. Second, a similar saving is achieved when running large inputs. Since large inputs are chunked down, and processed in series by the pipeline, identical computations may appear within the same run. For example, for the annotation of the human proteome, the task was chunked into 115 parts. Overall, over 11 million individual protein-to-structure annotations were processed, while the total number of different PDB entries considered was just around 80,000. Without the database, many of the annotations would have led to a repeated analysis of the same structures again and again. With the database, each structure gets analysed exactly one time. The third benefit is the exportation capability of the database enabling easy shipment of StructMAn results between individual instances of the pipeline. An empty instance of the the database based on MariaDB engine is installed automatically in the docker container. For the pip version, the user needs to provide a MySQL database server. Then after the configuration of the database credentials, a single command sets up the database structure. This allows the usage of a database server that is physically apart from the system that runs the pipeline, thus enabling more effective usage of provided resources.

Local instance of the PDB

Throughout the structural analysis section of the pipeline, many thousands of PDB entries may have to be retrieved. The vanilla version takes that data directly from the RCSB webservices resulting in a lot of traffic and time delay. Many work groups oriented towards protein structure analysis already maintain an instance of the PDB locally, while other users can use a script attached to d-StructMAn that downloads and configures an instance of the PDB. The same script can be used to update the local file storage and the search index databases that are required for the sequence similarity search section of the pipeline.

Local instance of the AlphaFold Protein Structure Database

In addition to the PDB, the AlphaFold Protein Structure Database can be used as a source for protein 3D structure data. d-StructMAn contains a script that creates a local instance of the database retrieving all structures stored at https://ftp.ebi.ac.uk/pub/databases/alphafold/latest/ [44]. The database is unpacked and distributed similarly to the PDB directory scheme to ensure fast local retrieval of individual protein structures. At present, d-StructMAn offers the annotation using AlphaFold structures only when the local instance has been installed.

Local instance of a RIN database

An important part of the structural analysis is based on the RIN (residue interaction network) of the corresponding PDB entry. While a live computation of each RIN during the runtime is possible, users that plan the processing of many or large inputs may consider to precompute the RINs of all PDB entries. We provided a script that creates such a RIN database locally. Similar to the local instance of the PDB, the same script can be used to update the RIN database.

Protein sequence and protein identifier mapping database

Different protein or transcript identifiers from different sequence databases can represent an identical protein sequence. Since d-StructMAn operates on the amino acid sequence level, identifying such duplicates at the beginning of the pipeline can save a lot of computations. Uniprot hosts a comprehensive identifier mapping service (https://www.uniprot.org/uploadlists/ [45]) that can also be programmatically accessed. After checking for duplicates the sequences have to be retrieved. Both tasks require a non-blocked connection to the internet and produce some traffic, while one also depends on the Uniprot webservices to be reachable. As an optional upgrade we offer the solution to download all sequences in Uniprot and the identifier mapping tables. They then are locally processed into an SQL database for fast retrieval.

Scaling solutions

Size and complexity of inputs given to StructMAn can vary a lot. This results in various scaling issues that had to be solved in a way considering the resources provided by the underlying system architecture. We provide several solutions to optimally balance the core load and memory usage.

Input chunking

The major mechanism to control memory consumption is the input chunking that divides the input in equally-sized subtasks. The size of a chunk is defined by the number of proteins it contains. It increases when more memory is available, while it decreases when more cores are provided. Larger proteins or those that will be mapped to more structures generate more load on the system. Since the number of proteins only roughly estimates the true complexity of a subtask, the input chunking may be very tentative. The implemented parallelization techniques that are explained in the following subsection enable an efficient core usage even when the number of given proteins is lower than the number of available cores.

Parallelization of the alignment section

For each protein-to-structure mapping coming out of the initial sequence similarity search, a Needleman-Wunsch alignment without terminal gap penalties with runtime complexity $O(nm)$, where n is the length of the protein sequence and m is the length of sequence retrieved from the mapped structure, is computed. Since at this stage of the pipeline both sequence lengths are known, the necessary runtimes for each task can be estimated quite precisely. We use this to prepare N equally complex subtasks, where N is the amount of available cores. In this packaging process we also prefer putting alignment task from the same protein together in order to reduce data amounts shared to the resulting subprocesses. In summary, this leads to a packaging strategy that adapts to different types of inputs. An input carrying a single protein will distribute multiple alignments of the same protein to different structures to different subprocesses and inputs with multiple proteins combine alignments of the same protein together and assign them together to a single subprocess.

Parallelization of the structural analysis

The structural analysis is performed separately for different PDB entries. The complexity of most computations is linear by the number of protein chains in the entry, with the exception of analyses that revolve around interaction between different chains, which have a roughly quadratic complexity. While generally we can distribute the analyses of different entries to different subprocesses, this can lead to situations, where the computation time for a large entry in a separate subprocess takes longer than the computation of all entries. To avoid such scenarios, we assign multiple threads to larger structures and subdivide many of the analyses chain-wise. This is

a so-called nested parallelization and should be avoided if possible, but in our case it provided a significant performance increase in practice.

Parallelization of the results aggregation

The structural analysis results from many different structures are combined for each position, this typically requires a lot of resources to look up data from one big data structure. Such a task cannot be effectively parallelized in Python without copying the datastructure for each subprocess, which leads to an overhead that is larger than the time saved by parallelized processing. The Ray [46] library offers solutions for exactly such problem settings; however, when we increase the number of threads the overhead also increases until we reach a point where more threads do not result in any performance increase anymore. For large jobs, we implemented an advanced optimization technique which divides the major data structure at a preprocessing step and sends the substructures to individual subprocesses, which then use the usual parallelization leading again to a nested parallelization setup that can then use a large number of available cores efficiently. Since the preprocessing step also leads to a significant overhead, this technique is best applicable in situations where a large input meets large computation resources.

Availability of source code and requirements

The code is available on GitHub, `https://github.com/kalininalab/d-StructMAn`. The implementation is in Python and the code is distributed under the LGPL-2.1 licence. Easy installation is provided using containerization software: Docker or Podman.

- Project name: d-StructMAn
- Project home page: `https://github.com/kalininalab/d-StructMAn`
- Operating system: Linux
- Programming language: Python 3.8
- Other requirements: Docker or Podman
- License: LPGL-2.1
- RRID: SCR_022534
- biotools ID: d-structman

Availability of supporting data and materials

The full structural annotation of the human proteome and ClinVar mutations are openly available in the GigaScience repository, GigaDB [47].

Declarations

List of abbreviations

- 3D: three-dimensional
- DelIns: mutli-residue substitutions
- Gb: gigabyte
- HPC: high performance computing
- indels: insertions and deletions
- MUT: mutant
- PDB: the Protein Data Bank
- RAM: random access memory
- RIN: residue interaction network
- RSA: relative solvent accessible area
- WT: wild type

Consent for publication

"Not applicable"

Competing Interests

The authors declare no competing interests.

Funding

The reported study was funded by the joint RFBR and DFG research project 20-54-12008. A.G. was supported by the German Federal Ministry of Education and Research (BMBF) within the frame-work of the e:Med research and funding concept (grant SysCARE [01ZX1908A]). S.K. was supported by the IMPRS-CS graduate student fellowship and Deutsche Forschungsgemeinschaft (DFG) project number 430158625. S.K.S. was partially supported by the UdS-HIPS-Tandem Interdisciplinary Graduate School for Drug Research. O.V.K. was supported by the Klaus Faber Foundation.

Author's Contributions

A.G. devised the method and implemented the core functionality. S.K.S. and S.K. assisted with the method development and implemented the container. V.R. and O.V.K. conceived the project. All authors wrote the manuscript.

Acknowledgements

References

1. Consortium TGP. A map of human genome variation from population scale sequencing. Nature 2010 Oct;467(7319):1061–1073. `https://www.ncbi.nlm.nih.gov/pmc/articles/PMC3042601/`.
2. Eilbeck K, Quinlan A, Yandell M. Settling the score: variant prioritization and Mendelian disease. Nature Reviews Genetics 2017 Oct;18(10):599–612. `https://www.nature.com/articles/nrg.2017.52`, number: 10 Publisher: Nature Publishing Group.
3. Chen R, Mias GI, Li-Pook-Than J, Jiang L, Lam HYK, Chen R, et al. Personal Omics Profiling Reveals Dynamic Molecular and Medical Phenotypes. Cell 2012;148(6):1293–1307. `https://www.sciencedirect.com/science/article/pii/S0092867412001663`.
4. Amoah K, Hsiao YHE, Bahn JH, Sun Y, Burghard C, Tan BX, et al. Allele-specific alternative splicing and its functional genetic variants in human tissues. Genome Research 2021 Mar;31(3):359–371. `https://genome.cshlp.org/content/31/3/359`, company: Cold Spring Harbor Laboratory Press Distributor: Cold Spring Harbor Laboratory Press Institution: Cold Spring Harbor Laboratory Press Label: Cold Spring Harbor Laboratory Press Publisher: Cold Spring Harbor Lab.
5. Chong J, Buckingham K, Jhangiani S, Boehm C, Sobreira N, Smith J, et al. The Genetic Basis of Mendelian Phenotypes: Discoveries, Challenges, and Opportunities. The American Journal of Human Genetics 2015 Aug;97(2):199–215. `http://www.sciencedirect.com/science/article/pii/S0002929715002451`.
6. Landrum MJ, Lee JM, Benson M, Brown G, Chao C, Chitipiralla S, et al. ClinVar: public archive of interpretations of clinically relevant variants. Nucleic Acids Research 2016 Jan;44(Database issue):D862–D868. `https://www.ncbi.nlm.nih.gov/pmc/articles/PMC4702865/`.
7. Lek M, Karczewski KJ, Minikel EV, Samocha KE, Banks E, Fennell T, et al. Analysis of protein-coding genetic varia-

tion in 60,706 humans. Nature 2016 Aug;536(7616):285–291. https://www.nature.com/articles/nature19057.
8. Ramensky V, Bork P, Sunyaev S. Human non-synonymous SNPs: server and survey. Nucleic Acids Research 2002 Sep;30(17):3894–3900. https://academic.oup.com/nar/article/30/17/3894/2376118, publisher: Oxford Academic.
9. Adzhubei IA, Schmidt S, Peshkin L, Ramensky VE, Gerasimova A, Bork P, et al. A method and server for predicting damaging missense mutations. Nature methods 2010 Apr;7(4):248–249. https://www.ncbi.nlm.nih.gov/pmc/articles/PMC2855889/.
10. Ng PC, Henikoff S. SIFT: predicting amino acid changes that affect protein function. Nucleic Acids Research 2003 Jul;31(13):3812–3814. https://www.ncbi.nlm.nih.gov/pmc/articles/PMC168916/.
11. Thomas PD, Campbell MJ, Kejariwal A, Mi H, Karlak B, Daverman R, et al. PANTHER: A Library of Protein Families and Subfamilies Indexed by Function. Genome Research 2003 Sep;13(9):2129–2141. https://www.ncbi.nlm.nih.gov/pmc/articles/PMC403709/.
12. Yue P, Melamud E, Moult J. SNPs3D: Candidate gene and SNP selection for association studies. BMC Bioinformatics 2006 Mar;7:166. https://www.ncbi.nlm.nih.gov/pmc/articles/PMC1435944/.
13. Bromberg Y, Rost B. SNAP: predict effect of non-synonymous polymorphisms on function. Nucleic Acids Research 2007 Jun;35(11):3823–3835. https://www.ncbi.nlm.nih.gov/pmc/articles/PMC1920242/.
14. Thusberg J, Olatubosun A, Vihinen M. Performance of mutation pathogenicity prediction methods on missense variants. Human Mutation 2011;32(4):358–368. https://onlinelibrary.wiley.com/doi/abs/10.1002/humu.21445.
15. De Baets G, Van Durme J, Reumers J, Maurer-Stroh S, Vanhee P, Dopazo J, et al. SNPeffect 4.0: on-line prediction of molecular and structural effects of protein-coding variants. Nucleic Acids Research 2012 Jan;40(Database issue):D935–D939. https://www.ncbi.nlm.nih.gov/pmc/articles/PMC3245173/.
16. Katsonis P, Lichtarge O. A formal perturbation equation between genotype and phenotype determines the Evolutionary Action of protein-coding variations on fitness. Genome Research 2014 Dec;24(12):2050–2058. https://www.ncbi.nlm.nih.gov/pmc/articles/PMC4248321/.
17. Capriotti E, Calabrese R, Fariselli P, Martelli PL, Altman RB, Casadio R. WS-SNPs&GO: a web server for predicting the deleterious effect of human protein variants using functional annotation. BMC Genomics 2013 May;14(Suppl 3):S6. https://www.ncbi.nlm.nih.gov/pmc/articles/PMC3665478/.
18. Ioannidis NM, Rothstein JH, Pejaver V, Middha S, McDonnell SK, Baheti S, et al. REVEL: An Ensemble Method for Predicting the Pathogenicity of Rare Missense Variants. The American Journal of Human Genetics 2016;99(4):877–885. https://www.sciencedirect.com/science/article/pii/S0002929716303706.
19. Li J, Zhao T, Zhang Y, Zhang K, Shi L, Chen Y, et al. Performance evaluation of pathogenicity-computation methods for missense variants. Nucleic Acids Research 2018 Sep;46(15):7793–7804. https://www.ncbi.nlm.nih.gov/pmc/articles/PMC6125674/.
20. Mosca R, Tenorio-Laranga J, Olivella R, Alcalde V, Céol A, Soler-López M, et al. dSysMap: exploring the edgetic role of disease mutations. Nature Methods 2015 Mar;12(3):167–168. https://www.nature.com/articles/nmeth.3289.
21. Betts MJ, Lu Q, Jiang Y, Drusko A, Wichmann O, Utz M, et al. Mechismo: predicting the mechanistic impact of mutations and modifications on molecular interactions. Nucleic Acids Research 2015 Jan;43(2):e10. https://www.ncbi.nlm.nih.gov/pmc/articles/PMC4333368/.
22. Dehiya V, Thomas J, Sael L. Impact of structural prior knowledge in SNV prediction: Towards causal variant finding in rare disease. PLoS ONE 2018 Sep;13(9). https://www.ncbi.nlm.nih.gov/pmc/articles/PMC6161878/.
23. Konc J, Skrlj B, Erzen N, Kunej T, Janezic D. GenProBiS: web server for mapping of sequence variants to protein binding sites. Nucleic Acids Research 2017 Jul;45(Web Server issue):W253–W259. https://www.ncbi.nlm.nih.gov/pmc/articles/PMC5570222/.
24. Radusky L, Modenutti C, Delgado J, Bustamante JP, Vishnopolska S, Kiel C, et al. VarQ: A Tool for the Structural and Functional Analysis of Human Protein Variants. Frontiers in Genetics 2018 Dec;9. https://www.ncbi.nlm.nih.gov/pmc/articles/PMC6291447/.
25. Wagih O, Galardini M, Busby BP, Memon D, Typas A, Beltrao P. A resource of variant effect predictions of single nucleotide variants in model organisms. Molecular Systems Biology 2018 Dec;14(12). https://www.ncbi.nlm.nih.gov/pmc/articles/PMC6301329/.
26. Segura J, Sanchez-Garcia R, Sorzano COS, Carazo JM. 3DBIONOTES v3.0: crossing molecular and structural biology data with genomic variations. Bioinformatics 2019 Sep;35(18):3512–3513. https://academic.oup.com/bioinformatics/article/35/18/3512/5320560, publisher: Oxford Academic.
27. Gress A, Ramensky V, Büch J, Keller A, Kalinina OV. StructMAn: annotation of single-nucleotide polymorphisms in the structural context. Nucleic Acids Research 2016 Jul;44(W1):W463–468.
28. Jumper J, Evans R, Pritzel A, Green T, Figurnov M, Ronneberger O, et al. Highly accurate protein structure prediction with AlphaFold. Nature 2021 Aug;596(7873):583–589. https://www.nature.com/articles/s41586-021-03819-2, number: 7873 Publisher: Nature Publishing Group.
29. Varadi M, Anyango S, Deshpande M, Nair S, Natassia C, Yordanova G, et al. AlphaFold Protein Structure Database: massively expanding the structural coverage of protein-sequence space with high-accuracy models. Nucleic Acids Research 2021 Nov;50(D1):D439–D444. https://www.ncbi.nlm.nih.gov/pmc/articles/PMC8728224/.
30. UniProt: a hub for protein information. Nucleic Acids Research 2015 Jan;43(Database issue):D204–D212. https://www.ncbi.nlm.nih.gov/pmc/articles/PMC4384041/.
31. human in UniProtKB (6971127) | UniProt;. https://www.uniprot.org/uniprotkb/?query=human&fil=proteome%3AUP000005640+AND+organism%3A%22Homo+sapiens+%28Human%29+%5B9606%5D%22&sort=score.
32. Index of /pub/clinvar/tab_delimited;. https://ftp.ncbi.nlm.nih.gov/pub/clinvar/tab_delimited/.
33. Pruitt K, Brown G, Tatusova T, Maglott D. The Reference Sequence (RefSeq) Database. National Center for Biotechnology Information (US); 2012. https://www.ncbi.nlm.nih.gov/books/NBK21091/.
34. Erdős G, Pajkos M, Dosztányi Z. IUPred3: prediction of protein disorder enhanced with unambiguous experimental annotation and visualization of evolutionary conservation. Nucleic Acids Research 2021 05;49(W1):W297–W303. https://doi.org/10.1093/nar/gkab408.
35. Schwarz JM, Cooper DN, Schuelke M, Seelow D. MutationTaster2: mutation prediction for the deep-sequencing age. Nature Methods 2014 Apr;11(4):361–362. https://www.nature.com/articles/nmeth.2890.
36. Sundaram L, Gao H, Padigepati SR, McRae JF, Li Y, Kosmicki JA, et al. Predicting the clinical impact of human mutation with deep neural networks. Nature Genetics 2018 Aug;50(8):1161. https://www.nature.com/articles/s41588-018-0167-z.
37. Boudellioua I, Kulmanov M, Schofield PN, Gkoutos GV, Hoehndorf R. DeepPVP: phenotype-based prioritization of causative variants using deep learning. BMC Bioinformatics 2019 Feb;20(1):65. https://doi.org/10.1186/s12859-019-2633-8.

38. Gress A, Ramensky V, Kalinina OV. Spatial distribution of disease-associated variants in three-dimensional structures of protein complexes. Oncogenesis 2017 Sep;6(9):e380.
39. Steinegger M, Söding J. MMseqs2 enables sensitive protein sequence searching for the analysis of massive data sets. Nature Biotechnology 2017 Oct;35:1026–1028. https://www.nature.com/articles/nbt.3988.
40. Needleman SB, Wunsch CD. A general method applicable to the search for similarities in the amino acid sequence of two proteins. Journal of Molecular Biology 1970;48(3):443–453. https://www.sciencedirect.com/science/article/pii/0022283670900574.
41. Rost B. Twilight zone of protein sequence alignments. Protein Engineering, Design and Selection 1999 Feb;12(2):85–94. https://academic.oup.com/peds/article/12/2/85/1550637.
42. Doncheva NT, Klein K, Domingues FS, Albrecht M. Analyzing and visualizing residue networks of protein structures. Trends in Biochemical Sciences 2011 Apr;36(4):179–182. http://www.sciencedirect.com/science/article/pii/S0968000411000132.
43. Rost B, Sander C. Conservation and prediction of solvent accessibility in protein families. Proteins: Structure, Function, and Bioinformatics 1994;20(3):216–226. https://onlinelibrary.wiley.com/doi/abs/10.1002/prot.340200303.
44. Index of /pub/databases/alphafold/latest/;. https://ftp.ebi.ac.uk/pub/databases/alphafold/latest/.
45. Retrieve/ID mapping | UniProt;. https://www.uniprot.org/id-mapping/.
46. Moritz P, Nishihara R, Wang S, Tumanov A, Liaw R, Liang E, et al. Ray: A Distributed Framework for Emerging AI Applications. arXiv:171205889 [cs, stat] 2018 Sep;http://arxiv.org/abs/1712.05889, arXiv: 1712.05889.
47. Decap D, de Schaetzen van Brienen L, Larmuseau M, Costanza P, Herzeel C, Wuyts R, et al. Halvade somatic: Somatic variant calling with Apache Spark. GigaScience 2022 Jan;11:giab094. https://academic.oup.com/gigascience/article/doi/10.1093/gigascience/giab094/6505120.

Click here to access/download
Supplementary Material
human_proteome_dataset_S1.csv

Click here to access/download
Supplementary Material
ClinVar_dataset_S2.csv

Click here to access/download
Supplementary Material
feature_descriptions_S3.csv

Click here to access/download
Supplementary Material
classification_example_s4.csv

Click here to access/download
Supplementary Material
supplementary_materials.xlsx

Personal Cover
Click here to access/download;Personal Cover;d-structman_cover_OK_OK.docx

HIPS HELMHOLTZ
Institute for Pharmaceutical Research Saarland

Helmholtz-Institute for Pharmaceutical Research Saarland (HIPS)
Campus E8.1 | 66123 Saarbrücken | Germany

GigaScience
Editorial Office

Prof. Dr. Olga V. Kalinina
Head of research group for Drug Bioinformatics

Phone +49 681 98806-3600
Fax +49 681 98806-3009

olga.kalinina@helmholtz-hips.de

Your Ref.	Our Ref.	Date
		06.07.2022

Submission of a revised manuscript entitled "d-StructMAn: containerized structural annotation on the scale from genetic variants to whole proteomes"

Dear Dr. Hans Zauner,

Herewith, we would like to submit a revised version of our manuscript entitled "***d-StructMAn: containerized structural annotation on the scale from genetic variants to whole proteomes***" (GIGA-D-22-00032) as a technical note to *GigaScience*.

We have addressed all concerns raised by the reviewers and include a point-by-point response. We hope that the manuscript was improved and is now suitable for publication in GigaScience.

Yours faithfully,

Olga V. Kalinina

Saarbrücken, 06.07.2022

Helmholtz-Institute for Pharmaceutical Research Saarland
Campus E8.1
66123 Saarbrücken
Germany
www.helmholtz-hips.de

Registered Office:
Helmholtz-Zentrum
für Infektionsforschung GmbH
Inhoffenstraße 7
38124 Braunschweig

Chair of Supervisory Board:
MinDir'in Prof. Dr. Veronika von Messling,
Federal Ministry of Education and Research

Scientific Director:
Prof. Dr. Dirk Heinz

Administrative Director:
Silke Tannapfel

Registry Court:
Amtsgericht Braunschweig HRB 477
VAT Reg. No DE 11 48 15 244

St.-Nr. 13/200/24006

An institution of
HZI HELMHOLTZ Centre for Infection Research
In cooperation with
UNIVERSITÄT DES SAARLANDES

Personal Cover Click here to access/download;Personal Cover;Response to reviewers.docx

Reviewer #1: The authors describe a method of annotating single-nucleotide variants and indels with information from 3D protein structures. It is an improved version of their previous tool StructMAn and can be installed and run locally as a command line tool.

The paper describes in detail how the tool operates and how it can be installed. Indeed, it rather focuses on the technical aspects and various statistics such as the numbers of variants, proteins, and performance issues. What it fails to provide are examples of the annotations themselves, how they are presented, and how these might be useful. A list of features given in Table S3, but at least one example of the output would make it clearer what one gets from the system. It does, after all, require a significant investment of effort to install and get running, so potential users need to be able to see what they will be getting out of it at the end of the process.

Response:

We would like to thank the reviewer for their patience with the installation process. The overall installation of the whole pipeline is quite complex due to the usage of many dependencies, online resources and especially the integrated MySQL database. For the publication in GigaScience our focus was on the processing of very large inputs, and to achieve that the pipeline needs all the previously mentioned parts installed. While we plan to provide a full installation guide outside of the container in the future, at present containerization was an ideal solution to provide the full potential of the pipeline without the need of a trained systems administrator to install the tool. In the future, we also want to provide a quicker installation that limits the pipeline to the 'lite-mode'. This installation will then not be recommended to process large inputs, but can be very easily installed and used. To showcase the output capabilities of the tool, we added a section explaining the results for one example with a single amino acid substitution D833A in the histone-lysine N-methyltransferase EHMT2.

Reviewer #2: Comments

The authors describe the d-StructMAn containerized methodology and provide a comprehensive summary of its performance, with a nice reading flow, especially in the introduction. They present the performance of the tool by structurally annotating the human proteome as well as a clinically relevant database such as ClinVar. Additionally, d-StructMAn authors provide insights on important aspects that influence their performances, which is of general interest and value to the community. Regarding the implementation of their tool, they are very aware of the data load the user will have to deal with and provide nice adaptations of their software. Other important aspects of the method, in particular input formats, therholds selection and the lack of high quality structural models from AlphaFold or RosettaFold are less detailed and would benefit of some clarification.

Response:

We are very happy to receive a reviewer that has dealt with a similar topic in the past and can delve even into the finest details of this project. We support multiple types of input formats, and explaining them in details would take too much space, thus we chose to provide the full documentation in the wiki that comes with the git repository

(https://github.com/kalininalab/d-StructMAn/wiki/Simple-Mutation-List-Format). We will continue to improve the content of the wiki in the future.
We tried to give better reasonings for our selected thresholds (see point-to-point responses).
We added the support for AlphaFold models, which needed quite some time to implement and test, which is the main cause for the long amount of time we needed to submit the revised manuscript.

Major

1) The choice of using all isoforms instead of a single isoform per gene can potentially be problematic. On one hand, it is an unusual experiment and the results, therefore, are interesting. On the other hand, this biases significantly the "overall" results towards genes with more isoforms. Another issue is that this choice makes it hard to interpret the rest of the results of the paper. For example, as it is written, I cannot know whether in Table 2 the "10 proteins, very many hits" include 10 proteins from 10 different genes, 10 protein isoforms from the same gene, or any combination in between. I'd suggest that the authors also show the results of the coverage with one isoform per gene. This will also make their results easier to compare to the existing literature.

Response:

All proteins in the benchmark datasets are not isoforms of one or several genes, but rather come from different genes. We use different isoforms only in the whole human proteome experiment, but the user is free to provide one or several isoforms per gene as input. We tried to make it clearer in the text that "using different isoforms" is a matter of input, not hard-coded in the pipeline.
Analyzing one isoform per gene in the human proteome does not change the results much, since the majority of proteins in this dataset only have one isoform (78.24%), so we chose to focus on the isoform-specific analysis, since this is the unique feature of our pipeline to differentiate between different isoforms in the input Uniprot identifiers..

2) Regarding the sequence alignment: The authors do not mention why their minimum sequence homology filter is set to 35%. This is a very important choice that would alter results significantly so I believe this should be clarified in the manuscript.

Response:

We use the threshold of 35 % as a more conservative version of a commonly accepted threshold of 30-35 % that is considered sufficient sequence identity for homology-based modelling. This threshold is based on the analysis of the relationship between sequence identity and structural similarity by Burkhard Rost (doi:10.1093/protein/12.2.85).

3) Do the authors filter the sequence alignment also by evalue? If one only filters by sequence identity it is possible to retrieve many non-significant hits for short sequences. This could be an even more significant problem if including multiple short isoforms for some genes.

Response:

We do not apply filtering by e-value, since the notion of significant and non-significant hits is somewhat different here from a typical sequence search. The key principle of the method is to transfer functional conclusions from structures of related proteins, therefore some degree of redundancy is intentional. Of course, for a short isoform, we will also annotate the structures of their longer variants, since they will be returned by the sequence similarity search and aligned to the identical segments. This is intentional, since the part of the structure that the short isoform is mapped to will be structurally analyzed and the key principle of the method is to transfer functional conclusions from structures of related proteins. We have to assume here that the function of the short isoform does not change significantly, when expressed without the other domains.

4) Does it make sense to aggregate the features from multiple structures? For example, if a residue is exposed in one structure but buried in a different one due to a conformational change, the average of both (buried and exposed) is difficult to interpret from a biological perspective.

Response:

We believe that aggregating results from different structures is important and provides better biological insights. Considering every structure separately quickly becomes infeasible for well studied proteins. However, the point raised by the reviewer is a valid one and constitutes a challenge. We approached this by favoring buried/core residues in the aggregation step. There are two reasons for this approach: 1. In partially resolved structures, buried residues can appear on the surface, while in reality being buried, thus we trust an annotation as a buried residue more than as a surface residue. We take the alignment coverage into account to a greater extent when aggregating surface exposure features compared to the aggregation of other structural features. 2. Buried and core residues tend to be more important for the stability and function of the protein compared to surface residues, and the same we assume to be true for residues that are surface residues in one conformation and buried in another conformation. Thus, annotating those switching residues as buried is more useful. We added more details in text to clarify our decisions. A potential future improvement would be to detect such cases and introduce another exposure type in our classification scheme. However, we would like to refrain from commenting on mutations' functional importance in this manuscript, since it only meant to introduce the classification tool.

5) Following the same thought as in point 3, how does the decision tree for the "Structural classification" decide which feature to assign when there is more than one interaction feature (i.e. a residue that interacts with a protein in a structure and with a nucleic acid in another)?

Response:

There are two classification schemes that are used in the results of the tool. First, a more complex one, lists all different interactions as multiple interactions. For the simpler scheme, we have to choose one of the types, here we take the interaction type with the highest aggregated interaction score.

Or if a residue is exposed in one structure and accessible in another?

Response:

Please see our response to point 4 that clarifies the assignment based on surface exposure.

6) Related to the previous point, the authors do not specify the RSA threshold they use to determine if a residue is buried or not. Again, I think this would affect the structural functional annotation, and therefore the reader should be aware of.

Response:

We added the threshold and explanation of how we chose it into the text.

7) The authors do not clarify how the input of protein positions or variants is. For example, variants do not follow a standard format so I think it is of importance to specify this to the reader.

Response:

We thought the details of our input formats would be too technical for the manuscript (which is already very technical) and left the documentation for it in our wiki. While there is sadly no standard format, we tried to design our format to comply with other formats previously used in the field.

8) In section "Clinically relevant genetic variations", it is unclear what are the 123 features and the 600 for either SNVs and indels. In supplementary table S3 only includes 75 d-StructMAn features, so I guess the rest of features are ClinVar features.

Response:

We added a remark in the section "Clinically relevant genetic variations" where to find more details on the feature generation. In supplementary table S3, some feature descriptors contain brackets "[" and "]" denoting a list of alternative words that each describe a different feature. Thus, the total number of features (123) is higher than the number of rows (74) in table S3. For indels, most features come in six different variants (WT, MUT, left flank WT, left flank MUT, right flank WT, right flank MUT), which results in a total 600 features.
