# Supplementary material for: d-StructMAn: Containerized structural annotation on the scale from genetic variants to whole proteomes
Source: Gigascience. 2022 Sep 20;11:giac086. doi: 10.1093/gigascience/giac086 (PMC9487898; doi:10.1093/gigascience/giac086)
Supplement: giac086_Response_to_Reviewer_Comments_Original_Submission [file giac086_response_to_reviewer_comments_original_submission.pdf]

Author's Response To Reviewer Comments

Close

Reviewer #1: The authors describe a method of annotating single-nucleotide variants and indels with information from 3D protein structures. It is an improved version of their previous tool StructMAn and can be installed and run locally as a command line tool.

The paper describes in detail how the tool operates and how it can be installed. Indeed, it rather focuses on the technical aspects and various statistics such as the numbers of variants, proteins, and performance issues. What it fails to provide are examples of the annotations themselves, how they are presented, and how these might be useful. A list of features given in Table S3, but at least one example of the output would make it clearer what one gets from the system. It does, after all, require a significant investment of effort to install and get running, so potential users need to be able to see what they will be getting out of it at the end of the process.

Response:

We would like to thank the reviewer for their patience with the installation process. The overall installation of the whole pipeline is quite complex due to the usage of many dependencies, online resources and especially the integrated MySQL database. For the publication in GigaScience our focus was on the processing of very large inputs, and to achieve that the pipeline needs all the previously mentioned parts installed. While we plan to provide a full installation guide outside of the container in the future, at present containerization was an ideal solution to provide the full potential of the pipeline without the need of a trained systems administrator to install the tool. In the future, we also want to provide a quicker installation that limits the pipeline to the 'lite-mode'. This installation will then not be recommended to process large inputs, but can be very easily installed and used. To showcase the output capabilities of the tool, we added a section explaining the results for one example with a single amino acid substitution D833A in the histone-lysine N-methyltransferase EHMT2.

Reviewer #2: Comments

The authors describe the d-StructMAn containerized methodology and provide a comprehensive summary of its performance, with a nice reading flow, especially in the introduction. They present the performance of the tool by structurally annotating the human proteome as well as a clinically relevant database such as ClinVar. Additionally, d-StructMAn authors provide insights on important aspects that influence their performances, which is of general interest and value to the community. Regarding the implementation of their tool, they are very aware of the data load the user will have to deal with and provide nice adaptations of their software. Other important aspects of the method, in particular input formats, therholds selection and the lack of high quality structural models from AlphaFold or RosettaFold are less detailed and would benefit of some clarification.

Response:

We are very happy to receive a reviewer that has dealt with a similar topic in the past and can delve even into the finest details of this project. We support multiple types of input formats, and explaining them in details would take too much space, thus we chose to provide the full documentation in the wiki that comes with the git repository (https://github.com/kalininalab/d-StructMAn/wiki/Simple-Mutation-List-Format). We will continue to improve the content of the wiki in the future.
We tried to give better reasonings for our selected thresholds (see point-to-point responses).
We added the support for AlphaFold models, which needed quite some time to implement and test, which is the main cause for the long amount of time we needed to submit the revised manuscript.

Major

1) The choice of using all isoforms instead of a single isoform per gene can potentially be problematic. On one hand, it is an unusual experiment and the results, therefore, are interesting. On the other hand,

this biases significantly the "overall" results towards genes with more isoforms. Another issue is that this choice makes it hard to interpret the rest of the results of the paper. For example, as it is written, I cannot know whether in Table 2 the "10 proteins, very many hits" include 10 proteins from 10 different genes, 10 protein isoforms from the same gene, or any combination in between. I'd suggest that the authors also show the results of the coverage with one isoform per gene. This will also make their results easier to compare to the existing literature.

Response:

All proteins in the benchmark datasets are not isoforms of one or several genes, but rather come from different genes. We use different isoforms only in the whole human proteome experiment, but the user is free to provide one or several isoforms per gene as input. We tried to make it clearer in the text that "using different isoforms" is a matter of input, not hard-coded in the pipeline.
Analyzing one isoform per gene in the human proteome does not change the results much, since the majority of proteins in this dataset only have one isoform (78.24%), so we chose to focus on the isoform-specific analysis, since this is the unique feature of our pipeline to differentiate between different isoforms in the input Uniprot identifiers..

2) Regarding the sequence alignment: The authors do not mention why their minimum sequence homology filter is set to 35%. This is a very important choice that would alter results significantly so I believe this should be clarified in the manuscript.

Response:

We use the threshold of 35 % as a more conservative version of a commonly accepted threshold of 30-35 % that is considered sufficient sequence identity for homology-based modelling. This threshold is based on the analysis of the relationship between sequence identity and structural similarity by Burkhard Rost (doi:10.1093/protein/12.2.85).

3) Do the authors filter the sequence alignment also by evalue? If one only filters by sequence identity it is possible to retrieve many non-significant hits for short sequences. This could be an even more significant problem if including multiple short isoforms for some genes.

Response:

We do not apply filtering by e-value, since the notion of significant and non-significant hits is somewhat different here from a typical sequence search. The key principle of the method is to transfer functional conclusions from structures of related proteins, therefore some degree of redundancy is intentional. Of course, for a short isoform, we will also annotate the structures of their longer variants, since they will be returned by the sequence similarity search and aligned to the identical segments. This is intentional, since the part of the structure that the short isoform is mapped to will be structurally analyzed and the key principle of the method is to transfer functional conclusions from structures of related proteins. We have to assume here that the function of the short isoform does not change significantly, when expressed without the other domains.

4) Does it make sense to aggregate the features from multiple structures? For example, if a residue is exposed in one structure but buried in a different one due to a conformational change, the average of both (buried and exposed) is difficult to interpret from a biological perspective.

Response:

We believe that aggregating results from different structures is important and provides better biological insights. Considering every structure separately quickly becomes infeasible for well studied proteins. However, the point raised by the reviewer is a valid one and constitutes a challenge. We approached this by favoring buried/core residues in the aggregation step. There are two reasons for this approach: 1. In partially resolved structures, buried residues can appear on the surface, while in reality being buried, thus we trust an annotation as a buried residue more than as a surface residue. We take the alignment coverage into account to a greater extent when aggregating surface exposure features compared to the aggregation of other structural features. 2. Buried and core residues tend to be more important for the stability and function of the protein compared to surface residues, and the same we assume to be true for residues that are surface residues in one conformation and buried in another conformation. Thus, annotating those switching residues as buried is more useful. We added more details in text to clarify

our decisions. A potential future improvement would be to detect such cases and introduce another exposure type in our classification scheme. However, we would like to refrain from commenting on mutations' functional importance in this manuscript, since it only meant to introduce the classification tool.

5) Following the same thought as in point 3, how does the decision tree for the "Structural classification" decide which feature to assign when there is more than one interaction feature (i.e. a residue that interacts with a protein in a structure and with a nucleic acid in another)?

Response:

There are two classification schemes that are used in the results of the tool. First, a more complex one, lists all different interactions as multiple interactions. For the simpler scheme, we have to choose one of the types, here we take the interaction type with the highest aggregated interaction score.

Or if a residue is exposed in one structure and accessible in another?

Response:

Please see our response to point 4 that clarifies the assignment based on surface exposure.

6) Related to the previous point, the authors do not specify the RSA threshold they use to determine if a residue is buried or not. Again, I think this would affect the structural functional annotation, and therefore the reader should be aware of.

Response:

We added the threshold and explanation of how we chose it into the text.

7) The authors do not clarify how the input of protein positions or variants is. For example, variants do not follow a standard format so I think it is of importance to specify this to the reader.

Response:

We thought the details of our input formats would be too technical for the manuscript (which is already very technical) and left the documentation for it in our wiki. While there is sadly no standard format, we tried to design our format to comply with other formats previously used in the field.

8) In section "Clinically relevant genetic variations", it is unclear what are the 123 features and the 600 for either SNVs and indels. In supplementary table S3 only includes 75 d-StructMAn features, so I guess the rest of features are ClinVar features.

Response:

We added a remark in the section "Clinically relevant genetic variations" where to find more details on the feature generation. In supplementary table S3, some feature descriptors contain brackets "[" and "]" denoting a list of alternative words that each describe a different feature. Thus, the total number of features (123) is higher than the number of rows (74) in table S3. For indels, most features come in six different variants (WT, MUT, left flank WT, left flank MUT, right flank WT, right flank MUT), which results in a total 600 features.

Close
