# Supplementary material for: d-StructMAn: Containerized structural annotation on the scale from genetic variants to whole proteomes
Source: Gigascience. 2022 Sep 20;11:giac086. doi: 10.1093/gigascience/giac086 (PMC9487898; doi:10.1093/gigascience/giac086)
Supplement: giac086_Response_to_Reviewer_Comments_Revision_1 [file giac086_response_to_reviewer_comments_revision_1.pdf]

Author's Response To Reviewer Comments

Close

We would like to thank the reviewers for the fair process and for accepting our manuscript.

Close
