# Supplementary material for: d-StructMAn: Containerized structural annotation on the scale from genetic variants to whole proteomes
Source: Gigascience. 2022 Sep 20;11:giac086. doi: 10.1093/gigascience/giac086 (PMC9487898; doi:10.1093/gigascience/giac086)
Supplement: giac086_Reviewer_2_Report_Original_Submission — Eduard Porta Pardo -- 4/5/2022 Reviewed [file giac086_reviewer_2_report_original_submission.pdf]

Reviewer Report

Title: d-StructMAn: containerized structural annotation on the scale from genetic variants to whole proteomes

Version: Original Submission **Date:** 4/5/2022

Reviewer name: Eduard Porta Pardo

Reviewer Comments to Author:

Comments
The authors describe the d-StructMAn containerized methodology and provide a comprehensive summary of its performance, with a nice reading flow, especially in the introduction. They present the performance of the tool by structurally annotating the human proteome as well as a clinically relevant database such as ClinVar. Additionally, d-StructMAn authors provide insights on important aspects that influence their performances, which is of general interest and value to the community. Regarding the implementation of their tool, they are very aware of the data load the user will have to deal with and provide nice adaptations of their software. Other important aspects of the method, in particular input formats, therholds selection and the lack of high quality structural models from AlphaFold or RosettaFold are less detailed and would benefit of some clarification.
Major
1) The choice of using all isoforms instead of a single isoform per gene can potentially be problematic. On one hand, it is an unusual experiment and the results, therefore, are interesting. On the other hand, this biases significantly the "overall" results towards genes with more isoforms. Another issue is that this choice makes it hard to interpret the rest of the results of the paper. For example, as it is written, I cannot know whether in Table 2 the "10 proteins, very many hits" include 10 proteins from 10 different genes, 10 protein isoforms from the same gene, or any combination in between. I'd suggest that the authors also show the results of the coverage with one isoform per gene. This will also make their results easier to compare to the existing literature.
2) Regarding the sequence alignment: The authors do not mention why their minimum sequence homology filter is set to 35%. This is a very important choice that would alter results significantly so I believe this should be clarified in the manuscript.
3) Do the authors filter the sequence alignment also by evalue? If one only filters by sequence identity it is possible to retrieve many non-significant hits for short sequences. This could be an even more significant problem if including multiple short isoforms for some genes.
4) Does it make sense to aggregate the features from multiple structures? For example, if a residue is exposed in one structure but buried in a different one due to a conformational change, the average of both (buried and exposed) is difficult to interpret from a biological perspective.
5) Following the same thought as in point 3, how does the decision tree for the "Structural classification" decide which feature to assign when there is more than one interaction feature (i.e. a residue that interacts with a protein in a structure and with a nucleic acid in another)? Or if a residue is exposed in one structure and accessible in another?

6) Related to the previous point, the authors do not specify the RSA threshold they use to determine if a residue is buried or not. Again, I think this would affect the structural functional annotation, and therefore the reader should be aware of.
7) The authors do not clarify how the input of protein positions or variants is. For example, variants do not follow a standard format so I think it is of importance to specify this to the reader.
8) In section "Clinically relevant genetic variations", it is unclear what are the 123 features and the 600 for either SNVs and indels. In supplementary table S3 only includes 75 d-StructMAn features, so I guess the rest of features are ClinVar features.
Eduard Porta Pardo
Victoria Ruiz Serra

Level of Interest

Please indicate how interesting you found the manuscript: Choose an item.

Quality of Written English

Please indicate the quality of language in the manuscript: Choose an item.

Declaration of Competing Interests

Please complete a declaration of competing interests, considering the following questions:

- Have you in the past five years received reimbursements, fees, funding, or salary from an organisation that may in any way gain or lose financially from the publication of this manuscript, either now or in the future?
- Do you hold any stocks or shares in an organisation that may in any way gain or lose financially from the publication of this manuscript, either now or in the future?
- Do you hold or are you currently applying for any patents relating to the content of the manuscript?
- Have you received reimbursements, fees, funding, or salary from an organization that holds or has applied for patents relating to the content of the manuscript?
- Do you have any other financial competing interests?
- Do you have any non-financial competing interests in relation to this paper?

If you can answer no to all of the above, write 'I declare that I have no competing interests' below. If your reply is yes to any, please give details below.

We declare that we have no competing financial interests. We have been developing a similar tool to the one described in this manuscript for the last four years and we will soon submit a manuscript describing it.

I agree to the open peer review policy of the journal. I understand that my name will be included on my report to the authors and, if the manuscript is accepted for publication, my named report including any attachments I upload will be posted on the website along with the authors' responses. I agree for my report to be made available under an Open Access Creative Commons CC-BY license (http://creativecommons.org/licenses/by/4.0/). I understand that any comments which I do not wish to be included in my named report can be included as confidential comments to the editors, which will not be published.

Choose an item.

To further support our reviewers, we have joined with Publons, where you can gain additional credit to further highlight your hard work (see: https://publons.com/journal/530/gigascience). On publication of this paper, your review will be automatically added to Publons, you can then choose whether or not to claim your Publons credit. I understand this statement.

Yes Choose an item.
